# Efficacy of Vitamins on Cognitive Function of Non-Demented People: A Systematic Review and Meta-Analysis

**DOI:** 10.3390/nu12041168

**Published:** 2020-04-22

**Authors:** Seung Wan Suh, Hye Sung Kim, Ji Hyun Han, Jong Bin Bae, Dae Jong Oh, Ji Won Han, Ki Woong Kim

**Affiliations:** 1 Department of Psychiatry, College of Medicine, Hallym University, Kangdong Sacred Heart Hospital, Seoul 05355, Korea; 2 Department of Neuropsychiatry, Seoul National University Bundang Hospital, Seongnam 13620, Korea; 3 Department of Psychiatry, College of Medicine, Seoul National University, Seoul 03080, Korea; 4 Department of Brain and Cognitive Sciences, College of Natural Sciences, Seoul National University, Seoul 08826, Korea

**Keywords:** B vitamins, antioxidants, vitamin D, cognitive

## Abstract

Previous evidence has suggested that vitamins might be beneficial for cognition. This systematic review aimed to investigate the efficacy of B vitamins, antioxidant vitamins, and vitamin D on the cognitive function of non-demented middle-aged or older people. Randomized or quasi-randomized controlled trials of individuals aged 40 years or older were included. PubMed/MEDLINE, EMBASE, CINAHL, PsycINFO, Cochrane Library databases, and other grey literature sources were searched up to November 2019. Their methodological quality was evaluated using the Cochrane Risk of Bias tool. Twenty-three studies on B vitamins (*n =* 22–1053; comprising folate, B6, and B12), nine on antioxidant vitamins (*n =* 185–20,469), and six on vitamin D (*n =* 55–4122) were included. Taking B vitamins for over 3 months was beneficial for global cognition (standardized mean difference (SMD) −0.18, 95% CI −0.30 to −0.06) and episodic memory (SMD −0.09, 95% CI −0.15 to −0.04). However, antioxidant vitamins (SMD −0.02, 95% CI −0.08 to 0.03) and vitamin D (SMD −0.06, 95% CI −0.36 to 0.23) were not. Antioxidant vitamins were beneficial for global cognition in sensitivity analyses using final measurement data as mean difference estimates (SMD, −0.04, 95% CI −0.08 to −0.01). Taking B vitamins and possibly antioxidant vitamins may be beneficial for the cognitive function of non-demented people.

## 1. Introduction

Many previous studies have reported that vitamins have a modifying effect on cognitive function. For example, B vitamins, especially folate, vitamin B6, and vitamin B12, are involved in eliminating homocysteine from the body [1], which has been related to dementia via direct neurotoxic or vascular mechanisms [2,3]. In our previous cohort study, a normal-but-low level of serum folate was associated with the risk of dementia [4]. Vitamin C, vitamin E, and carotenoids ameliorated oxidative stress that could cause or mediate the neurodegenerative process [5]. Vitamin D decreased amyloid β production, increased amyloid β clearance, and protected neurons from inflammation damage.

However, the effects of vitamins on cognitive function of non-demented elderly have been inconsistent even in meta-analyses on randomized or quasi-randomized, placebo-controlled trials [6,7,8,9,10,11,12,13,14]. These conflicting results may be attributable to several limitations of previous meta-analyses. Firstly, the proportion of the subjects with mild cognitive impairment (MCI) was not uniform across the studies, and the effect of vitamins on cognitive function may be different between subjects with normal cognition and those with MCI [6,7,8,9,10,11,12,13,14]. Secondly, the effect of different geographical locations or ethnicities was not considered in all previous meta-analyses, even though they are related to dietary habits and genetic backgrounds. Thirdly, most previous meta-analyses did not examine the isolated effects of vitamins by allowing the inclusion of minerals or other cofactors as intervention material [6,7,8,12,14].

In this systematic review, we evaluated the efficacy of vitamins without minerals or other cofactors on cognitive function of non-demented people and compared these results by geographical location and the presence of MCI.

## 2. Materials and Methods

In this review, we followed the Preferred Reporting Items for Systematic Reviews and Meta-Analyses (PRISMA) guidelines [15]. The study protocol was registered at the International Prospective Register of Systematic Reviews (PROSPERO, registration number: CRD42020151379).

### 2.1. Criteria for Study Inclusion/Exclusion

We included studies that met the following criteria: (1) investigated humans aged 40 years or older; (2) designed as a randomized or quasi-randomized placebo-controlled clinical trial, published or unpublished, without any restrictions on the publication date; (3) written in any language; (4) examined the effect of B vitamins, antioxidant vitamins (C, A, E), or vitamin D which were orally or parenterally administered; (5) used validated cognitive test scores as outcome measures. We excluded studies when (1) they included subjects with dementia or a major neurocognitive disorder according to Diagnostic and Statistical Manual for Mental Disorders (DSM)-III-R criteria, DSM-IV criteria, DSM-IV-TR criteria, DSM-5 criteria, International Classification of Diseases (ICD)-9, ICD-9-Clinical Modification (CM), or ICD-10-CM at the baseline evaluation; (2) they included subjects with severe psychiatric or neurological disorders involving apparent cognitive sequelae at the baseline evaluation; (3) the duration of intervention was less than 3 months; (4) the intervention materials included nutrients other than vitamins (for example, minerals, cofactors, or amino acids) unless the study design analyzed the effect of these vitamins separately; (5) they represented additional trials on the same participants included in a previous trial.

### 2.2. Search Strategy and Study Selection

SW Suh, JH Han, and DJ Oh conducted electronic searches of the PubMed/MEDLINE, EMBASE, CINAHL, PsycINFO, and Cochrane Library databases from inception to November 2019. During the same period, we also searched for grey literature through the Open Grey database and the World Health Organization portal which covers ClinicalTrials.gov. A search strategy was developed for each of the databases based on previous literature [6], and detailed search keywords are provided in the Supplementary Materials (Table S1).

Two geropsychiatrists, SW Suh and JH Han, independently screened titles and abstracts obtained from the searches, followed by full-text evaluation. For those with non-English texts, they planned to use Google Translate to convert them into English. When a disagreement arose regarding the screening results, KW Kim and JW Han made a ruling unless it was resolved by a discussion between SW Suh and JH Han.

### 2.3. Outcomes

As the primary outcome of this study, we used the mean difference between baseline and follow-up assessments of validated cognitive test scores on global cognitive function. As secondary outcomes of this study, we employed the mean difference between baseline and follow-up assessments of validated cognitive test scores on episodic memory, executive function, processing speed, attention, and visuospatial function. If a study evaluated a specific cognitive function using two or more cognitive tests simultaneously, we included the more frequently employed test for a particular cognitive domain among other studies [6]. When two or more cognitive tests were equally employed among the included studies, we chose one test that was more representative of the particular cognitive domain.

### 2.4. Data Extraction and Assessment of the Methodological Quality

Two investigators (SW Suh and HS Kim) extracted data independently based on a standardized template that included the study design, location, setting of recruitment and treatment, sample size, baseline participant characteristics (age, female proportion, and cognitive function), inclusion/exclusion criteria, methods of intervention and placebo arms (composition, dosage, frequency, and period), cognitive outcome measures and their numerical data, funding sources, and information on treatment adherence. If cognitive test scores of a given cognitive domain were reported at multiple time points, we extracted the longest available one within a pre-specified timeframe, such as 3 to 11 months and 12 or more months. In addition, if a trial reported outcomes separately for several vitamins belonging to the same supplementation category (B vitamins, antioxidant vitamins, and vitamin D), we extracted data from the vitamin that most frequently appeared among the included trials. When the numerical data of cognitive test scores including means and measures of dispersion were not reported, we approximated them from available figures. For trials with a crossover design, we used data only from the first intervention period.

The two investigators used the Risk of Bias (RoB) Tool from the Cochrane Handbook to evaluate the RoB in terms of random sequence generation (selection bias), allocation concealment (selection bias), blinding of participants and personnel (performance bias), blinding of outcome assessment (detection bias), incomplete outcome data (attrition bias), selective reporting (reporting bias), and other biases [16]. If there was a disagreement regarding the extracted data or RoB that was unable to be resolved through a discussion, other reviewers (KW Kim and JW Han) made a decision. We requested unpublished or missing data that we needed for the analyses from the corresponding authors.

### 2.5. Statistical Analyses

We ran standard inverse-variance random-effects meta-analysis to synthesize data across studies [17]. For continuous outcomes, a mean difference (MD) was used as the measure of treatment effect if all studies used the same cognitive test, and if not, a standardized mean difference (SMD) was used instead. The scales of cognitive tests were made to have a consistent direction of effect across all included studies, with negative estimates favoring intervention groups and positive estimates favoring placebo groups. The results of the meta-analyses were presented as forest plots. To evaluate between-trial heterogeneity, we visually examined forest plots for its presence, and computed the I^2^ value and Chi^2^ test [18].

We conducted a-priori subgroup analyses for the primary outcome which include: by the number of months in the intervention period (3 to 11 versus ≥ 12 months), by the geographical location of the study (North America/Europe/Australia versus Asia/Middle East), or by baseline cognitive function (cognitively normal versus MCI). To evaluate the robustness of results, we performed several sensitivity analyses that involved (1) using final measurement data as the mean difference estimates of the cognitive test because the change scores could lead to an exaggerated outcome [19]; (2) eliminating studies with two or more aspects of bias rated as high risk or any literature that was not formally published as a full article in books or journal articles; (3) excluding trials of which eligibility criteria permitted the inclusion of those with a preexisting condition (for example, cerebrovascular disease or diabetes mellitus); and (4) excluding trials that restricted the baseline level of vitamins or their related material of the study participants. Analyses were performed using Review Manager 5.3 [20], and we used P values with two-sided significance and 95% confidence intervals. We produced funnel plots to visually examine the evidence of publication bias. When the number of included trials for a given meta-analysis was 10 or more, Egger’s test [21] with a significance level of < 0.05 was applied to quantitatively measure the degree of publication bias using R Statistical Software (version 3.5.1; R Foundation for Statistical Computing, Vienna, Austria).

## 3. Results

### 3.1. Study Selection

**Figure 1:**
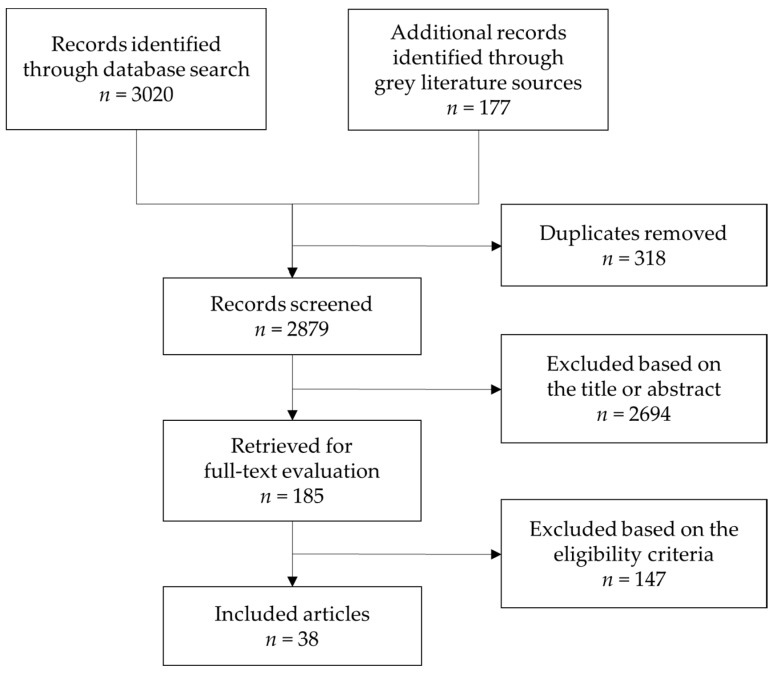
Preferred Reporting Items for Systematic Reviews and Meta-Analyses (PRISMA) flow chart of the study selection process.

Of the 3197 papers retrieved, 318 were duplicates, and 2694 articles were excluded by screening their titles and abstracts. All of the non-English papers retrieved at baseline had either their title or abstract written in English, and all of them were excluded based on this information. An additional 147 articles were also eliminated through full-text assessment (not a randomized or placebo-controlled design, *n* = 62; the intervention material contained nutrients other than vitamins, *n* = 46; included participants with dementia or severe alcohol use disorder at baseline, *n* = 19; duration of the intervention period was less than 3 months, *n* = 8; included participants aged less than 40 years old, *n* = 7; used study samples not independent from the already included articles, *n* = 3; and cognitive test scores were not provided, *n* = 2). As for those studies for which the cognitive test scores were not available, we did not receive any response from the corresponding authors. Therefore, 39 articles were used for the final systematic review (Figure 1).

### 3.2. Study Characteristics

Individual study characteristics are described in Supplementary Table S2.

#### 3.2.1. B Vitamins

Twenty-three studies with 6906 participants focused on B vitamins were included. Of these, one was a quasi-experimental pre-test–post-test control group design [22], one was a placebo-controlled trial allocated on matching principle [23], and the others were randomized, placebo-controlled designs. Two were letters to the editor [24,25], one was printed in proceedings [26] and the others were full articles formally published in journals. Sample sizes ranged from 22 to 1053. The length of the intervention period ranged from 3 to 72 months (mean (standard deviation, SD), 22.0 (18.7) months). Five studies included participants with cerebrovascular disease [24,27,28,29,30], and one study with diabetes mellitus [31]. We identified one study [30] that reported only the final measurement data of cognitive test scores without their baseline data.

As for the dosage and ingredient of B vitamin supplementation, 13 studies reported the use of folate (400 to 5000 µg), vitamin B6 (3 to 50 mg), and vitamin B12 (20 to 1000 µg) simultaneously [22,23,24,26,28,29,30,32,33,34,35,36,37], with two of them using riboflavin as well (10 and 25 mg, respectively) [26,29]. Four studies used folate (400 to 5000 µg) and vitamin B12 (25 to 1500 µg) concurrently [27,38,39,40]. The other two studies employed folate only (400 and 800 µg, respectively) [41,42], while another study employed vitamin B6 only (20 mg) [43]. The remaining three studies employed vitamin B12 only (all used 1000 µg) [25,31,44]. As for sex distribution, one study had only females [28], and two studies included only males [33,43]. Four studies included individuals younger than 60 years old [23,30,36,41]. One study examined participants with normal cognitive function at baseline [25] while another five studies investigated those with MCI at baseline [24,27,32,37,42]. The remaining ten studies included non-demented individuals in their trials without a clear demarcation between normal cognition and MCI. Concerning the baseline level of B vitamin and its related material, two studies explicitly targeted those with mild to moderate vitamin B12 deficiency (serum vitamin B12 level approximately between 100 and 200 pmol/L as one of the deficiency criteria) [38,44], and seven studies included those with elevated homocysteine concentration (threshold ranged from 11 to 16 µmol/L) [23,27,32,35,36,39,41].

#### 3.2.2. Antioxidant Vitamins

Of the nine studies involving 34,318 participants that dealt with antioxidant vitamins, all assumed a randomized, placebo-controlled design. Because two studies had identical baseline samples but reported different cognitive outcomes, they were treated as a single study, but incorporated in the separate meta-analyses [45,46]. Sample sizes ranged from 185 to 20,469. The length of the intervention period ranged from 12 to 216 months (mean (SD), 67.4 (65.4)). As for the dosage and ingredient of antioxidant vitamin supplementation, three studies reported the use of 300 to 2000 IU of alpha-tocopherol daily [47,48,49], one study used 50 mg beta-carotene on alternate days [50], one study used 300 mg of dl-alpha-tocopherol and 400 mg vitamin C daily [51], and the others used 268 to 600 mg alpha-tocopherol, 250 to 500 mg vitamin C, and 12 to 20 mg beta-carotene concurrently [45,46,52,53]. Regarding sex distribution, two studies had only females [47,49] while another study recruited only males [50]. Four studies included individuals younger than 60 years old [47,48,52,53]. Two studies evaluated those with MCI at baseline only [48,51]. We identified four studies [49,50,52,53] that reported only the final measurement data of cognitive test scores without their baseline data. No trials had any restrictions on the baseline level of antioxidant vitamins of study participants.

#### 3.2.3. Vitamin D

Of the six studies involving 4992 participants that dealt with vitamin D, one was a randomized pre-test–post-test design with a nonequivalent control group [54]. We identified one material that was published in conference proceedings [55]. Sample sizes ranged from 55 to 4122. The length of follow-up ranged from 3 to 96 months (mean (SD), 26.2 (36.4)). All included trials used only vitamin D3 with its dosage varied from 400 to 3600 IU daily. As for sex distribution, two studies had only females [56,57]. One study included individuals younger than 60 years old [58]. One study evaluated those with MCI at baseline only [59], and one study included participants with cerebrovascular disease [58]. Concerning the baseline level of vitamin D, three studies recruited those with a decreased level of serum 25(OH)D concentration, with their cut-points ranging from 16.8 to 26 ng/mL [54,56,58].

### 3.3. Cognitive Test Outcomes

A detailed description of the cognitive tests is presented in the Supplementary Materials (Table S3). We found that the most frequently used neuropsychological test for each cognitive domain was the mini-mental state examination (MMSE) for global cognition [22,24,25,32,33,35,37,39,48,51,54,56,57], delayed recall from California Verbal Learning Test (CVLT) for episodic memory [25,33,44,57], verbal fluency for executive function [24,28,32,35,37,38,39,41,44,47,49,50,53,57], digit symbol substitution/modality/coding test for processing speed [29,34,36,37,39,41,42,44,58,59], digit span backward for attention [24,34,38,39,42,53,57,59], and block design for visuospatial function [34,36,42,59].

### 3.4. Methodological Quality

Supplementary Figure S1 depicts our judgment about the RoB for the included studies, and its supporting evidence is detailed in the Supplementary Table S2. Regarding selection bias, two studies allocated participants in each intervention arm on matching principle [23,43], one study explicitly stated that the participants were allocated on the basis of the admitted facility [22], and another study did not conceal the allocation [42]. Therefore, these were considered to have a high risk of selection bias. Three studies did not employ placebo pills and rather used a conventional treatment or no treatment for a control group, and were thus considered to be at high risk of performance or detection bias [22,27,42]. Seven studies were judged to be vulnerable to attrition bias [23,24,25,29,43,47,48], and four studies were deemed not to have reported all the outcomes provided in the protocol [29,40,43,55].

### 3.5. Intervention Effects of B Vitamins

**Figure 2:**
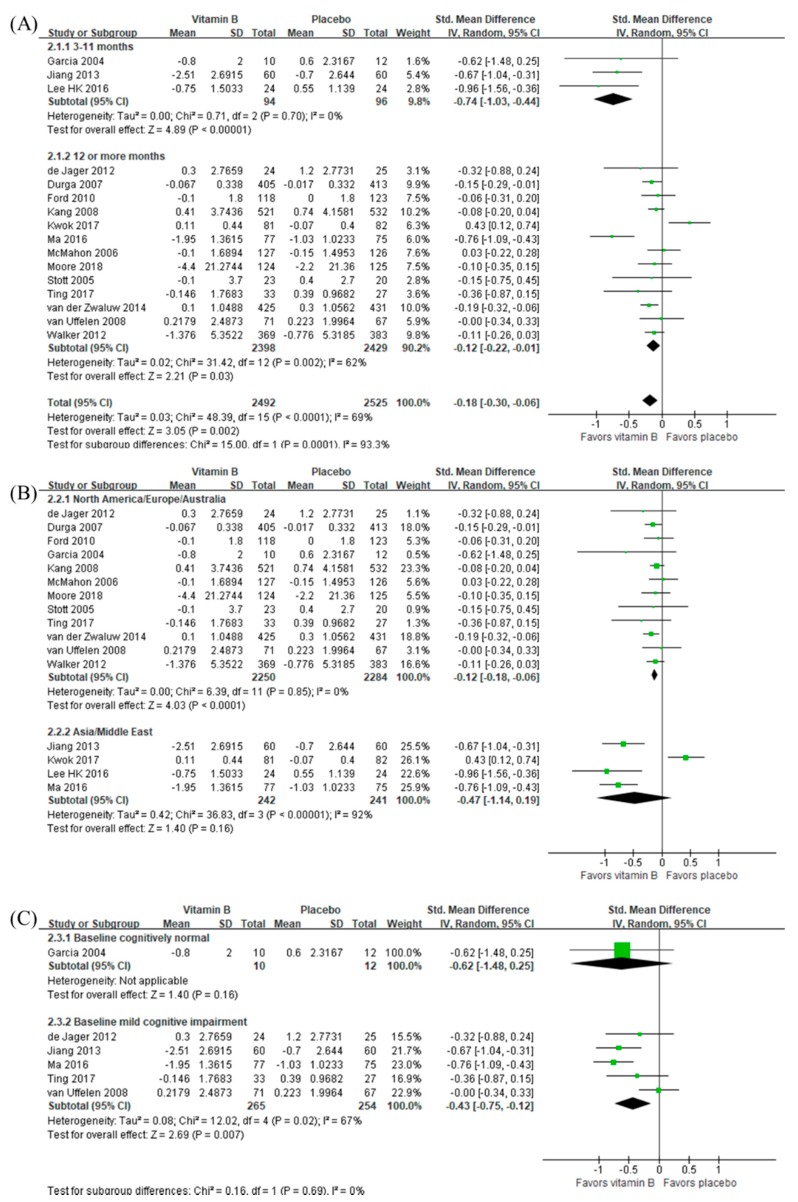
Effect of B vitamins on global cognition in terms of change from baseline by (**A**) the length of the intervention period, (**B**) the geographic location of the study, and (**C**) baseline cognitive function.

As summarized in Figure 2, B vitamins were beneficial to global cognitive function (SMD −0.18, 95% CI −0.30 to −0.06, *n =* 5017), which was the case in both short (3–11 months) and long (≥12 months) interventions, although the magnitude of the effect was larger in the former (SMD −0.74, 95% CI −1.03 to −0.44, *n =* 190) compared to the latter (SMD −0.12, 95% CI −0.22 to −0.01, *n =* 4827). Interestingly, B vitamins were beneficial to global cognitive function in the participants living in North America/Europe/Australia (SMD −0.12, 95% CI −0.18 to −0.06, *n =* 4534) with low between-trial heterogeneity (tau^2^ = 0.00 and I^2^ = 0 %) while their effect was nonsignificant in those living in Asia/Middle East (SMD −0.47, 95% CI −1.14 to 0.19, *n =* 483), with a substantially high between-trial heterogeneity (tau^2^ = 0.42 and I^2^ = 92%). When we analyzed the participants with MCI only, B vitamins were beneficial to global cognitive function (SMD −0.43, 95% CI −0.75 to −0.12, *n =* 519). There was only one study on the participants with normal cognition, in which B vitamins were not beneficial to global cognitive function (SMD −0.62, 95% CI −1.48 to 0.25, *n =* 22). After using final measurement data as mean difference estimates, we found that the beneficial effect of B vitamins on global cognition over a long period (≥12 months) became insignificant (SMD −0.07, 95% CI −0.18 to 0.03, *n =* 5176) (Supplementary Figure S2).

**Figure 3:**
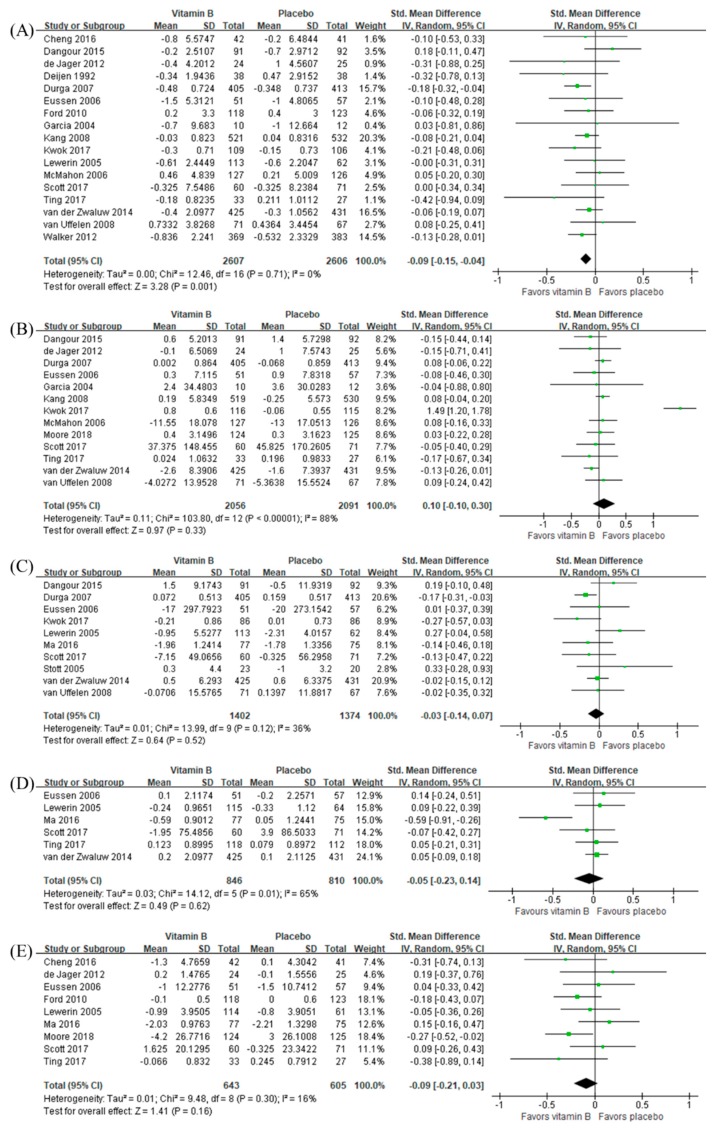
Effects of B vitamins on (**A**) episodic memory, (**B**) executive function, (**C**) processing speed, (**D**) attention, and (**E**) visuospatial function in terms of change from baseline.

As summarized in Figure 3, B vitamins were beneficial to episodic memory (SMD −0.09, 95% CI −0.15 to 0−0.04, *n =* 4213) but not to other cognitive domains (SMD 0.10, 95% CI −0.10 to 0.30, *n =* 4147 for executive function; SMD −0.03, 95% CI −0.14 to 0.07, *n =* 2776 for processing speed; SMD −0.05, 95% CI −0.23 to 0.14, *n =* 1656 for attention; SMD −0.09, 95% CI −0.21 to 0.03, *n =* 1248 for visuospatial function). Sensitivity analyses using final measurement data as mean difference estimates did not change overall results (Supplementary Figure S3).

### 3.6. Intervention Effects of Antioxidant Vitamins and Vitamin D

**Figure 4:**
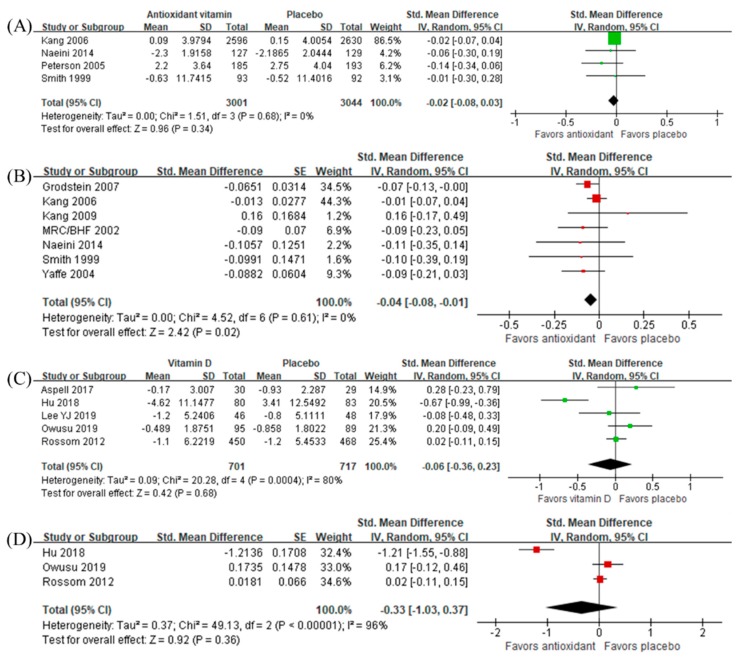
Effect of antioxidant vitamins on global cognition in terms of (**A**) change from baseline and (**B**) final measurement, and the effect of vitamin D on global cognition in terms of (**C**) change from baseline and (**D**) final measurement.

Both antioxidant vitamins (SMD −0.02, 95% CI −0.08 to 0.03, *n =* 6045, Figure 4A) and vitamin D (SMD −0.06, 95% CI −0.36 to 0.23, *n =* 1418, Figure 4C) were not beneficial to global cognitive function. However, sensitivity analyses using the final measurement data as mean difference estimates found that the antioxidant vitamins had a beneficial effect (SMD, −0.04, 95% CI −0.08 to −0.01, *n =* 2519, Figure 4B) while vitamin D did not.

**Figure 5:**
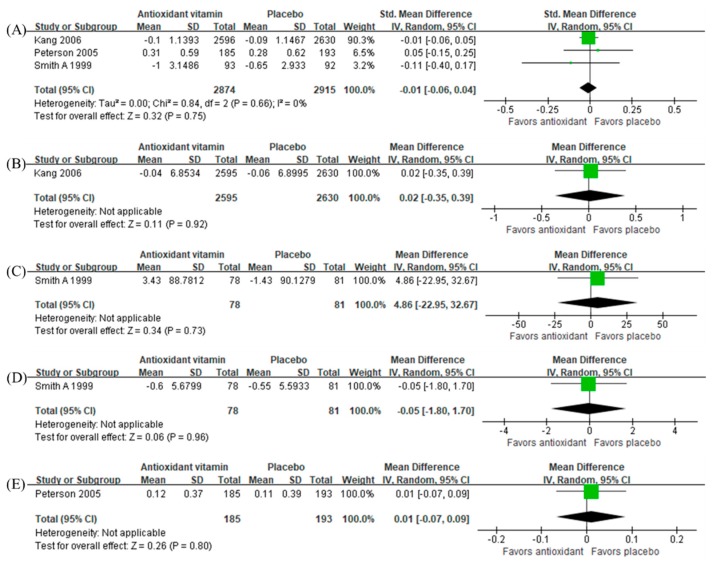
Effect of antioxidant vitamins on (**A**) episodic memory, (**B**) executive function, (**C**) processing speed, (**D**) attention, and (**E**) visuospatial function in terms of change from baseline.

**Figure 6:**
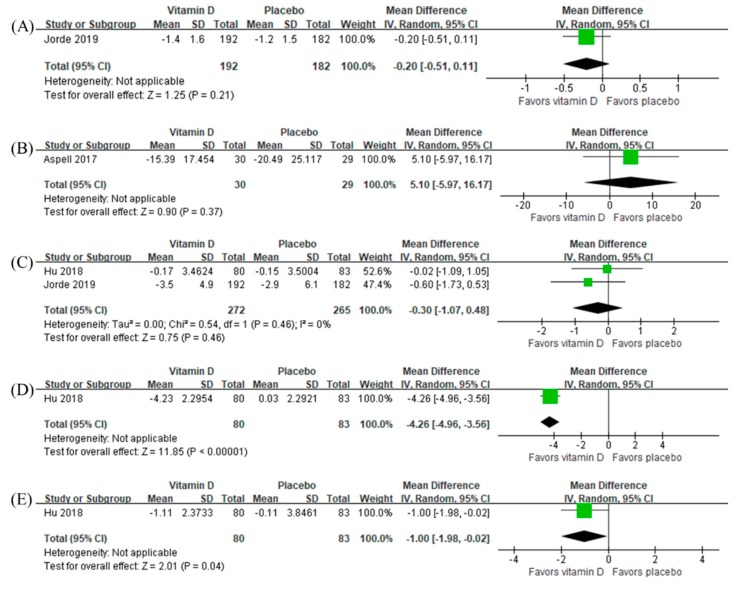
Effect of vitamin D on (**A**) episodic memory, (**B**) executive function, (**C**) processing speed, (**D**) attention, and (**E**) visuospatial function in terms of change from baseline.

In addition, antioxidant vitamins were not beneficial to all cognitive domains (Figure 5). There was only one study on the effect of vitamin D [59] in which the intervention was beneficial to attention (MD −4.26, 95% CI −4.96 to −3.56, *n =* 163) and visuospatial function (MD −1.00, 95% CI −1.98 to −0.02, *n =* 163) (Figure 6). Sensitivity analysis using the final measurement data did not change overall results either (Supplementary Figures S4 and S5).

### 3.7. Sensitivity Analyses and Publication Bias

After excluding seven studies that were in the form of a letter to the editor or conference proceedings, or were rated as high risk in two or more of the RoB areas [22,23,25,26,29,42,43], overall results did not change (data not shown). After exclusion of six studies that included pre-existing conditions such as cerebrovascular disease and diabetes mellitus [24,27,28,29,31,58], overall findings did not change except that the effect of B vitamins on global cognition in studies from Asia and the Middle East became significant (SMD −0.76, 95% CI −0.98 to −0.53, *n =* 320) with an improved statistical heterogeneity (tau^2^ = 0.00 and I^2^ = 0%). Exclusion of trials that recruited participants with vitamin B12 deficiency, elevated homocysteine, or vitamin D deficiency [23,27,32,35,36,38,39,41,44,54,56,58] also resulted in inconsiderable changes, except that the effect of 12 or more months of B vitamin intake on global cognition became nonsignificant (SMD −0.11, 95% CI −0.27 to 0.05, *n =* 2851).

Egger’s test showed no significant funnel asymmetry in the four meta-analyses with 10 or more included studies (B vitamins on global cognition, *p =* 0.215; B vitamins on episodic memory, *p =* 0.877; B vitamins on executive function, *p =* 0.725; and B vitamins on processing speed, *p =* 0.287) (Supplementary Figure S6).

## 4. Discussion

Our analyses revealed that taking B vitamins for over 3 months was beneficial to the global cognition and episodic memory of non-demented people aged 40 years or older. This beneficial effect on global cognition was significant only for people from North America, Europe, and Australia, while it was not for those from Asia and the Middle East. However, sensitivity analyses revealed that, after excluding the trial by Kwok et al. [31], the beneficial effect for those in the latter locations became significant even to a higher degree than that for those in the former locations with a satisfying level of statistical between-trial heterogeneity. One most striking difference in the work by Kwok et al. was that the participants had diabetes mellitus. This condition can be associated with decreased activity of transketolase, resulting in a substantial increase in renal clearance, impaired uptake, and metabolism of thiamine [60,61], which may partly explain why the trial by Kwok et al. demonstrated different results from the others.

The current analysis confirmed that B vitamins were beneficial to the global cognitive function of participants with MCI, with moderate between-trial heterogeneity. For those with normal cognition, we identified only one study reporting an insignificantly beneficial effect of B vitamins, making us unable to draw any meaningful conclusions. Taken together, it is possible to assume that the advantageous effect of B vitamins in previous research might have been driven by participants with MCI. There was only one previous systematic review that compared the effect of B vitamins on cognitive function between participants with and without cognitive impairment [62]. It reported a negligible change in the global and domain-specific measures of cognitive function for people with both existing cognitive impairment (MCI and dementia) and normal cognition. We believe that the discrepant results were due to the inclusion of trials that dealt with people with dementia in this review. People with dementia are unlikely to be responsive to B vitamins due to extensive neuropathological damage. The neurobiological mechanism for MCI individuals underlying the B vitamin effect on cognitive function has yet to be discovered. However, a critical review concerning homocysteine-lowering trials suggested that to observe any beneficial effect of B vitamins within a reasonable time frame (months to several years), study participants should have cognitive decline or at least be on the verge of it [63]. In other words, for cognitively normal individuals, we cannot expect to identify a protective effect of B vitamins against cognitive decline as the decline is not occurring in the first place. For those people with MCI, the protective effect might be more noticeable, which is why our analysis revealed a significant outcome.

There was no evidence on the appropriate duration of vitamin supplementation for cognitive function. B vitamins may exert their effect on the brain directly through their acute influence on hypomethylation, and indirectly through their long-term influence on homocysteine levels [1,64]. Since the effect of B vitamins on cognitive function was larger in the shorter supplementation period (3–11 months) compared to a more extended period (12 months or more), it is tempting to speculate that the beneficial effect of B vitamins may largely rely on the correction of hypomethylation. Through this hypomethylation, a low level of B vitamins is known to be associated with monoamine metabolism disturbances involving serotonin, dopamine, and norepinephrine [65,66], which are ultimately linked to cognitive dysfunction [67]. Additionally, we observed that the intervention effect of B vitamins extending beyond 12 months reduced or even became insignificant after excluding trials targeting participants with a high homocysteine level. This paradoxical decrease of effect size with extending the duration of supplementation may be attributable to the possibility that long-term cumulative effect of habitual dietary patterns and the physiological changes of study participants may mask the beneficial effect of B vitamins. Additionally, a selective increment of homocysteine immunoreactivity involving the hippocampus of B vitamin-deficient rat brains [68] could partly explain why episodic memory alone showed a beneficial effect of B vitamins among other cognitive domains.

In contrast to B vitamins, we did not observe a beneficial effect for antioxidant vitamins on either global or domain-specific cognitive measures when examining changes from baseline. However, sensitivity analyses using final measurements as mean difference estimates let us include additional trials [49,50,52,53] in the meta-analysis, among which two trials reported that there might be a favorable effect on overall cognitive function for an average of 18 years of β-carotene [50] and 5 to 10 years of vitamin C [49] in cognitively normal elderly. It is possible that a relatively short period of supplementation of antioxidant vitamins ranging from 1 to 2 years in the meta-analysis in terms of change from baseline led to a false negative outcome. Previous research also suggested that antioxidant compounds may protect against degenerative neuronal changes by opposing long-term effects of oxidative stress, such as increased production of reactive oxygen species [69].

As for vitamin D, we did not find any significant effect on global cognitive function. Only one study [59] showed a beneficial effect on attention and visuospatial function, as measured by the digit span test and block design, respectively, from the Chinese version of the Wechsler Adult Intelligence Scale—Revised [70]. We believe that, as mentioned above, the protective effect of vitamins is probably more prominent for those with MCI, which is why this study consisting of only MCI subjects showed significant outcomes. However, due to the limited number of included studies in the meta-analysis, we were unable to draw any meaningful conclusions.

We demonstrated our results in terms of SMD, of which significant findings ranged from 0.04 to 0.76 in the effect size. Some might argue that SMD can be difficult to interpret because it reports in units of standard deviation instead of in units of specific measurement scales [71]. However, as a previous article [72] suggested, SMD could provide an efficient way of judging the magnitude of effect following the rule of thumb by Cohen that SMD of 0.2, 0.5, and 0.8 indicates small, medium, and large effects, respectively [73]. Because we incorporated a multitude of neuropsychological test scores to represent domain-specific cognitive measures and it is hard to interpret a clinical significance from a single neuropsychological test, applying this method to explain our findings seems reasonable. It revealed that B vitamin intervention on global cognition for a period of between 3 and 11 months had a large effect with 12 or more months related to a small effect. The same intervention for those from Asia/Middle East excluding trials with diabetes had a large effect, and for those with MCI had a medium effect, while all the other significant findings retained a small effect. As our findings were robust even after excluding trials that targeted participants with vitamin B12 deficiency or elevated homocysteine level, we believe that these effects could be expected from those without an apparent B-vitamin deficiency.

Our review had several limitations. Firstly, although we tried to minimize the between-trial heterogeneity by employing the most frequently used neuropsychological test score for a specific cognitive domain in a single study as the outcome measure, a variety of test scores were used in the meta-analyses in the form of the standardized mean difference. Moreover, several test scores are not highly specific for the designated cognitive domain. For example, trail-making test B can either be included in the executive function or attention domain, and the clock drawing test in the visuospatial function or executive function domains. However, we found that reclassifying these tests into the alternative domain did not result in significant changes in the overall outcome (data not shown). Secondly, the combination and dosage of supplementation varied considerably between clinical trials included in the meta-analysis. This could be a particular problem because of distinct physiological functions by the type of vitamins such as helping the synthesis of serotonin, dopamine, norepinephrine and gamma-aminobutyric acid (GABA) for folate, aiding the synthesis, methylation, and repair of DNA for vitamin B6, and assisting the function and maintenance of nerve cells for vitamin B12 [74]. However, because all three of these B vitamins are particularly important components for methylation when homocysteine is recycled into methionine [64], and folate and vitamin B12 are dependent on each other for their activation [74], it could be worthwhile to treat them collectively and examine their effect in a single model. Thirdly, there was also substantial heterogeneity between trials in terms of baseline age, sex distribution, and sample sizes, which warrants a cautious interpretation of our study results.

## 5. Conclusions

Because of substantial clinical heterogeneity among included trials, we were unable to make any definitive recommendation regarding the duration or dosage of the vitamin intake. Nevertheless, our meta-analyses indicated that B vitamin supplementation for 3 months or longer may be beneficial to the cognitive function of middle-aged or older people even when they do not have an apparent B vitamin-deficiency. It seems plausible that individuals from Asia and the Middle East or those with MCI might be particularly benefitted from this supplementation. Long-term intake of antioxidant vitamins could also be beneficial to cognitive function, while no observable effect was identified for vitamin D.

## Supplementary Materials

The following are available online at https://www.mdpi.com/2072-6643/12/4/1168/s1, Table S1: Search strategies by data sources, Table S2: Characteristics of included studies ordered by study ID for each vitamin group, Table S3: Cognitive domains and their corresponding tests used in each study, Figure S1: Summary of the risk of bias across seven categories for the overall included studies, Figure S2: Effect of B vitamins on global cognition in terms of final measurements by (A) the length of the intervention period, (B) the geographic location of the study, and (C) baseline cognitive function, Figure S3: Effects of B vitamins on (A) episodic memory, (B) executive function, (C) processing speed, (D) attention, and (E) visuospatial function in terms of final measurements, Figure S4: Effect of antioxidant vitamins on (A) episodic memory, (B) executive function, (C) processing speed, and (D) attention in terms of final measurements, Figure S5: Effect of vitamin D on (A) episodic memory, (B) processing speed, (C) attention, and (D) visuospatial function in terms of final measurement, and Figure S6: Funnel plots of meta-analyses with 10 or more included studies.
