# Supplementary material for: Efficacy of Vitamins on Cognitive Function of Non-Demented People: A Systematic Review and Meta-Analysis
Source: Nutrients. 2020 Apr 22;12(4):1168. doi: 10.3390/nu12041168 (PMC7231132; doi:10.3390/nu12041168)
Supplement: Supplementary file 1 [file nutrients-12-01168-s001.pdf]

Table S1. Search strategies by data sources

Data Sources	Search Strategy		
MEDLINE (R) and Epub ahead of print, In-process & other non-indexed citations, Daily and versions (R) (Ovid SP), 1946 to Nov 2019 (Hits retrieved: 1,037)	1. exp *vitamins/	2. exp *dietary supplements/	3. vitamin*.ti,ab.
	4. vitamin A/	5. retinol.ti,ab.	6. “beta-carotene”.ti,ab.
	7. “alpha-carotene”.ti,ab.	8. “gamma-carotene”.ti,ab.	9. "beta-cryptoxanthin".ti,ab.
	10. Vitamin B 12/	11. thiamine.ti,ab.	12. riboflavin.ti,ab.
	13. niacin.ti,ab.	14. nicotinamide.ti,ab.	15. pantothenic.ti,ab.
	16. pyridoxine.ti,ab.	17. pyridoxal.ti,ab.	18. pyridoxamine.ti,ab.
	19. biotin.ti,ab.	20. Folic Acid/	21. “folic acid”.ti,ab.
	22. cobalamin.ti,ab.	23. cyanocobalamin.ti,ab.	24. methylcobalamin.ti,ab.
	25. “l-ascorbic acid”.ti,ab.	26. “ascorbic acid”.ti,ab.	27. Ascorbic Acid/
	28. Vitamin D/	29. cholecalciferol.ti,ab.	30. ergocalciferol.ti,ab.
	31. toxiferol.ti,ab.	32. Vitamin E/	33. tocopherol.ti,ab.
	34. phylloquinone.ti,ab.	35. phytomenadione.ti,ab.	36. phytonadione.ti,ab.
	37. multivitamin*.ti,ab.	38. or/1-37	39. *aging/
	40. aged/	41. “Aged, 80 and over”/	42. middle aged/
	43. age factors/	44. “old* adults”.ti,ab.	45. elderly.ti,ab.
	46. “old* age*”.ti,ab.	47. “middle age*”.ti,ab.	48. seniors.ti,ab.
	49. “senior citizens”.ti,ab.	50. pensioners.ti,ab.	51. “aged sample”.ti,ab.
	52. “aged population”.ti,ab.	53. “mild cognitive impairment”.ti,ab.	54. Mild Cognitive Impairment/
	55. MCI.ti,ab.	56. AAMI.ti,ab.	57. “age-associated memory impairment”.ti,ab.
	58. AACD.ti,ab.	59. “age-associated cognitive decline”.ti,ab.	60. ACMI.ti,ab.
	61. “age-consistent memory impairment”.ti,ab.	62. ARCD.ti,ab.	63. “age-related Cognitive Decline”.ti,ab.
	64. CIND.ti,ab.	65. “cognitive impairment no dementia”.ti,ab.	66. or/39-65
	67. 38 and 66	68. *cognition/	69. *cognition disorders/
	70. memory/	71. memory disorders/	72. (cognit* adj3 (func* or declin* or reduc* or impair* or improve* or deficit* or progress* or perform* or abilit*)).ti,ab.
	73. “mental perform*”.ti,ab.	74. memory.ti,ab.	75. “episodic memory”.ti,ab.
	76. Memory, Episodic/	77. “executive function*”.ti,ab.	78. Executive Function/
	79. Attention/	80. (speed adj2 processing).ti,ab.	81. visuospatial.ti,ab.
	82. language.ti,ab.	83. or/68-82	84. 67 and 83
	85. randomized controlled trial.pt.	86. controlled clinical trial.pt.	87. randomized.ab.

	88. placebo.ab.	89. drug therapy.fs.	90. randomly.ab.
	91. trial.ab.	92. groups.ab.	93. or/85-92
	94. exp Animals/ not humans.sh.	95. 93 not 94	96. 84 and 95
EMBASE, 1974 to Nov 2019 (Hits retrieved: 1,198)	1. 'vitamin'/mj/exp	2. 'diet supplementation'/exp	3. vitamin*:ab,ti
	4. 'vitamin supplementation'/de	5. retinol:ab,ti	6. 'retinoic acid':ab,ti
	7. 'beta carotene'/de	8. 'beta carotene':ab,ti	9. 'alpha carotene'/de
	10. 'alpha-carotene':ab,ti	11. 'gamma carotene'/de	12. 'gamma-carotene':ab,ti
	13. beta-cryptoxanthin:ab,ti	14. 'vitamin B complex'/de OR 'vitamin B group'/de	15. thiamine/de
	16. thiamine:ab,ti	17. riboflavin/de	18. riboflavin:ab,ti
	19. niacin:ab,ti	20. 'nicotinic acid'/de	21. nicotinamide:ab,ti
	22. 'pantothenic acid'/de	23. pantothenic:ab,ti	24. pyridoxamine/de
	25. pyridoxamine:ab,ti	26. biotin/de	27. biotin:ab,ti
	28. 'folic acid'/de	29. 'folic acid':ab,ti	30. cobalamin:ab,ti
	31. cyanocobalamin/de	32. cyanocobalamin:ab,ti	33. methylcobalamin:ab,ti
	34. 'l-ascorbic acid':ab,ti	35. 'ascorbic acid':ab,ti	36. 'ascorbic acid'/de
	37. 'vitamin D'/de	38. 'vitamin D'/de cholecalciferol/de OR calcitriol/de OR 'calcitriol derivative'/de	39. cholecalciferol:ab,ti
	40. ergocalciferol:ab,ti	41. tocopherol:ab,ti	42. 'vitamin K epoxide reductase'/de OR 'vitamin K group'/de
	43. phylloquinone:ab,ti	44. phytonadione:ab,ti	45. phytomenadione:ab,ti
	46. multivitamin*:ab,ti	47. 'vitamin* supple*':ab,ti	48. 'diet* supplement*':ab,ti
	49. #1 OR …… OR #48	50. aging/de	51. aged/de
	52. 'middle aged'/de	53. 'mild cognitive impairment'/de	54. 'mild cognitive impairment':ab,ti
	55. MCI:ab,ti	56. AAMI:ab,ti	57. 'age-associated memory impairment':ab,ti
	58. AACD:ab,ti	59. 'age-associated cognitive decline':ab,ti	60. ACMI:ab,ti
	61. 'age-consistent memory impairment':ab,ti	62. ARCD:ab,ti	63. 'age-related cognitive decline':ab,ti
	64. CIND:ab,ti	65. 'cognitive impairment no dementia':ab,ti	66. 'middle age*':ab,ti
	67. 'old* age*':ab,ti	68. 'old* adults':ab,ti	69. 'senior citizens':ab,ti
	70. seniors:ab,ti	71. pensioners:ab,ti	72. 'aged sample':ab,ti
	73. 'aged population':ab,ti	74. #50 OR …… OR #73	75. 'cognition'/exp
	76. 'cognition disorders'/de	77. 'episodic memory'/de OR memory/de	78. 'memory disorder'/de
	79. dementia/de	80. 'Alzheimer disease'/de	81. dementia*:ab,ti
	82. alzheimer*:ab,ti	83. cognition:ab,ti	84. cognitive:ab,ti

	85. #75 OR …… OR #84	86. #49 AND #74 AND #85	87. 'randomized controlled trial'/de
	88. 'controlled clinical trial'/de	89. placebo:ab	90. (random* NEAR/2 divide*):ab,ti
	91. (random* NEAR/2 allocate*):ab,ti	92. trial:ab	93. 'double-blind*':ab,ti
	94. 'single blind*':ab,ti	95. #87 OR …… OR #94	96. #86 AND #95
	97. [embase]/lim	98. #96 AND #97	
PsycINFO (EBSCOhost), Jan 1806 to Nov 2019 (Hits retrieved: 137)	S1. MJ vitamins +	S2. MJ "Dietary Supplements" +	S3. TI vitamin* OR AB vitamin*
	S4. TI retinol OR AB retinol	S5. TI retinal OR AB retinal	S6. TI "retinoic acid" OR AB "retinoic acid"
	S7. TI "beta-carotene" OR AB "beta-carotene"	S8. TI "alpha-carotene" OR AB "alpha-carotene"	S9. TI "gamma-carotene" OR AB "gamma-carotene"
	S10. TI "beta-cryptoxanthin" OR AB "beta-cryptoxanthin"	S11. MJ "Folic Acid" +	S12. TI "folic acid" OR AB "folic acid"
	S13. TI thiamine OR AB thiamine	S14. TI riboflavin OR AB riboflavin	S15. TI niacin OR AB niacin
	S16. TI nicotinamide OR AB nicotinamide	S17. TI pantothenic OR AB pantothenic	S18. TI pyridoxine OR AB pyridoxine
	S19. TI pyridoxal OR AB pyridoxal	S20. TI pyridoxamine OR AB pyridoxamine	S21. TI biotin OR AB biotin
	S22. TI cobalamin OR AB cobalamin	S23. TI cyanocobalamin OR AB cyanocobalamin	S24. TI methylcobalamin OR AB methylcobalamin
	S25. MJ "ascorbic acid" +	S26. TI "l-ascorbic acid" OR AB "l-ascorbic acid"	S27. TI "ascorbic acid" OR AB "ascorbic acid"
	S28. TI ascorbate OR AB ascorbate	S29. TI cholecalciferol OR AB cholecalciferol	S30. TI ergocalciferol OR AB ergocalciferol
	S31. TI tocopherol OR AB tocopherol	S32. TI phylloquinone OR AB phylloquinone	S33. TI phytomenadione OR AB phytomenadione
	S34. TI phytonadione OR AB phytonadione	S35. TI multivitamin* OR AB multivitamin*	S36. S1 OR …… OR S35
	S37. MJ aging +	S38. MJ "cognitive impairment" +	S39. TI "cognit* impair*" OR AB "cognit* impair*"
	S40. TI MCI OR AB MCI	S41. TI "mild cognitive impairment" OR AB "mild cognitive impairment"	S42. TI AAMI OR AB AAMI
	S43. TI "age-associated memory impairment" OR AB "age-associated memory impairment"	S44. TI AACD OR AB AACD	S45. TI "age-associated cognitive decline" OR AB "age-associated cognitive decline"
	S46. TI ACMI OR AB ACMI	S47. TI "age-consistent memory impairment" OR AB "age-consistent memory impairment"	S48. TI ARCD OR AB ARCD
	S49. TI "age-related cognitive decline" OR AB "age-related cognitive	S50. TI CIND OR AB CIND	S51. TI "cognitive impairment no dementia" OR AB

	decline"		"cognitive impairment no dementia"
	S52. TI “old* age*” OR AB “old* age*”	S53. TI elderly OR AB elderly	S54. TI “middle age*” OR AB “middle age*”
	S55. TI “old* adults” OR AB “old* adults”	S56. TI seniors OR AB seniors	S57. TI “senior citizens” OR AB “senior citizens”
	S58. TI pensioners OR AB pensioners	S59. MJ cognition +	S60. MJ dementia +
	S61. S37 OR …… OR S60	S62. MA "Randomized Controlled Trials"	S63. AB randomly
	S64. AB placebo	S65. AB groups	S66. AB RCT
	S67. TX "double blind*”	S68. TX "single blind*”	S69. TX "controlled clinical trial"
	S70. TI randomised	S71. TI randomized	S72. S62 OR …… S71
	S73. S36 AND S61 AND S72		
CINAHL (EBSCOhost) , 1961 to Nov 2019 (Hits retrieved: 48)	S1. MM "Vitamins+"	S2. TI vitamin* OR AB vitamin*	S3. MH "Vitamin A"
	S4. (MH "Vitamin B12") OR (MH "Vitamin B Complex") OR (MH "Thiamine") OR (MH "Riboflavin") OR (MH "Pyridoxine")	S5. MH "Folic Acid"	S6. MH "Ascorbic Acid"
	S7. (MH "Vitamin D") OR (MH "Cholecalciferol") OR (MH "Ergocalciferols") OR (MH "Calcitriol")	S8. (MH "Vitamin E") OR (MH "Pantothenic Acid") OR (MH "tocopherol")	S9. (MH "Vitamin K") OR (MH "Osteocalcin")
	S10. TI "beta-carotene" OR AB "beta-carotene"	S11. TI "alpha-carotene" OR AB "alpha-carotene"	S12. TI thiamine OR AB thiamine
	S13. TI riboflavin OR AB riboflavin	S14. TI niacin OR AB niacin	S15. TI pantothenic OR AB pantothenic
	S16. TI nicotinamide OR AB nicotinamide	S17. TI pyridoxine OR AB pyridoxine	S18. TI pyridoxal OR AB pyridoxal
	S19. TI biotin OR AB biotin	S20. S1 OR …… OR S19	S21. MH "Aging"
	S22. (MH "Aged") OR (MH "Aged, 80 and Over")	S23. MH "Middle Age"	S24. TI MCI OR AB MCI
	S25. TI AAMI OR AB AAMI	S26. TI ACMI OR AB ACMI	S27. TI ARCD OR AB ARCD
	S28. TI CIND OR AB CIND	S29. TI AACD OR AB AACD	S30. TI "Mild Cognitive Impairment" OR AB "Mild Cognitive Impairment"
	S31. TI "age-associated memory impairment" OR AB "age-associated memory impairment"	S32. TI "age-consistent memory impairment" OR AB "age-consistent memory impairment"	S33. TI "age-related cognitive decline" OR AB "age-related cognitive decline"
	S34. TI "cognitive impairment no dementia"	S35. TI "age-associated cognitive decline" OR AB	S36. TI elderly OR AB elderly

	OR AB "cognitive impairment no dementia"	"age-associated cognitive decline"	
	S37. TI "old* adults" OR AB "old* adults"	S38. TI "old* age*" OR AB "old* age*"	S39. TI pensioners OR AB pensioners
	S40. TI seniors OR AB seniors	S41. TI "senior citizen*" OR AB "senior citizen*"	S42. TI "age* sample" OR AB "age* sample"
	S43. TI "age* population" OR AB "age* population"	S44. S21 OR …… OR S43	S45. (MH "Cognition") OR (MH "Cognition Disorders") OR (MH "Delirium, Dementia, Amnestic, Cognitive Disorders")
	S46. TI cognition OR AB cognition	S47. TI memory OR AB memory	S48. (MH "Memory") OR (MH "Memory Disorders") OR (MH "Memory, Short Term")
	S49. TI "executive function" OR AB "executive function"	S50. TI "cognitive* declin*" OR AB "cognitive* declin*"	S51. TI "cognitive* improv*" OR AB "cognitive* improv*"
	S52. TI "cognitive deficit*" OR AB "cognitive deficit*"	S53. TI "mental perform*" OR AB "mental perform*"	S54. TI dementia OR AB dementia
	S55. TI alzheimer* OR AB alzheimer*	S56. MH "Dementia+"	S57. S45 OR …… OR S56
	S58. MH "Randomized Controlled Trials"	S59. AB randomly	S60. AB placebo
	S61. AB groups	S62. AB RCT	S63. TI "double blind*" OR AB "double blind*"
	S64. TI "single blind*" OR AB "single blind*"	S65. TI "controlled clinical trial" OR AB "controlled clinical trial"	S66. TI randomised
	S67. TI randomized	S68. S58 OR …… OR S67	S69. S20 AND S44 AND S57 AND S68
Cochrane Central Register of Controlled Trials (CENTRAL), Nov 2019 (Hits retrieved: 696)	#1. MeSH descriptor: [Vitamins] explode all trees	#2. (vitamin*):ti,ab,kw	#3. MeSH descriptor: [Vitamin A] this term only
	#4. (retinol):ti,ab,kw	#5. (beta-carotene):ti,ab,kw	#6. (alpha-carotene):ti,ab,kw
	#7. (gamma-carotene):ti,ab,kw	#8. (beta-cryptoxanthin):ti,ab,kw	#9. MeSH descriptor: [Vitamin B 12] this term only
	#10. (thiamine):ti,ab,kw	#11. (riboflavin):ti,ab,kw	#12. (niacin):ti,ab,kw
	#13. (nicotinamide):ti,ab,kw	#14. (pantothenic):ti,ab,kw	#15. (pyridoxine):ti,ab,kw
	#16. (pyridoxal):ti,ab,kw	#17. (pyridoxamine):ti,ab,kw	#18. (biotin):ti,ab,kw
	#19. MeSH descriptor: [Folic Acid] this term only	#20. (cobalamin):ti,ab,kw	#21. (cyanocobalamin):ti,ab,kw
	#22. (methylcobalamin):ti,ab,k	#23. (l-ascorbic acid):ti,ab,kw	#24. (ascorbic acid):ti,ab,kw

w		
#25. MeSH descriptor: [Ascorbic Acid] this term only	#26. MeSH descriptor: [Vitamin D] this term only	#27. (cholecalciferol):ti,ab,kw
#28. (ergocalciferol):ti,ab,kw	#29. MeSH descriptor: [Vitamin E] this term only	#30. (tocopherol):ti,ab,kw
#31. (phylloquinone):ti,ab,kw	#32. (phytomenadione):ti,ab,kw	#33. (phytonadione):ti,ab,kw
#34. (multivitamin*):ti,ab,kw	#35. #1 OR …… OR #34	#36. MeSH descriptor: [Aged] in all MeSH products
#37. MeSH descriptor: [Aged, 80 and over] this term only	#38. MeSH descriptor: [Middle Aged] this term only	#39. MeSH descriptor: [Age Factors] this term only
#40. (old* adults):ti,ab,kw	#41. (elderly):ti,ab,kw	#42. (old* age*):ti,ab,kw
#43. (middle age*):ti,ab,kw	#44. (seniors):ti,ab,kw	#45. (senior citizens):ti,ab,kw
#46. (pensioners):ti,ab,kw	#47. (aged sample):ti,ab,kw	#48. (aged population):ti,ab,kw
#49. (mild cognitive impairment):ti,ab,kw	#50. MeSH descriptor: [Cognitive Dysfunction] this term only	#51. (MCI):ti,ab,kw
#52. (AAMI):ti,ab,kw	#53. (age-associated memory impairment):ti,ab,kw	#54. (AACD):ti,ab,kw
#55. (age-associated cognitive decline):ti,ab,kw	#56. (ACMI):ti,ab,kw	#57. (age-consistent memory impairment):ti,ab,kw
#58. (ARCD):ti,ab,kw	#59. (age-related Cognitive Decline):ti,ab,kw	#60. (CIND):ti,ab,kw
#61. (cognitive impairment no dementia):ti,ab,kw	#62. #36 OR …… OR #61	#63. (cognitive impairment no dementia):ti,ab,kw
#64. MeSH descriptor: [Cognition Disorders] this term only	#65. MeSH descriptor: [Memory] this term only	#66. MeSH descriptor: [Memory Disorders] this term only
#67. (cognit* NEAR/3 (func* OR declin* OR reduc* OR impair* OR improve* OR deficit* OR progress* OR perform* OR abilit*)):ti,ab,kw	#68. (mental perform*):ti,ab,kw	#69. (memory):ti,ab,kw
#70. (memory):ti,ab,kw	#71. MeSH descriptor: [Memory, Episodic] this term only	#72. (executive function*):ti,ab,kw
#73. (executive function*):ti,ab,kw	#74. MeSH descriptor: [Attention] this term only	#75. (speed NEAR/2 processing):ti,ab,kw
#76. (visuospatial):ti,ab,kw	#77. (language):ti,ab,kw	#78. #63 OR …… OR #77
#79. (randomized	#80. (controlled clinical	#81. (randomized):ab

	controlled trial):pt	trial):pt	
	#82. (placebo):ab	#83. (randomly):ab	#84. (trial):ab
	#85. (groups):ab	#86. #79 OR …… OR #85	#87. #35 AND #62 AND #78 AND #86
International Clinical Trials Registry Platform Search Portal* Nov 2019 (Hits retrieved: 122)	**A:** in the Condition box: cognition OR "mild cognitive impairment" OR elderly OR "aged subjects" OR "older adults" OR "middle aged"<br>**B:** in the Intervention box: vitamin* OR "diet* suppl*" OR retinol OR carotene OR thiamine OR riboflavin OR niacin OR nicotinamide OR pantothenic OR pyridoxine OR biotin OR "folic acid" OR cobalamin OR "ascorbic acid" OR cholecalciferol OR ergocalciferol OR tocopherol OR multivitamin<br>**A and B**<br>Recruitment status is: ALL		
Open Grey database Nov 2019 (Hits retrieved: 55)	abstract: (dementia OR cognitive OR cognition OR "mild cognitive impairment" OR elderly OR "aged subjects" OR "older adults" OR "middle aged") AND (vitamin* OR "diet* suppl*" OR retinol OR carotene OR thiamine OR riboflavin OR niacin OR nicotinamide OR pantothenic OR pyridoxine OR biotin OR "folic acid" OR cobalamin OR "ascorbic acid" OR cholecalciferol OR ergocalciferol OR tocopherol OR multivitamin)		

* Data providers include: Australian New Zealand Clinical Trials Registry; Chinese Clinical Trial Registry; ClinicalTrials.gov; EU Clinical Trials Register (EU-CTR); International Standard Randomised Controlled Trial Number (ISRCTN); The Netherlands National Trial Register; Brazilian Clinical Trials Registry (ReBec); Clinical Trials Registry – India; Clinical Research Information Service - Republic of Korea; Cuban Public Registry of Clinical Trials; German Clinical Trials Register; Iranian Registry of Clinical Trials; Japan Primary Registries Network; Pan African Clinical Trial Registry; Sri Lanka Clinical Trials Registry; Thai Clinical Trials Registry (TCTR); and Peruvian Clinical Trials Registry (REPEC).

Table S2. Characteristics of included studies ordered by study ID for each vitamin group

B vitamins

Andreeva, 2011

Methods	SU.FOL.OM3 randomized trial; multicenter, randomized, double-blind, placebo-controlled, secondary prevention trial
Participants	Location: France<br>Setting of recruitment and treatment: conducted between 2003 and 2009; were recruited via a network of 417 cardiologists, neurologists, and other physicians throughout France.<br>Sample size<br>● Number randomized: 446 in intervention, 425 in placebo<br>● Number completed (95%)<br>Participant baseline characteristics<br>● Age in years, mean (SD): intervention, 61.4 (8.7); placebo, 60.9 (8.9)<br>● Female sex: intervention, 93 (20.9); placebo, 96 (22.6)<br>● Cognitive function (CN, MCI) – Isaacs Set Test, mean (SD): intervention, 35.7 (7.9); placebo, 36.1 (7.1)<br>**Inclusion criteria**<br>Men and women aged 45–80 y w**ith a recent myocardial infarction (MI), unstable angina, or ischemic stroke** were eligible for participation.<br>**Exclusion criteria**<br>Patients that are incapable of understanding the study protocol or who refuse to sign the informed consent; patients with a pathology that might interfere with homocysteine or omega-3 fatty acid metabolism, in particular those that use methotrexate for the treatment of a cancer or rheumatoid arthritis; chronic renal failure (plasma level of creatinine > 200 μmol/L or creatinine clearance < 40 ml/min); patients with a non-cardiovascular pathology with a suspected survival time less than the 5 years period of the study (solid cancer, evolved dementia, leukemia, etc.); patients taking treatment with B vitamins or omega-3 fatty acids.
Interventions	**Intervention**: B vitamins: 5-methyltetrahydrofolate (5-methyl-THF, 0.56 mg), vitamin B-6 (3 mg), and vitamin B-12 (0.02 mg)<br>**Placebo:** placebo capsules were made of gelatin manufactured by Catalent Pharma Solutions<br>The supplements were given as 2 capsules to be taken once daily
Outcomes	The participants were given sufficient supplements for 1 y and were reexamined at annual follow-up visits<br>At 4 year<br>**Global**: F-TICS (French version of the modified Telephone Interview for Cognitive Status)<br>**Memor**y: Repetition (%) of F-TICS
Notes	Funding: supported by the French Ministry of Research (grant R02010JJ), the Ministry of Health, Sodexo, Candia, Unilever, Danone, Roche Laboratories, Merck EPROVA AG, and Pierre Fabre Laboratories.

ROB

Bias	Judge	Support for judgement
Random sequence generation (selection bias)	Low	Quote: We used computerized block randomization (block size = 8) with stratification by sex, age (45–54, 55–64, and 65–80 y), prior CVD, and city of residence.

Allocation concealment (selection bias)	Low	Quote: The statistics team at the trial's coordinating center randomized participants in a 2-by-2 factorial design to 1 of 4 daily treatment groups:
Blinding of participants and personnel (performance bias)	Unclear	No information
Blinding of outcome assessment (detection bias)	Unclear	No information
Incomplete outcome data (attrition bias)	Unclear	Comment: Exact numerical data of those completed trial for each intervention arm were not given.
Selective reporting (reporting bias)	Low	Quote: This trial is registered at controlled-trials.com as ISRCTN41926726.
Other bias	Low	No other sources of bias identified

Cheng, 2016

Methods	Placebo-controlled trial
Participants	Location: China<br>Setting of recruitment and treatment: volunteers from five communities and two nursing homes in Tianjin city, China,<br>Sample size<br>● Number randomized: 57 in intervention, 47 in placebo<br>● Number completed: 42 in intervention, 41 in placebo<br>Participant baseline characteristics<br>● Age in years, mean (SD): intervention, 74.3 (9.6); placebo, 72.5 (7.0)<br>● Female sex: intervention, 30 (52.6%); placebo, 24 (51.1%)<br>● Cognitive function (CN, MCI) – Basic Cognitive Aptitude Tests, mean (SD): intervention, 49.9 (16.3); placebo, 51.9 (19.0)<br>**Inclusion criteria**<br>aged 55–94 years old; All subjects were Chinese Han people.; The eligible participants with HHcy (serum tHcy concentration ≥ 16 μmol/l) were screened<br>**Exclusion criteria**<br>with serious renal or hepatic disease, diabetics, cancer, hyper- or hypothyroidism and diseases of the nervous system, and acoustic or visual disorders were excluded
Interventions	Intervention: a 14-week treatment with daily oral doses of a combination of 800 μg **folate**, 10 mg vitamin B6, and 25 μg vitamin B12.<br>Placebo: The control group was given a placebo capsule daily.
Outcomes	At 3.5 months<br>**memory**: recognition of two-word nouns (RTN),<br>**visuospatial**: Chinese character rotation (CCR),
Notes	Funding: The study was supported by the State Key Program of National Natural Science Foundation of Tianjin (No. 14JCZDJC36100), Danone Institute China Diet Nutrition Research and Communication Grant (No. DIC2006-08) and Tianjin Application Basic and Front Technology Research Project Grant (No.09JCYBJC12900).

ROB

Bias	Judge	Support for judgement
Random sequence generation (selection bias)	High	Quote: allocated on matching principle

Allocation concealment (selection bias)	High	Quote: assigned to two groups according to their age, education level, and initial BCAT scores.<br>Comment: no information about concealment
Blinding of participants and personnel (performance bias)	Unclear	Comment: no information
Blinding of outcome assessment (detection bias)	Unclear	Comment: no information
Incomplete outcome data (attrition bias)	High	Comment: discrepant drop-out rates between two arms.
Selective reporting (reporting bias)	Unclear	Comment: no information
Other bias	Low	No other sources of bias identified.

Dangour 2015

Methods	double-blind, randomized, placebo-controlled trial, 12 months duration
Participants	Location: 7 general practices in South East England, United Kingdom<br>**Setting of recruitment and treatment**: enrolled at 7 general practices in South East England that were members of the Medical Research Council General Practice Research Framework or the National Institute of Health Research Primary Care Research Network.<br>Sample size<br>● Number randomized: 99 in intervention, 102 in placebo<br>● Number completed: 91 in intervention, 92 in placebo<br>Participant baseline characteristics<br>● Age in years, mean (SD): intervention, 79.9 (3.5); placebo, 80.1 (3.7)<br>● Female sex: intervention, 53/99 (53.5%); placebo, 54/102 (52.9%)<br>● Cognitive function (CN, MCI) – MMSE, mean (IQR): intervention, 29 (28-29); placebo, 29 (28-29)<br>**Inclusion criteria**<br>aged ≥75 y; Mini-Mental State Examination score ≥ 24 (maximum score: 30); Individuals with moderate vitamin B-12 deficiency who did not have anemia (serum vitamin B-12 concentrations ≥107 and <210 pmol/L [Beckman Coulter assay] and hemoglobin concentrations ≥110 g/L for women and ≥120 g/L for men) were eligible to join the trial.<br>**Exclusion criteria**<br>Exclusion of individuals with diabetes, dementia, or epilepsy. exclude individuals with alcohol addiction, pacemakers, or other implanted metallic devices (for whom central neurophysiologic testing was contraindicated), residents of nursing homes, and anyone with a previous diagnosis of pernicious anemia; Individuals who reported current consumption of vitamin B-12 supplements or who had received a vitamin B-12 injection in the previous 6 mo were excluded; Individuals with very-low vitamin B-12 concentrations (<107 pmol/L, which is a cutoff typically used for deficiency; Beckman Coulter assay, Beckman Coulter Inc.) or who were shown to have anemia (hemoglobin concentration <110 g/L for women and <120 g/L for men) were excluded.
Interventions	Intervention: 1 mg **crystalline vitamin B-12** for 12 months<br>Placebo: matching pill
Outcomes	At 12 months<br>**Memory:** California Verbal Learning Test, Total words correct in first 3 trials, n;

	Words recalled at delayed recall, n<br>**Executive**: Verbal fluency, n animals named<br>**Processing speed**: Symbol letter modality, n correct in 90 sec; Reaction time, s, simple; choice
Notes	**Funding:** Supported by the Food Standards Agency (N05072) and the Department of Health. National Health Service Research and Development and King's College Hospital Trust Research and Development provided service support costs. DSM donated the vitamin B-12 form used to manufacture study tablets. This is an open access article distributed under the CC-BY license (http://creativecommons.org/ licenses/by/3.0/). The funders had no role in the implementation, data collection, management, analysis, or interpretation of the study or in the preparation, review, and approval of the manuscript.<br>**Compliance**: Adherence to allocated treatment was measured by counting the number of tablets returned at the end of the study. At baseline and 12 mo after random assignment, blood samples were collected to measure serum concentrations of vitamin B-12 (microbiologic assay; CV range: 5–7%), holotranscobalamin (Axis- Shield radioimmunoassay; CV range: 5–8%; Axis-Shield plc), total homocysteine (Abbott IMx analyzer; CV range: 2–3%; Abbott Laboratories), and folate (chloramphenicol-resistant microbiologic assay; CV range: 5–8%).

ROB

Bias	Judge	Support
Random sequence generation (selection bias)	Low	Quote: Allocation codes were obtained from a central computerized randomization service. Allocation to treatment was balanced by age (75–79 and ≥80 y) and sex.
Allocation concealment (selection bias)	Unclear	Quote: All study personnel were blinded to the treatment allocation.
Blinding of participants and personnel (performance bias)	Low	Quote: Identical in size, shape, color, smell, and taste for both the intervention and placebo and packaged into identical pots each of which contained 70 tablets.
Blinding of outcome assessment (detection bias)	Unclear	Quote: At baseline and after 12 mo, the study manager (KW) administered a range of cognitive function tests.
Incomplete outcome data (attrition bias)	Low	Quote: 262 potential participants were shown to be ineligible, largely because their serum vitamin B-12 concentrations were out of range.<br>Of 209 participants who were randomly allocated to the study between January 2009 and May 2010, 8 subjects were randomly assigned in error (as a result of protocol deviations) and provided no additional data. Six participants withdrew from the study (2 subjects from the vitamin B-12 arm and 4 subjects from the placebo arm), and one participant died.<br>There was no difference between trial arms in the number of tablets returned at the end of the study (mean: 37 tablets in the vitamin B-12 arm and 39 tablets in the placebo arm).
Selective reporting (reporting bias)	Low	Quote: Details of the trial protocol have been published (www.isrctn.com; ISRCTN54195799)

Other bias	Unclear	Quote: In comparison, compared with baseline amounts, allocation to the placebo was associated with small changes (0–5%) in biochemical variables (Table 4).

Deijen 1992

Methods	Quasi-randomized, placebo-controlled trial
Participants	Location: Netherlands<br>Setting of recruitment and treatment: Subjects were recruited by means of advertisements in local papers.; placebo subjects were matched for age, plasma pyridoxal-5'-phosphate (PLP) concentration and intelligence score.<br>Sample size<br>● Number randomized: 41 in intervention, 41 in placebo<br>● Number completed: 38 in intervention, 38 in placebo<br>Participant baseline characteristics<br>● Age in years, mean (SD): intervention, 73 (3); placebo, 73 (3)<br>● Female sex: 0%<br>● Cognitive function (CN, MCI) – IQ, mean (SD): intervention, 109 (11); placebo, 111 (10)<br>● At the start of the study 13 subjects (9 placebo, 4 vitamin B-6) of the group of 76 participants (17%) were defined as marginally deficient on the basis of plasma PLP (PLP < 20 nmol/1) and three subjects (4%) on the basis of a-EAST (> 1.98), all three belonging to the placebo group.<br>**Inclusion criteria**<br>self-supporting healthy men, aged between 70-79 years.; apparently healthy/age between 70 and 79 year/restricted alcohol use (a maximum of four glasses a day)/consent to inform the physician/intelligence score above 80.<br>**Exclusion criteria**<br>use of drugs affecting the vitamin B-6 metabolism, i.e. levodopa, (di) hydralazine, amitriptyline, isoniazide, amiodarone, penicillamin and MAO inhibitors/use of drugs affecting the immune reactivity i,e, corticosteroids and cytostatics/use of vitamin B-6 supplements within 3 months before the study period/ auto-immune diseases, like rheumatoid arthritis/use of tong-acting hypnotics, i.e. flunitrazepam, flurazepam, quazepam/use of antidepressants within a month before study period/addicted to drugs or alcohol/abnormal clinical chemical-haematological profile/sensory or motor defect which may affect performance.
Interventions	Intervention: **vitamin B-6 supplementation** (20 mg pyridoxine HCL daily for 3 months)<br>Placebo: placebo, administered in identical capsules.
Outcomes	At 3 months<br>a condensed version of the Groninger Intelligence Test<br>**Episodic memory**: Long-term memory storage. The scores on trial 3 of Associate Learning (AL3) and on Associate Recognition (AR) were combined in a "**Forget score**", computed as: Forget = AL3-AR. The value of this difference indicates the amount of information, learned before, that is not stored in LTM. Thus, the smaller this forget value the more information is stored in LTM.

Notes	Funding: N/A<br>Compliance: On two random occasions during the study period urine samples were collected. From these samples 4-pyridoxine acid excretion was measured. Apart from inspection of these samples, the compliance could be judged by considering the increase in vitamin B-6 status of each subject in the treatment group.

ROB

Bias	Judge	Support for judgement
Random sequence generation (selection bias)	High	Quote: Pairs of subjects were formed, each pair matched on age, vitamin B-6 status and IQ scores. Subsequently, each one of the two was randomly allocated to the placebo group or the B-6 group, according to a double-blind procedure.<br>Comment: subjects were not randomly distributed
Allocation concealment (selection bias)	Unclear	Quote: Pairs of subjects were formed, each pair matched on age, vitamin B-6 status and IQ scores. Subsequently, each one of the two was randomly allocated to the placebo group or the B-6 group, according to a double-blind procedure.
Blinding of participants and personnel (performance bias)	Low	Quote: Pairs of subjects were formed, each pair matched on age, vitamin B-6 status and IQ scores. Subsequently, each one of the two was randomly allocated to the placebo group or the B-6 group, according to a double-blind procedure.<br>Treatment consisted of vitamin B-6 or placebo. Afterwards subjects were informed if they had been given placebo or vitamin B-6.<br>placebo, administered in identical capsules.<br>The capsules were given to the subjects on two occasions in the study period, in envelopes containing blister cards, for a period of 8 weeks.
Blinding of outcome assessment (detection bias)	Unclear	No information
Incomplete outcome data (attrition bias)	High	**Quote:** Three subjects dropped out because of illness. Because of the matched pairs procedure, which is described in the next section, this lead to the exclusion of three other, matched subjects.<br>**Comment:** this deliberate exclusion of matching pairs could lead to attrition bias.
Selective reporting (reporting bias)	High	Comment: not all the cognitive measures were reported
Other bias	Low	No other sources of bias identified

de Jager 2012

Methods	A randomized trial (VITACOG), double-blind, single-centre study, 24 months duration
Participants	Location: United Kingdom<br>Setting of recruitment and treatment: Respondents to recruitment advertising from the Oxford area.; Participants in the Oxford area were

	**recruited between April 2004 and November 2006** through advertisements in the local newspaper or radio seeking elderly people with concerns about their memory.<br>Sample size<br>• Number randomized: 138 in intervention, 133 in placebo<br>• Number completed: 110 in intervention, 113 in placebo<br>**Sample size for high homocysteine group**<br>• Number randomized: 24 in intervention, 25 in placebo<br>• Number completed: 24 in intervention, 25 in placebo<br>Participant baseline characteristics<br>• Age in years, mean (SD): intervention, 76.8 (5.1); placebo, 76.7 (4.8)<br>• Female sex: intervention, 70/110 (63.6%); placebo, 73/113 (64.6%)<br>• Cognitive function (MCI) – TICS-M, mean (SD): intervention, 24.9 (2.8); placebo, 24.9 (2.8)<br>**Inclusion criteria**<br>Age ≥70 years; study partner available as informant, and diagnosis of amnestic or non-amnestic MCI according to Petersen's criteria. The diagnosis included a subjective concern about memory that did not interfere with activities of daily living, assessed with 4 questions on subjective memory complaints from the Cambridge Mental Disorders of the Elderly Examination (CAMDEX) and 5 questions relating to activities of daily living based on the Cambridge Behavioural Inventory, an objective memory problem assessed with the 'Telephone interview of cognitive status, modified' (TICS-M), a test without a ceiling effect, and category fluency based on previously defined cut-off scores for MCI. Thus, eligible subjects had a score of 17 – 29 out of a maximum of 39 on TICS-M. For borderline cases, if TICS-M was >29 but category fluency <19 or TICS-M word recall ≤10/20, then subjects were eligible. Alternatively, if TICS-M was <17 but category fluency was ≥19 or word recall was ≥10/20, then subjects were also eligible. Other measures to confirm the MCI diagnosis collected at the first visit were a Mini-mental state examination (MMSE) score of > 24/30 and no evidence of dementia.<br>**Exclusion criteria**<br>a diagnosis of dementia or being treated with anti-dementia drugs; active cancer; major stroke within past 3 months; treatment with methotrexate, anti-cancer or anti-epileptic drugs, or taking folic acid >300 mg/d, pyridoxine >3 mg/d or vitamin B12 >1.5 mg/d by mouth or any dose by injection. Those taking B vitamins below these doses were allowed to continue during the trial.
Interventions	Intervention: 0.8mg folic acid, 0.5mg vitamin B12 and 20mg vitamin B6 for 24 months<br>Placebo: vitamin-free tablets of similar appearance
Outcomes	At 24 months (for TICS-M, at 15, 27 months) for **high/low homocysteine group**<br>**Global**: MMSE;<br>**Memory**: Hopkins Verbal Learning Test-revised with delayed recall<br>**Executive:** categorical fluency;<br>**Visuospatial**: CLOX2
Notes	**Funding**: The sponsor (University of Oxford), the funders of the study and the company providing the tablets had no role in the study design, data collection, data analysis, data interpretation or writing of the report.

	**Homocysteine:** Cognitive test scores based on both high and low baseline level of homocysteine included in the study. We extracted data from participants with high baseline level of homocysteine. This might result in the exaggeration of treatment effect but replacing them with data from low baseline level of homocysteine did not change overall results.

ROB

Bias	Judge	Support
Random sequence generation (selection bias)	Low	Quote: Centralized telephone randomization was used with full allocation concealment and minimization for age, gender, TICS-M score and MRI consent.
Allocation concealment (selection bias)	Low	Comment: with full allocation concealment
Blinding of participants and personnel (performance bias)	Low	Quote: Participants, study partners and those assessing outcomes were blind to the assignment of interventions.
Blinding of outcome assessment (detection bias)	Low	Quote: Participants, study partners and those assessing outcomes were blind to the assignment of interventions.
Incomplete outcome data (attrition bias)	Low	Quote: There was no difference between baseline demographics for the 43 participants who failed to complete the trial compared with the 223 participants who completed the trial
Selective reporting (reporting bias)	Low	Quote: The trial was registered under ISRCTN94410159.
Other bias	Low	Comment: No other sources of bias identified

Durga, 2007

Methods	Randomised, double blind, placebo controlled study, 36 months duration
Participants	Location: from the Gelderland region in the Netherlands<br>Setting of recruitment and treatment: From the Folic Acid and Carotid Intimamedia Thickness (FACIT) study. We used municipal and blood-bank registries to recruit participants.<br>Sample size<br>• Number randomized: 406 in intervention, 413 in placebo<br>• Number completed: 405 in intervention, 413 in placebo<br>Participant baseline characteristics<br>• Age in years, mean (SD): intervention, 60(5); placebo, 60 (6)<br>• Female sex: intervention, 111/405 (27%); placebo, 121/413 (29%)<br>• Cognitive function (CN, MCI) – MMSE, mean (IQR): intervention, 29 (28-30); placebo, 29 (28-30)<br>**Inclusion criteria**<br>Aged 50-70;<br>**Exclusion criteria**<br>Concentrations of plasma total homocysteine of less than 13 umol/L (73% of those screened). We excluded participants with raised homocysteine concentrations (>26 μmol/L) that were possibly due to factors other than suboptimal folate concentrations, including: serum vitamin B12 concentration of less than 200 pmol/L (10% of those screened); self-reported

	medical diagnosis of renal or thyroid disease; or self-reported use of medications that influence folate metabolism. Additionally, we excluded participants with self-reported intestinal disease and participants who reportedly used B-vitamin supplements or drugs that could affect atherosclerotic progression; dementia with MMSE < 24
Interventions	**Intervention**: 800 μg daily oral **folic acid** for 36 months<br>**Placebo**: No information
Outcomes	At 36 months<br>**Global:** average of domains, composite Z score<br>**Memory**: composite Z score<br>**Executive:** verbal fluency Z score<br>**Processing speed:** letter digit substitution test Z score; sensorimotor speed Z score; complex speed Z score, information processing speed Z score
Notes	**Funding:** Jane Durga currently works at Nestle Research Center in Lausanne, Switzerland and Petra Verhoef currently works at the Unilever Food and Health Research Institute in Vlaardingen, the Netherlands. The work at both food companies entails examining the health benefits of a variety of food ingredients, including folic acid. However, the study reported in the current manuscript was completed and submitted to The Lancet before the authors joined the companies, when they were still employed by Wageningen University and Wageningen Centre for Food Sciences. All authors declare that they have no conflict of interest.<br>The sponsors had no role in the design or implementation of the study, data collection, data management, data analysis, data interpretation, or in the preparation, review, or approval of the manuscript.<br>**Compliance:** More than 80% self-reported compliance during a 6-week placebo run-in period was required.<br>Compliance was judged by capsule-return counts and a diary that registered missed capsules. Diaries and capsules were returned by participants every 12 weeks.<br>Apart from these participants, the compliance of the participants was high, with 99% of the capsules reportedly consumed.<br>**Adverse outcomes:** Five participants allocated folic acid treatment reported adverse events: forgetfulness, sun allergies, weight gain, tinnitus, and dark urine. Adverse e! ects reported in the placebo group (n=7) were muscle aches, headaches, weight gain, queasiness, bitter taste in mouth, and skin irritations.

ROB

Bias	Judge	Support
Random sequence generation (selection bias)	Low	Quote: Patients were allocated treatment or placebo with permuted blocks of sizes four and six, which varied randomly.
Allocation concealment (selection bias)	Low	Quote: Specialized staff who were not involved in the study allocated and labelled the capsule boxes with participants' unique sequence number.
Blinding of participants and personnel (performance bias)	Low	Quote: Participants in the same household received the same treatment.<br>The folic acid and placebo capsules, produced by Swiss-Caps Benelux (Heerhugowaard, Netherlands), were indistinguishable in appearance.

		At the end of the study, the proportion of participants who thought they had received folic acid or placebo did not differ between the two groups (p=0·64).
Blinding of outcome assessment (detection bias)	Low	Quote: All staff, including all authors, were unaware of group assignment until completion of the trial and after data analyses.
Incomplete outcome data (attrition bias)	Low	Quote: The proportion of participants lost to follow-up or who stopped treatment early did not differ between the groups (p=0·25).
Selective reporting (reporting bias)	Low	Quote: This trial is registered with clinicaltrials.gov with trial number NCT00110604
Other bias	Low	Comment: No other sources of bias identified.

Eussen, 2006

Methods	a double-blind, placebo-controlled trial,
Participants	Location: Netherlands<br>Setting of recruitment and treatment: recruited from different parts of the Netherlands via mailed health questionnaires; Screening for vitamin B-12 status was carried out between April 2003 and March 2004.<br>Sample size<br>● Number randomized: 64 in intervention1, 66 in intervention2, 65 in placebo<br>● Number completed: 54 in intervention1, 51 in intervention2, 57 in placebo<br>Participant baseline characteristics<br>● Age in years, mean (SD): intervention1, 82 (5); intervention2, 83 (6); placebo, 82 (5)<br>● Female sex: intervention1, 49 (77%); intervention2, 49 (74%); placebo, 51 (78%)<br>● Cognitive function (CN, MCI) – MMSE, mean (SD): intervention1, 26.7 (3.1); intervention2, 26.7 (3.0); placebo, 26.8 (2.9)<br>**Inclusion criteria**<br>persons aged 70 or more y with mild vitamin B-12 deficiency.; Free-living older persons and older persons living in care facility homes; Medication interfering with vitamin B-12 absorption was permitted if it had been provided 3mo or more before the screening of vitamin B-12 status and was intended to be continued for the duration of the trial.; Individuals who fulfilled the criteria for mild vitamin B-12 deficiency were eligible<br>**Exclusion criteria**<br>if they reported a history of cobalamin deficiency, use of cobalamin (>50 ug/d) or folic acid (>200 ug/d) supplementation or injections, surgery or diseases of the stomach or small intestine, anemia, **dementia**, life-threatening diseases, or severe hearing or visual problems. Individuals with an MMSE score <19 points (maximum 30 points) were excluded;
Interventions	Intervention1: 1000 ug vitamin **B-12**<br>Intervention**2**: 1000 ug vitamin B-12 + 400 ug folic acid<br>Placebo: AVICEL PH102 (Medipulp GmbH, Aschaffenburg, Germany) as a filler.
Outcomes	At 6 months

	**Memory**: 15-word learning, delayed recall; Rey complex figure, delayed recall<br>**Executive**: word fluency animal; word fluency letter; WAIS similarities,<br>**Processing speed**: motor planning; finger tapping;<br>**Attention**: digit span backward; digit span forward; TMT A<br>**Visuospatial**: Rey complex figure copy<br>except for all tests of sensomotor speed, motor planning task 3, the Stroop test (part C/part A), and the trail making tests (part 3/part 2), for which a higher score indicates lower cognitive performance.
Notes	**Funding:** Supported by grant 2100.0067 from ZON-MW, The Hague, Netherlands; grant 001-2002 from Kellogg's Benelux, Zaventem, Belgium; grant QLK3-CT-2002-01775 from the Foundation to Promote Research Into Functional Vitamin B12 Deficiency and the European Union BIOMED Demonstration Project; and grant 2004-E2 from the Nutricia Health Foundation, Wageningen, Netherlands.<br>**Compliance**: The participants were asked to maintain their regular diet and to record in a diary their daily intake of capsules, use of medication, and occurrence of any new illnesses during the trial. Compliance was checked by counting the number of unused capsules remaining in capsule dispensers and by verifying pill counts in the participants' diaries.

ROB

Bias	Judge	Support for judgement
Random sequence generation (selection bias)	Unclear	Quote: Randomization was stratified according to MMA concentration at the screening visit, age, sex, and MMSE score.
Allocation concealment (selection bias)	Unclear	Quote: The study had a double-blind design.
Blinding of participants and personnel (performance bias)	Low	Quote: were identical in appearance, smell, and taste.
Blinding of outcome assessment (detection bias)	Unclear	Quote: The study had a double-blind design.
Incomplete outcome data (attrition bias)	Unclear	Quote: no information
Selective reporting (reporting bias)	Unclear	Quote: no information
Other bias	Low	No other sources of bias identified.

Ford, 2010

Methods	Randomized, double-blind controlled clinical trial, 96 months duration
Participants	Location: Australia<br>Setting of recruitment and treatment: community-representative hypertensive men from a large population-based study of abdominal aortic aneurysm screening (Health in Men Study)<br>Sample size<br>● Number randomized: 150 in intervention, 149 in placebo<br>● Number completed: 118 in intervention, 123 in placebo<br>Participant baseline characteristics<br>● Age in years, mean (SD): 79.3 (2.8) intervention, ; 78.7 (2.7) placebo,<br>● Female sex: intervention, 0; placebo, 0

	- Cognitive function (CN, MCI) – MMSE, mean (SD): intervention, 27.5 (1.8); placebo, 27.6 (1.9)<br>**Inclusion criteria**<br>75 years or older; Men<br>**Exclusion criteria**<br>Beck Depression Inventory (BDI) score ≥ 18 or a Mini-Mental State Examination (MMSE) score ≤ 24, were deemed to have an illness likely to cause severe disability or death within 12 months (for example, metastatic cancer, Parkinson disease, or a history of stroke), were non-English speaking, lived in residential care facilities, or were already taking vitamin B supplements.
Interventions	**Intervention:** 25mg B6, **2mg folic acid**, 400ug B12 (1 cap qd, morning) for 24 months.<br>**Placebo:** No information
Outcomes	At 6, 12, 18, and 24 months, 96 months (incidence); changes from baseline<br>**Global**: change in ADAS-cog (primary); **MMSE**; TICS<br>**Memory**: California Verbal Learning Test 2 delayed recall<br>**Visuospatial:** Clock Drawing Test ; Digit Cancellation Test (lower score = impairment)<br><br>**Incidence**: risk of cognitive impairment and dementia (TICS ≤ 27 or a recorded diagnosis of dementia in the Western Australian Data Linkage System)<br>**Quality**: Quality of life (Short Form [SF]–36)
Notes	**Funding:** Dr. Ford reports no disclosures. Dr. Flicker serves as Internal Medicine Editor for Geriatric Medicine, Associate Editor for BMC Geriatrics, on the editorial board of the Australasian Journal on Ageing, and as Editor of the Cochrane Dementia and Cognitive Improvement Group; and receives/ has received research support from Pfizer Inc. and NHMRC. Dr. Alfonso and J. Thomas report no disclosures. Dr. Clarnette serves on scientific advisory boards for Lundbeck Inc., Pfizer Inc., and Novartis; serves as an editorial advisor to Geriatric Medicine in General Practice; and estimates that 10% of his practice at Fremantle Hospital consists of cognitive testing. Dr. Martins serves as Senior Editor for the Journal of Alzheimer's Disease; serves as a consultant for and holds stock in Alzhyme Ltd.; and receives research support from Commonwealth Scientific and Industrial Research Organisation (Australia). Dr. Almeida has received funding for travel from Blackmores Ltd.<br>**Compliance:** Men who consumed at least 75% of the study tablets during this 2-year trial were considered compliant. Compliance was determined by pill count and medication diaries.<br>Twenty-four–month compliance with treatment was 112/150 (74.7%) for men treated with vitamins and 112/149 (75.2%) for men treated with placebo. There was no difference between the groups in the primary outcome of interest when the data from only compliant men were reanalyzed (p = 0.602; data not shown).

ROB

Bias	Judge	Support
Random sequence generation	Low	Quote: Participants were given consecutive numbers and allocated to active vs placebo arms based on computer-generated random

(selection bias)		permuted blocks. Blocks consisted of 8 subjects (4 subjects allocated to each group) so as to minimize the risk of having unbalanced entry into each arm of the study during the period of recruitment.
Allocation concealment (selection bias)	Low	Quote: An external and independent academic controlled the randomization procedures of the trial.
Blinding of participants and personnel (performance bias)	Low	Quote: Vitamins and placebo were administered in the form of identical oral capsules.<br>Participants and investigators were blinded to the group membership of men in the trial until the last follow-up assessment was completed. There were no breaches of protocol.
Blinding of outcome assessment (detection bias)	Low	Quote: Vitamins and placebo were administered in the form of identical oral capsules.<br>Participants and investigators were blinded to the group membership of men in the trial until the last follow-up assessment was completed. There were no<br>breaches of protocol.
Incomplete outcome data (attrition bias)	Low	Quote: Thirty-two (21%) men in the intervention and 26 (17%) in the placebo group withdrew consent or were lost during the trial (χ^2 = 0.72, p = 0.396, df = 1).
Selective reporting (reporting bias)	Low	Quote: The trial was registered with the Australian Clinical Trials Registry (http:// www.actr.org.au), trial number ACTRN012605000045617.
Other bias	Low	No other sources of bias identified.

Garcia, 2004

Methods	**Letter to the editor**<br>-month, double-blind, randomized, placebo-controlled study
Participants	Location: United States<br>Setting of recruitment and treatment: recruited at senior community group meetings and activities.<br>Sample size<br>● Number randomized: 10 in intervention, 14 in placebo<br>● Number completed: 10 in intervention, 12 in placebo<br>Participant baseline characteristics<br>● Age in years: overall mean age 76<br>● Female sex: overall 17 (77%)<br>● Cognitive function (CN) – N/A<br>**Inclusion criteria**<br>- normal cobalamin, elevated methylmalonic acid, and normal cognitive function at baseline, normal renal function.<br>**Exclusion criteria**<br>- subjects taking more than 37.5 mg/d of oral Cbl or any injected dose, history of ileal/gastric surgery, renal failure (serum creatinine level 4130mmol/L), <u>neurological disease (e.g., dementia, stroke</u>, severe head trauma, Parkinson’s disease), depression (based on a Geriatric Depression Rating Scale score 46/15), MMSE score of less than 24 of 30, hospitalization during the 3 months before testing, and any acute medical condition.
Interventions	Intervention: monthly intramuscular injections of 1,000 ug of Cbl for 6 mo.<br>Placebo: saline injection (vitamin B12)

Outcomes	At 6 months.<br>Global: **MMSE**, dementia rating scale<br>Episodic memory: **CVLT trial A list 1-5**<br>Executive function: **Stroop**
Notes	**Funding: N/A**<br>Oral multivitamin use was allowed during the trial, provided Cbl intake was not higher than 25 mg/d.<br>Four persons allocated to the placebo arm and two to the Cbl arm had elevated tHcy (413.9 mM) at baseline.<br>**Compliance**: There were no significant differences in any of the laboratory determinations at baseline between the participants in the Cbl arm and the ones in the placebo arm.

ROB

Bias	Judge	Support for judgement
Random sequence generation (selection bias)	Unclear	Comment: The parent study article by Garcia et al. 2004 had a cross-sectional study design, and did not provide further information about the specifics of the design of the randomized placebo-controlled trial.
Allocation concealment (selection bias)	Unclear	Comment: No information
Blinding of participants and personnel (performance bias)	Unclear	Comment: No information
Blinding of outcome assessment (detection bias)	Unclear	Comment: No information
Incomplete outcome data (attrition bias)	High	Comment: Discrepant drop-out rates between intervention and placebo arms.
Selective reporting (reporting bias)	Unclear	Comment: No information
Other bias	Unclear	Comment: Funding sources not identified

Jiang, 2013

Methods	Randomized clinical trial
Participants	Location: China<br>Setting of recruitment and treatment: They were all patients with cerebral apoplexy that received treatment at the First Hospital Affiliated to the Chinese PLA General Hospital.<br>Sample size<br>● Number randomized: 60 in intervention, 60 in placebo<br>● Number completed: no information<br>Participant baseline characteristics<br>● Age in years, mean (SD): Overall, average age of 63± 1.87 years.<br>● Female sex: Overall, 78 were males and 42 were females<br>● Cognitive function (MCI) – MoCA, mean (SD): intervention, 22.39 (2.01); placebo, 22.50 (2.12)<br>**Inclusion criteria**<br>**Hyperhomocysteinemia (The normal Hcy concentration range is between 5 mmol/L and 14 mmol/L. When a value is above the upper limit, HHcy will be accounted for.);** VCIND was diagnosed based on the recommendations by Rockwood et al. The inclusion criteria included: 1)

	cerebrovascular disease; 2) evidence of cognitive impairment according to psychological evaluation; 3) cognitive impairment within 3 months after cerebral apoplexy; 4) causality between cerebrovascular disease and cognitive impairment, other than other diseases; 5) Hanchinski ischemia index ≥7; and 6) severity without meeting the diagnostic criteria of dementia.<br>**Exclusion criteria**<br>1) Alzheimer disease; 2) other cognitive disorders, mental diseases, or aphasia that affects Montreal cognitive assessment (MoCA) and P300 determination; 3) administration of drugs that influence Hcy level within one month (such as contraceptives, antiepileptics, dopaminergics, and folic acid and/or vitamin B12; and 4) systemic diseases that influence the function of the central nervous system, such as thyroid disease, severe ischemia, deficiency of vitamin B12 and folic acid, severe malnutrition, and severe cardiac, hepatic and renal diseases. Patients with especially bad habits such as alcoholism were excluded.
Interventions	Intervention: 5 mg of folic acid per day and 500 ug of VitB12 thrice per day<br>Placebo: The control group only received conventional treatment.
Outcomes	At 6 months<br>Montreal cognitive assessment (MoCA)
Notes	Funding: no information

ROB

Bias	Judge	Support for judgement
Random sequence generation (selection bias)	Unclear	Quote: The patients were equalized randomly into intervention and control groups.
Allocation concealment (selection bias)	Unclear	No information
Blinding of participants and personnel (performance bias)	High	Quote: Control group did not receive placebo-pills
Blinding of outcome assessment (detection bias)	Unclear	No information
Incomplete outcome data (attrition bias)	Unclear	No information
Selective reporting (reporting bias)	Unclear	No information
Other bias	Unclear	No information on funding sources.

Kang 2008

Methods	2x2x2 randomized placebo-controlled trial of 3 antioxidants (vitamin E, vitamin C, and beta-carotene); In April 1998, a fourth arm including folic acid (2.5 mg/d), vitamin B-6 (50 mg), and vitamin B-12 (1 mg/d) was added
Participants	Location: United States<br>Setting of recruitment and treatment: 1995-96. **Women's Antioxidant Cardiovascular Study**; From December 1998 to July 2000, a mean of 1.2 y after B vitamin randomization, a substudy of cognitive function was initiated among active WAFACS participants; women completed the initial telephone cognitive assessment.<br>Sample size<br>• Number randomized: 1,002 in intervention, 1,007 in placebo

	• Number completed: 521 in intervention, 532 in placebo<br>Participant baseline characteristics<br>• Age in years, mean (SD): intervention, 71.3 (4.2); placebo, 71.3 (4.2)<br>• Female sex: intervention, 100%; placebo, 100%<br>• Cognitive function (CN, MCI) – TICS, mean (SD): intervention, 34.35 (0.10); placebo, 34.38 (0.10)<br>**Inclusion criteria**<br>Female health professionals aged 65 or more; **with CVD** or 3 or more coronary risk factors in 1998; **CVD included** myocardial infarction**, stroke**, revascularization procedures (percutaneous transluminal angioplasty, coronary artery bypass graft, carotid endarterectomy, and peripheral artery surgery), and **symptomatic angina pectoris or transient cerebral ischemia**. Risk factors included current tobacco use, hypertension, high cholesterol, diabetes, parental history of premature myocardial infarction, or obesity [body mass index 30 or more (in kg/m2)]<br>In a 3-mo run-in phase to assess compliance, women received placebo caplets. Women who reported good compliance; who had no history of cancer, active liver disease, chronic kidney failure, or use of anticoagulants; and who expressed willingness to forego the use of out-of-study vitamin supplements (an exception was made for vitamin supplements, including multivitamins that provided only up to the Recommended Dietary Allowances; any supplements that exceeded Recommended Dietary Allowances were not permitted) were randomly assigned to treatment.
Interventions	Intervention: combination **of B vitamins** (2.5 mg folic acid/d, 50 mg vitamin B-6/d, and 1mg vitamin B-12/d)<br>Placebo: no information
Outcomes	At up to 4 times over 5.4 y **(24 (wave 2)**, 48, **72 (wave 4)**,months)<br>Global: TICS<br>Memory: verbal memory (the delayed recall of the TICS 10-word list and the immediate and delayed recalls of the East Boston Memory Test)<br>Executive: categorical fluency
Notes	Funding: Supported by grants AG15933 and HL47959 from the National Institutes of Health.<br>Compliance: Women completed mailed questionnaires annually to update information on compliance, side effects, health and lifestyle characteristics. The average compliance during follow-up was 83% and did not differ significantly between the 2 groups

ROB

Bias	Judge	Support for judgement
Random sequence generation (selection bias)	Unclear	Comment: no information
Allocation concealment (selection bias)	Unclear	Comment: no information
Blinding of participants and personnel (performance bias)	Unclear	Comment: no information
Blinding of outcome assessment (detection bias)	Unclear	Comment: no information
Incomplete outcome data (attrition bias)	Low	Quote: Follow-up rates were nearly identical across treatment groups at each assessment.
Selective reporting (reporting	Unclear	Comment: no information

bias)		
Other bias	Low	No other sources of bias identified

Kwok, 2017

Methods	a randomized placebo-controlled trial
Participants	Location: Hong Kong<br>Setting of recruitment and treatment: recruited from medical/diabetic clinics in Prince of Wales Hospital (PWH) and the seven Family Medicine/general outpatient clinics in the New Territories East cluster in Hong Kong. Between August 2011 and September 2013.<br>Sample size<br>● Number randomized: 137 in intervention, 134 in placebo<br>● Number completed: 109 in intervention, 113 in placebo<br>Participant baseline characteristics<br>● Age in years, mean (SD): intervention, 74.67 (4.00); placebo, 75.84 (4.15)<br>● Female sex: intervention, 55 (40.1%); placebo, 58 (43.3%)<br>● Cognitive function (CN, MCI) – MMSE, mean (SD): intervention, 25.33 (3.79); placebo, 25.2 (3.35)<br>**Inclusion criteria**<br>Diabetic people aged 70 years or over<br>**Exclusion criteria**<br>Those with known dementia or peripheral neuropathy or anemia, disabling stroke, renal failure, clinical depression and those who were taking vitamin B12 supplements or centrally acting medications, and those who did not have a family member who could reliably inform on cognitive functioning (personal contact at least once a week). Those with CDR score of 1 or more (i.e. clinical dementia) or clinical depression, positive titre of intrinsic factor antibody and anemia (HB < 10 g/dl)
Interventions	once daily for **27** months<br>Intervention: two **methylcobalamin** 500 mcg tablets<br>Placebo: two similar looking placebo tablets
Outcomes	At **9, 18, 27** months<br>**Global**: total NTB (neuropsychological test battery) z score<br>**Memory:** International Shopping List Test + Continuous Paired Associates Learning<br>**Executive function:** Controlled Oral Word Association Test + Category Fluency Test (to name animals, vegetables and fruits in 1 min each)<br>**Psychomotor speed:** 'Detection' a test of simple reaction time test (SRT) + 'Identification' a choice reaction time test (CRT)
Notes	**Funding**: General Research Grant from the Hong Kong Research Grant Council. (CUHK468110).<br>Those with borderline low vitamin B12 (150-300 pmol/L) were screened.<br>**Compliance:** RA performed a pill count and dispenses the trial tablets.

ROB

Bias	Judge	Support for judgement
Random sequence generation (selection bias)	Low	Quote: The randomization was performed by a research pharmacist using a computer program with random number generator in blocks of 4 or 6. The

		pharmacist had no contact or knowledge of the trial subjects.
Allocation concealment (selection bias)	Low	Quote: The pharmacist packaged the trial tablets for each month into plain bottles labeled with the name of subject and the subject number. The pharmacist concealed the group assignment from the investigators and research assistants involved in the trial.
Blinding of participants and personnel (performance bias)	Low	Quote: two similar looking placebo tablets
Blinding of outcome assessment (detection bias)	Unclear	Comment: No mention of blinding of researchers.
Incomplete outcome data (attrition bias)	Low	Quote: Five subjects reported side effects and withdrew from the study. The side effects included: skin rash (N=1), headache (N=1), bone pain (N=1), bloating (N=1) in supplement group and dizziness (N=1) in placebo group.<br>Comment: similar rates of drop out between the two arms, and their respective reasons were given in detail.
Selective reporting (reporting bias)	Low	Quote: was registered at the Clinical trial registry of the US (NCT02457507).
Other bias	Low	Comment: no other sources of bias identified.

Lee HK 2016

Methods	A quasi-experimental pretest-posttest control group design
Participants	Location: South Korea<br>Setting of recruitment and treatment: living in care facilities in Gyeong-gi-do, Korea. Samples in study criteria were included with convenience sampling in this study; The period for data collection was February to September 2013<br>Sample size<br>● Number randomized: 24 in intervention, 24 in placebo<br>● Number completed: no information<br>Participant baseline characteristics<br>● Age in years, mean (SD): intervention, 76.08 (5.15); placebo, 78.33 (4.98)<br>● Female sex: intervention, 15 (62.5); placebo, 13 (54.17)<br>● Cognitive function (CN, MCI; score of 20–23 on the Mini Mental State Examination) – MMSE, mean (SD): intervention, 19.29 (7.09); placebo, 17.57 (5.40)<br>**Inclusion criteria**<br><u>65 years of age and older</u>
Interventions	Intervention: Multivitamin supplements as experimental treatment consisted of **<u>vitamin B6, B12, and folic acid</u>**. Multivitamin supplements were taken at a dosage of one pill every day for 12 weeks through the oral route. (no information on the detailed dosage of vitamins)<br>Placebo: no information
Outcomes	At 3 months

	Global; MMSE
Notes	Funding: no information

ROB

Bias	Judge	Support for judgement
Random sequence generation (selection bias)	High	Quote: Participants in Facility A were assigned to the experimental group and those in Facility B to the control group.
Allocation concealment (selection bias)	High	Quote: Participants in Facility A were assigned to the experimental group and those in Facility B to the control group.
Blinding of participants and personnel (performance bias)	Unclear	Comment: no information
Blinding of outcome assessment (detection bias)	High	Quote: Taking multivitamin supplements as an experimental intervention was applied to the experimental group by the researcher.
Incomplete outcome data (attrition bias)	Unclear	Comment: no information
Selective reporting (reporting bias)	Unclear	Comment: no information
Other bias	Unclear	Comment: the cognitive function of participants were designated as MCI without using any consensus criteria.

Lewerin 2005

Methods	a placebo-controlled randomized study
Participants	Location: Sweden<br>Setting of recruitment and treatment: Community-dwelling subjects<br>Sample size<br>● Number randomized: 126 in intervention, 69 in placebo<br>● Number completed: 115 in intervention, 64 in placebo<br>Participant baseline characteristics<br>● Age in years, mean (SD): intervention, 75.7 (4.7); placebo, 75.6 (4.0)<br>● Female sex: intervention, 88 (70%); placebo, 25 (36%)<br>● Cognitive function (CN, MCI) – all cognitive tests well balanced<br>**Inclusion criteria**<br>Community-dwelling subjects<br>N/A
Interventions	Intervention: 0.5 mg cyanocobalamin, 0.8 mg folic acid, and 3mg vitamin B-6 for 4 months<br>Placebo: received an identical (other than the vitamin content) placebo tablet
Outcomes	At 4 months<br>Memory: visual reproduction<br>Processing speed: digit symbol<br>Attention: DSF, DSB<br>Visuospatial: block design
Notes	**Funding:** Supported by grants from the Hjalmar Svensson Foundation, the Göteborg Medical Society, the Medical Faculty at Göteborg University, the

	Wilhelm and Martina Lundgren Foundation, and the Magnus Strandqvist Foundation. Recip AB supported the study and provided the vitamin and placebo tablets.<br>-high plasma total homocysteine (tHcy) concentration (>16umol/L) was found in 64% of men and in 45% of women,<br>-high serum methylmalonic acid (MMA) concentration (>0.34 umol/L) was found in 11% of both sexes.<br>-**compliance:** To ensure compliance, all subjects received a specified blinded number of tablets, and at the end of the study, the number of remaining tablets was compared with the initial number and planned intake during the study period.

ROB

Bias	Judge	Support for judgement
Random sequence generation (selection bias)	Unclear	No information
Allocation concealment (selection bias)	Unclear	No information
Blinding of participants and personnel (performance bias)	Low	Quote: received an identical (other than the vitamin content) placebo tablet
Blinding of outcome assessment (detection bias)	Unclear	No information
Incomplete outcome data (attrition bias)	Low	Quote: Dropouts and excluded subjects (n=48) were slightly older than and had longer MTs and L phases than did the remaining participants (n=161).<br>Comment: Reasons of drop-outs were given.
Selective reporting (reporting bias)	Unclear	No information
Other bias	Unclear	Comment: details of inclusion criteria are not given.

Ma, 2016

Methods	A single-center, randomized Controlled Trial, 12 months duration
Participants	Location: Tianjin, China<br>Setting of recruitment and treatment: six geographically convenient communities with a high proportion of older residents who were all native Chinese speakers were selected from the Binhai New District, Tianjin, China. Enrolled between March 2013 and April 2013. Ninety-seven percent of participants in both the intervention and control groups were living in the community at recruitment.<br>Sample size<br>● Number randomized: 90 in intervention, 90 in placebo<br>● Number completed: 77 in intervention, 75 in placebo<br>Participant baseline characteristics<br>● Age in years, mean (SD): intervention,73.71 (2.57); placebo, 73.52 (3.03)<br>● Female sex: intervention, 57/84 (67.86%); placebo, 58/84 (69.05%)<br>● Cognitive function (MCI; modified Petersen´s criteria) –MMSE, mean (SD): intervention, 25.60 (2.22); placebo, 26.13 (2.51)<br>**Inclusion criteria**<br>(a) aged 65+; (b) absence of terminal illness or mental disorders (ie, major

	depression, schizophrenia, bipolar disorder, etc); (c) not using any nutritional supplementation known to interfere with nutrition status, folate metabolism, or cognitive function in the 3 months before recruitment; and (d) not living in a nursing home or being on a waiting list for a nursing home; MCI by modified Petersen´s criteria
Interventions	Intervention: **folic acid** 400ug/day, during, or immediately after a meal for 12 months<br>Placebo: conventional treatment
Outcomes	At 6, **12** months.<br>Chinese version of WAIS-R<br>**Global:** FSIQ<br>**Attention: Digit Span**<br>**Processing speed**: **Digit Symbol**<br>**Visuospatial:** Block Design, Picture Completion, Object Assembly, Picture Arrangement
Notes	**Funding**: This study was also supported by a grant from the National Natural Science Foundation of China (grant number: 81130053).<br>**Compliance**: Adherence was encouraged and monitored in both groups throughout the trial by telephone interviews at 15 time points and by blood assay at the baseline, six- and twelve-month assessments.<br>**Possible population overlapping with Ma 2019 study (see below)**: The Ma 2016 and Ma 2019 trials have an identical sampling location and design recruited approximately 3 years apart. They did not mention whether these two studies have independent population. Therefore, we concluded that there is a possibility that some individuals who had participated in the Ma 2016 trial could also participate in the Ma 2019 trial after 3 years.<br><br>Ma F, Li Q, Zhou X, Zhao J, Song A, Li W, Liu H, Xu W, Huang G. Effects of folic acid supplementation on cognitive function and Aβ-related biomarkers in mild cognitive impairment: a randomized controlled trial. European journal of nutrition. 2019 Feb 1;58(1):345-56. doi: 10.1007/s00394-017-1598-5

ROB

Bias	Judge	Support
Random sequence generation (selection bias)	Low	Quote: The study sponsor generated the randomization sequence with a computer.
Allocation concealment (selection bias)	High	Quote: Because there was no feasible way to blind the patients' group allocations, it may not be feasible or ethical to provide a sham procedure to make blinding possible.
Blinding of participants and personnel (performance bias)	High	Quote: Conventional treatment: not likely to have been taken identical pills as the intervention group.
Blinding of outcome assessment (detection bias)	Unclear	Quote: However, minimizing measurement bias in this situation may be best accomplished by recruiting a trial investigator, outcome assessors, and data analysts in an attempt to decrease biased classification of the outcomes or unexpected side effects.
Incomplete outcome data (attrition bias)	Low	Quote: Dropout rates were 8.33% (7/84) in the folic acid group and 10.7% (9/84) in the conventional-treatment group. There was no significant difference in dropout

		rates between the two groups ($\chi2$ = 0.276, P = 0.834).
Selective reporting (reporting bias)	Low	Quote: This trial has been registered on May 4th 2013 with trial number ChiCTR-TRC-13003227 (http://www.chictr.org.cn/showproj.aspx?proj= 6332).
Other bias	Low	Comment: No other sources of bias identified.

McMahon, 2006

Methods	Two-year, double-blind, placebo-controlled, randomized clinical trial
Participants	Location: Dunedin, New Zealand,<br>Setting of recruitment and treatment: recruited from service clubs (e.g., Rotary International), through advertisements in newspapers, and by direct mail. Between August 2002 and December 2004<br>Sample size<br>● Number randomized: 138 in intervention, 138 in placebo<br>● Number completed: 127 in intervention, 126 in placebo<br>Participant baseline characteristics<br>● Age in years, mean (SD): intervention, 73.6 (5.8); placebo, 73.4 (5.7)<br>● Female sex: intervention, 47/127 (37%); placebo, 65/126 (52%)<br>● Cognitive function (CN, MCI) – MMSE, mean (SD): intervention, 29.19 (0.97); placebo, 29.17 (1.06)<br>**Inclusion criteria**<br>65 years of age or older; fasting homocysteine concentration of at least 13 μmol per liter and a normal plasma creatinine concentration (133 μmol per liter [1.5 mg per deciliter] in men and ≤115 μmol per liter [1.3 mg per deciliter] in women)<br>**Exclusion criteria**<br>Were ineligible if they had suspected dementia; were taking medications known to interfere with folate metabolism (e.g., oral hypoglycemic agents or antiepileptic agents); were taking vitamin supplements containing folic acid, vitamin B12, or vitamin B6; were being treated for depression; had diabetes; or had a history of stroke or transient ischemic attacks.
Interventions	Intervention: folate (1000 μg) and vitamins B12 (500 μg) and B6 (10 mg) daily for 24 months<br>Placebo: Pill with a blend of magnesium stearate and microcrystalline cellulose as a filler
Outcomes	At 12, 24 months<br>**Global:** MMSE<br>**Memory**: Wechsler Paragraph Recall test (total score on two 25-item tests; maximum possible, 50); Rey Auditory Verbal Learning Test, trials I–V (sum of five trials with the same list; maximum possible, 75 words); Rey Auditory Verbal Learning Test, trial VII (30-min delayed recall; maximum possible, 15 words)<br>**Executive**: Category Word Fluency test (total no. of words generated in three 1-min tests); Controlled Oral Word Association Test (total no. of words generated in three 1-min tests); Raven's Progressive Matrices (20-item test; maximum possible score, 20)
Notes	Funding: Supported by an Otago Research Grant. No potential conflict of interest relevant to this article was reported. We are indebted to Merck Eprova for providing the vitamin and placebo capsules.<br>Compliance: Compliance was assessed by counting returned capsules.

ROB

Bias	Judge	Support
Random sequence generation (selection bias)	Low	Quote: Random decimals between 0 and 1 were generated for each person in each of the four strata. Those below the median of the random numbers in each stratum were assigned to the vitamin group, and the remainder were assigned to the placebo group.
Allocation concealment (selection bias)	Unclear	No information
Blinding of participants and personnel (performance bias)	Low	Quote: All capsules were gelatincoated, identical in color and shape, and packaged in blister packs.
Blinding of outcome assessment (detection bias)	Unclear	Quote: One of the authors administered all the cognitive tests. The tests were conducted in the same order during each session, and whenever possible, the one-year and two-year tests for a given participant were carried out at the same time on the same day of the week as the baseline tests.
Incomplete outcome data (attrition bias)	Low	Quote: Excluded because they had a fasting plasma homocysteine level of less than 13 μmol per liter (172 people) or an abnormal plasma creatinine level (3 people) (Fig. 1). An additional 14 people declined to participate in the intervention after screening. The remaining 276 people were randomly assigned — 138 to the vitamin group and 138 to the placebo group. Three participants withdrew before baseline values were collected. Twelve participants in the placebo group and 11 in the vitamin group were lost to follow-up. Fifteen participants discontinued taking the supplements but completed the tests of cognition and were included in the final analysis.
Selective reporting (reporting bias)	Low	Quote: Australian Clinical Trials registry number, ACTR NO 12605000030673.)
Other bias	Low	No other sources of bias identified.

Moore, 2018

Proceedings of the Nutrition Society

Methods	A randomised controlled trial
Participants	Location: Ireland<br>Setting of recruitment and treatment: Brain Health in Older People (BrainHOP)<br>Sample size<br>● Number randomized: 124 in intervention, 125 in placebo<br>● Number completed: Of the 328 participants initially recruited, 249 (74%) participants completed the intervention.<br>Participant baseline characteristics<br>● Age in years, mean (SD): intervention, 77.9 (4.2); placebo, 78.2 (4.7)<br>● Female sex: intervention, 33%; placebo, 20%<br>● Cognitive function (CN, MCI)<br>**Inclusion criteria**

	Adults aged 70 years and older.
Interventions	Intervention: for 24 months, **folic acid (400 µg), vitamin B12 (**10 µg), vitamin B6 (10 mg) and riboflavin (10 mg) daily<br>Placebo: no information
Outcomes	At 24 months<br>**Global**: RBANS (Repeatable Battery of the Assessment of Neuropsychological Status)<br>**Executive:** FAB (Frontal Assessment Battery)<br>**Visuospatial:** RBANS-Index II
Notes	Funding: N/A

ROB

Bias	Judge	Support for judgement
Random sequence generation (selection bias)	Unclear	Comment: no information (proceedings)
Allocation concealment (selection bias)	Unclear	Comment: no information (proceedings)
Blinding of participants and personnel (performance bias)	Unclear	Comment: no information (proceedings)
Blinding of outcome assessment (detection bias)	Unclear	Comment: no information (proceedings)
Incomplete outcome data (attrition bias)	Unclear	Comment: no information (proceedings)
Selective reporting (reporting bias)	Unclear	Comment: no information (proceedings)
Other bias	Unclear	Comment: no information (proceedings)

Scott, 2017

Methods	a randomized, placebo-controlled multi-site trial
Participants	Location: 18 sites across North America.<br>Setting of recruitment and treatment: from 30 sites in the USA, Canada and Brazil between August 2002 through January 2007, with follow-up contacts occurring every 6 months and annual clinic visits. Of these, 18 sites in North America conducted in-person cognitive testing for the ancillary study.<br>Sample size<br>● Number randomized: 250 in intervention, 274 in placebo<br>● Number completed: 60 in intervention, 71 in placebo<br>Participant baseline characteristics<br>● Age in years, mean (95% CI): intervention, 52.9 (51.7-54.1); placebo, 52.5 (51.4-53.6)<br>● Female sex: intervention, 35.2%; placebo, 38.7%<br>● Cognitive function (**CN, MCI**) – word list delayed recall, mean (95% CI): intervention, 7.2 (6.6-7.8); placebo, 6.7 (6.1-7.4)<br>**Inclusion criteria**<br>aged 35 to 75 years with stable kidney function for at least 6 months after transplantation and with elevated tHcy of ≥12.0 µmol/L for men or ≥11.0 µmol/L for women.<br>**Exclusion criteria**<br>exclusions for visual or hearing impairment substantial enough to hinder performance on cognitive testing.

Interventions	**Intervention:** daily multi-vitamin containing high-doses of folate (5.0 mg), vitamin B12 (1.0 mg) and vitamin B6 (50 mg)<br>**Placebo:** daily multi-vitamin containing no folate and doses of vitamins B12 and B6 consistent with recommended daily allowances (folate 0 mg; vitamin B12, 2.0 μg; and vitamin B6, 1.4 mg<br>Mandatory fortification of flour with folic acid was in effect in the participant's countries throughout the trial.
Outcomes	At mean 39 months<br>Memory: word list delayed recall<br>Executive function: TMT B<br>Processing speed: digit symbol<br>Attention: TMT A<br>Visuospatial: block design
Notes	**Funding:** This research was supported by grant RO1 DK65114 and cooperative agreement UO1 DK61700 from the from the National Institute of Diabetes and Digestive and Kidney Diseases with additional financial support from the Office of Dietary Supplements, National Institutes of Health, Department of Health and Human Services, and by cooperative research agreement 58-1950-4-401 with the U.S. Department of Agriculture. Partial support to AMT was provided by Israel Science Foundation grant no. 1353/11 and a European Union FP7 Marie Curie International Re-integration grant PIRG08-GA-2010-276791 – NUVASCOG.

ROB

Bias	Judge	Support for judgement
Random sequence generation (selection bias)	Unclear	Comment: no information
Allocation concealment (selection bias)	Unclear	Comment: no information
Blinding of participants and personnel (performance bias)	Unclear	Comment: no information
Blinding of outcome assessment (detection bias)	Unclear	Comment: no information
Incomplete outcome data (attrition bias)	Unclear	Comment: high drop-out rates in both arms
Selective reporting (reporting bias)	Low	Quote: registered at ClinicalTrials.gov under identifier NCT00064753.
Other bias	Low	No other sources of bias identified

Stott, 2005

Methods	factorial 2 x 2 x 2, randomized, placebo-controlled, double-blind study with 3 active treatments
Participants	Location: United Kingdom<br>Setting of recruitment and treatment: 2-center, hospital-based<br>Sample size<br>• Number randomized: 23 in intervention, 24 in placebo<br>• Number completed: 23 in intervention, 20 in placebo<br>Participant baseline characteristics<br>• Age in years, mean (SD): intervention, 74.0 (6.5); placebo, 72.8 (5.4)<br>• Female sex: intervention, 14 (61%); placebo, 10 (42%)

	• Cognitive function (CN, MCI) – TICS, mean (SD): intervention, 25.5 (5.2); placebo, 25.1 (4.3)<br>**Inclusion criteria**<br>age 65 or more y and ischemic vascular disease, defined as one or more of the following: history of angina pectoris, previous acute myocardial infarction, evidence of major ischemia or previous acute myocardial infarction on the basis of a 12-lead electrocardiogram, ischemic stroke, transient ischemic attack, intermittent claudication, or surgery for peripheral arterial disease.<br>**Exclusion criteria**<br>an acute vascular event < 1 wk previously; major surgery <1 mo previously; any other major acute illness <1mo previously; severe renal impairment (serum creatinine > 400 umol/L); severe hepatic impairment; malignancy within the previous year (excluding local skin cancer); severe congestive heart failure (New York Heart Association class IV); total anterior cerebral infarct with major residual disability; malabsorption; inability to give informed consent (eg, due to dementia or dysphasia); major cognitive impairment (Mini-Mental State Examination score <19); existing treatment with riboflavin, vitamin B-6, vitamin B-12, or folic acid preparations; hemoglobin concentration < 10 g/dL; and mean cell volume > 100 fL plus either a low red blood cell folate concentration (<280 ng/mL) or a low serum vitamin B-12 concentration (<250 pg/mL).
Interventions	**Intervention:** folic acid (2.5 mg) plus vitamin B-12 (500 ug), vitamin B-6 (25 mg), and riboflavin (25 mg).<br>daily dose was provided in a total of 2 capsules (1 red and 1 white)<br>**Placebo**: no information
Outcomes	At 12 months<br>**Global:** Telephone Interview of Cognitive Status<br>**Processing speed**: Letter Digit Coding Test
Notes	Funding: Supported by a grant from the Healthcare Foundation (reference 112/57).

ROB

Bias	Judge	Support for judgement
Random sequence generation (selection bias)	Low	Quote: Allocation was determined at a site remote from the clinical study (Robertson Centre for Biostatistics) in randomized permuted blocks of 8, stratified by hospital center.
Allocation concealment (selection bias)	Low	Quote: Treatment allocation was concealed from the patients and the investigators (double-blind).
Blinding of participants and personnel (performance bias)	Low	Quote: The capsules were visually identical for the run-in phase and for all arms of the trial.
Blinding of outcome assessment (detection bias)	Low	Quote: Treatment allocation was concealed from the patients and the investigators (double-blind).
Incomplete outcome data (attrition bias)	High	Comment: Selective drop-outs in placebo groups
Selective reporting (reporting bias)	High	Comment: functional outcomes were mentioned in the trial registry, but was not reported in the paper.
Other bias	Low	No other sources of bias identified

Ting, 2017

Methods	**Letter to the editor**<br>a randomized, double-blind international multi-centre trial, longitudinally over 5 years; as per VITATOPS trial protocol
Participants	Location: 20 countries from four continents.<br>International Steering Committee: Clin. Prof. Graeme Hankey (Chairman; Australia), Dr. Christopher Chen (Singapore), Dr. John Gommans (New Zealand), Prof. Kennedy Lees (UK), Dr. Jose Navarro (Philippines), Dr. Udaya Ranawaka (Sri Lanka), Dr. Stefano Ricci (Italy), Dr. Reinhold Schmidt (Austria), Dr. Andrew Slivka (USA) and Dr. Alexander Tsiskaridze (Republic of Georgia).<br>Setting of recruitment and treatment: 123 medical centres<br>Sample size<br>● Number randomized: 118 in intervention, 112 in placebo<br>● Number completed: 33 in intervention, 27 in placebo<br>Participant baseline characteristics<br>● Age in years (range): 68 (61 – 73) intervention, ; 66 (60.8 – 73) placebo,<br>● Female sex: 53 (44.9%) intervention, ; 38 (33.9%) placebo,<br>● Cognitive function (CIND) – MMSE, median (IQR): 24.0 (22.0, 27.0) intervention, ; 25.0 (21.0, 27.0) placebo,<br>**Inclusion criteria**<br>- **had a stroke (ischaemic or haemorrhagic) or transient ischaemic attack** (eye or brain), as defined by standard criteria, within the past 7 months.<br>- a subset of participants with small vessel disease<br>- patients with recent lacunar stroke and cognitive impairment no dementia (CIND): CIND was defined as impairment in at least one domain of the neuropsychological test battery using education adjusted cut-off values of 1.5 SDs below the established normal means on individual tests.<br>**Exclusion criteria**<br>- if they were taking folic acid, vitamin B6, vitamin B12, or a folate antagonist (eg, methotrexate)<br>- pregnant or were women of childbearing potential<br>- if they had a limited life expectancy (eg, because of ill health).
Interventions	Intervention: **B vitamins** (2 mg folic acid, 25 mg vitamin B6, and 0·5 mg vitamin B12)<br>Placebo: matching placebo that had the same colour and coating
Outcomes	At **12**, **24,** 36, 48, **60** months<br>**Global: MMSE**<br>**Episodic memory**: **visual memory span forward**<br>**Executive function: category naming**; frontal assessment battery total score<br>**Attention:** digit span backward; **digit span forward**<br>**Visuospatial function: digit cancellation**
Notes	Funding: This work was supported by Singapore Biomedical Research Council and Singapore National Medical Research Council.<br>Serum homocysteine level of the active group was significantly lower throughout the five years follow-up.

ROB

Bias	Judge	Support for judgement
Random sequence generation	Low	Quote: Random allocation was done by use of a

(selection bias)		central 24 h telephone service or an interactive website by use of random permuted blocks stratified by hospital.
Allocation concealment (selection bias)	Unclear	Comment: No information
Blinding of participants and personnel (performance bias)	Low	Quote: Matching placebo that had the same colour and coating.<br>Patients, clinicians, trial coordinators, and outcome investigators were masked to treatment allocation.
Blinding of outcome assessment (detection bias)	Low	Quote: Patients, clinicians, trial coordinators, and outcome investigators were masked to treatment allocation.
Incomplete outcome data (attrition bias)	High	Comment: More than 25% loss from each arm, and no clear reason given in this letter to the editor.
Selective reporting (reporting bias)	Low	Quote: The trial is registered with www.clinicaltrials.gov, NCT00097669.
Other bias	Low	Comment: No other sources of bias identified

van der Zwaluw, 2014

Methods	Multicenter, double-blind, randomized, placebo-controlled trial, 24 months duration
Participants	**Location:** Netherlands<br>**Setting of recruitment and treatment**: Participated in the B-PROOF (B-Vitamins for the Prevention of Osteoporotic Fractures) study. Conducted in 3 research centers in the Netherlands: VU University Medical Center (Amsterdam), Erasmus Medical Center (Rotterdam), and Wageningen University (WU, Wageningen). Recruited via registries of municipalities in surroundings of the research centers.<br>Sample size<br>● Number randomized: 1,516 in intervention, 1,511 in placebo<br>● Number completed: 425 in intervention, 431 in placebo<br>Participant baseline characteristics<br>● Age in years, mean (SD): intervention, 72.6 (5.7); placebo, 72.6 (5.8)<br>● Female sex: intervention, 181/425 (42.6%); placebo, 176/431 (40.8%)<br>● Cognitive function (CN, MCI) – MMSE, mean (IQR): intervention, 29 (27-30); placebo, 29 (28-30)<br>**Inclusion criteria**<br>Age 65 years or older, having an elevated plasma Hcy level (12–50 mmol/L), being competent to make own decisions, and having a compliant tablet intake (≥85%) in the run-in period.<br>**Exclusion criteria**<br>Cancer diagnosis within the last 5 years except for basal cell carcinoma and squamous cell carcinoma, bedridden, serum creatinine level > 150 umol/L, current or recent (<4 months) use of intramuscular injections of vitamin B12 or folic acid supplementations (> 300 umol), and participation in other intervention studies.
Interventions	Intervention: 400 **ug folic acid** and 500 ug vitamin B12 for 24 months + 15 ug (600 IU) of vitamin D3 to ensure normal vitamin D status.<br>Placebo: 15 ug (600 IU) of vitamin D3 to ensure normal vitamin D status.
Outcomes	At 24 months

	**Global:** MMSE;<br>**Memory:** RAVLT–Immediate Recall; RAVLT-Decay (delayed recall - trial 5); RAVLT–Recognition, max 30 words<br>**Executive:** Trail Making Test (part B/part A); Stroop Interference (part 3 2 [part 1 1 part 2/2]); Verbal Fluency–total no.; Stroop 1 and 2 mean, s;<br>**Processing speed**: Trail Making part A, s; Symbol Digit Modalities Test, no. correct;<br>**Attention:** Digit Span forward, max 16 points; Digit Span backward, max 14 points;
Notes	Funding: B-PROOF was supported and funded by The Netherlands Organization for Health Research and Development (ZonMw, grant 6130.0031), the Hague; unrestricted grant from NZO (Dutch Dairy Association), Zoetermeer; MCO Health, Almere; NCHA (Netherlands Consortium Healthy Ageing) Leiden/Rotterdam; Ministry of Economic Affairs, Agriculture and Innovation (project KB-15-004-003), the Hague; Wageningen University, Wageningen; VU University Medical Center, Amsterdam; and Erasmus Medical Center, Rotterdam.<br>Compliance: Every 6 months, participants received new tablets and participants were requested to return any remaining tablets in order to measure compliance.

ROB

Bias	Judge	Support
Random sequence generation (selection bias)	Low	Quote: Randomization was done by an independent person by means of computer-generated randomization numbers in stratified permuted blocks of size 4, stratified by study center, sex, age (65–79 years, ≥80 years), and Hcy levels (12–17 mmol, ≥18 mmol).
Allocation concealment (selection bias)	Unclear	No information
Blinding of participants and personnel (performance bias)	Low	Quote: Participants and employees of the study were blinded until data analyses were finished.
Blinding of outcome assessment (detection bias)	Low	Quote: Participants and employees of the study were blinded until data analyses were finished.<br>Analyses were performed before unblinding of the treatment code.
Incomplete outcome data (attrition bias)	Low	Quote: In the placebo group, 200 participants (14%) discontinued use of tablets, whereas this number was 222 in the B-vitamin group (15%, not significant). Dropouts were older (77.1 years, p , 0.01), had higher median Hcy concentrations (15.2 mmol/L, p , 0.01), had lower MMSE scores (median 27, p , 0.01), and were more likely to be women (16% vs 13% men, p 5 0.01) compared with persons who completed the study.
Selective reporting (reporting bias)	Low	Quote: This trial is registered at clinicaltrials.gov as NCT00696514 and at Netherlands Trial Register as NTR1333.<br>Per-protocol analyses included those participants who were compliant to the study protocol ($80%).

Other bias	Low	No other sources of bias identified.

van Uffelen, 2008

Methods	Double-blind randomized placebo-controlled trial, two by two factorial design, 12 months duration
Participants	Location: Netherlands<br>Setting of recruitment and treatment: General community<br>Sample size<br>• Number randomized: 90 in intervention, 89 in placebo<br>• Number completed: 71 in intervention, 67 in placebo<br>Participant baseline characteristics<br>• Age in years, mean (SD): intervention, 75 (2.8); placebo, 75 (2.9)<br>• Female sex: intervention, 34/78 (43.6%) ; placebo, 33/74 (44.6%)<br>• Cognitive function (MCI) – MMSE, mean (IQR): intervention, 29 (28-29); placebo, 29 (28-29)<br>**Inclusion criteria**<br>community-dwelling adults aged 70-80 with MCI by Petersen criteria; Memory complaints (answer 'yes' to question 'do you have memory complaints', or at least twice answering 'sometimes' on the Strawbridge cognition scale; Objective memory impairment (10 WLT delayed recall ≤ 5 and percentage savings ≤ 100); Normal general cognitive function (TICS ≥ 19 and MMSE ≥ 24); Intact daily functioning (no report of disability in activities of daily living on GARS (Groningen Activity Restriction Scale)-scale, except on the item 'taking care of feet and toe nails'); Absence of dementia (TICS ≥ 19 and MMSE ≥ 24); Being able to perform moderate intensity physical activity, without making use of walking devices, e.g. a rollator or a walking frame; Not using vitamin supplements/ vitamin injections/ drinks with folic acid, vitamins B12 and B6, comparable to the vitamin supplement given in the intervention; Not suffering from epilepsy, multiple sclerosis, Parkinson's disease, kidney disorder requiring haemodialysis, psychiatric impairment; Not suffering from depression as measured by the GDS (cut off ≤ 5); Not using medication for rheumatoid arthritis or psoriasis which interfered with the vitamin supplement; No alcohol abuse (men < 21 drinks a week, women < 15 drinks a week); Not currently living in a nursing home or on a waiting list for a nursing home
Interventions	Intervention: 5 mg folic acid, 0.4mg B12, 50 mg B6, one pill daily, for 12 months<br>Placebo: identically looking
Outcomes	At 6, 12 months<br>**Global**: MMSE<br>**Memory:** AVLT (Auditory Verbal Learning Test) 1-5 (words, learning trials [sum of trials 1–5]); AVLT 6 (words, where the subject is read an interference list of 15 new words and must recall as many as possible)<br>**Executive:** SCWT-A (Stroop Colour Word Test-Abridged) task 1 (s); SCWT-A task 2 (s); SCWT-A task 3 (s); VFT (Verbal Fluency Test) (words)<br>**Processing speed:** DSST (Digit Symbol Substitution Test) (symbols)
Notes	**Funding:** The FACT-study (Folate physical Activity Cognition Trial) was funded by Body@Work, Research Center Physical Activity, Work and Health, TNO-VU University Medical Center. External financial support was obtained from the municipality of Alkmaar and the 'Stichting Fonds voor

	het Hart'. VIATRIS provided the FA/B12/B6-pills and placebo-pills. None of the external sources had input into protocol development, data collection, analyses and interpretation or drafting this manuscript. We appreciate the assistance of Jos Twisk, PhD, who provided guidance on appropriate statistical methods. We also acknowledge the work of Lyda ter Hofstede and the other research assistants who contributed to the FACT-study.<br>**Compliance:** Compliance was verified by counting pills in returned blister packs

ROB

Bias	Judge	Support
Random sequence generation (selection bias)	Low	Quote: Participants were randomized using the option 'random sample of cases' in SPSS. Randomization was stratified for physical activity level assessed by the LASA physical activity questionnaire
Allocation concealment (selection bias)	Unclear	No information
Blinding of participants and personnel (performance bias)	Low	Quote: Participants and exercise instructors were blinded to group allocation by being left unaware of which exercise program was supposed to be effective. Participants in the control group received an identical-looking placebo-pill.
Blinding of outcome assessment (detection bias)	Low	Quote: The pills were coded as A or B by the manufacturer. The key was decoded after data-analysis.<br>All cognitive outcome measures were assessed by trained examiners, who were also blinded to group allocation.
Incomplete outcome data (attrition bias)	Low	Quote: Two participants stopped taking vitamin FA/B12/B6-pills after reporting adverse side effects, i.e. sleep problems and increased forgetfulness; one participant discontinued the placebo pills after reporting not feeling well.
Selective reporting (reporting bias)	Low	Quote: Study protocol available at BMC Geriatrics [ISRCTN19227688]
Other bias	Low	Quote: Half of the subjects in the walking program got vitamin B supplements and the other half got placebo supplements.

Walker 2012

Methods	randomized controlled trial (RCT) with a completely crossed 2 x 2 x 2 factorial design
Participants	Location: Australia<br>Setting of recruitment and treatment: The population-based sample was recruited by a direct mailing of a screening survey and consent form to 105,000 randomly selected adults aged from 60 to 74 y whose names, addresses, and dates of birth were obtained from the mail lists provided by the Australian Electoral Commission; the sample comprised federal electorates in 2 cities, Canberra (Australian Capital Territory) and Sydney,

	and a rural location, Wagga Wagga (New South Wales). Recruitment occurred between 22 October 2005 and 4 September 2006<br>Sample size<br>● Number randomized: 447 in intervention, 453 in placebo<br>● Number completed: overall, 777 (only 123 (13.5%) participants withdrawing from the time of randomization to the 24-mo assessment).<br>Participant baseline characteristics<br>● Age in years, mean (SD): intervention, 65.92 (4.30); placebo, 65.97 (4.18)<br>● Female sex: intervention, 266 (59.5%); placebo, 276 (60.9%)<br>● Cognitive function (CN, MCI) – TICS-M total score, mean (SD): intervention, 26.42 (3.87); placebo, 26.67 (3.69)<br>**Inclusion criteria**<br>aged from 60 to 74 y; Selected participants had elevated psychological distress as assessed by the K10 (scores 16); did not engage in physical activity at public health–recommended levels as indicated by International Physical Activity Questionnaire scores; did not take folic acid, vitamin B-12, or vitamin B complex supplements; had no history of dementia, bipolar disorder, or current suicide risk; had competent literacy skills; and did not have a medical condition that would contraindicate exercise or FA use.<br>**Exclusion criteria**<br>Individuals with high likelihood of a depressive disorder with K10 scores of >30 were excluded. Those individuals with low concentrations of red blood cell folate (< 250 nmol/L) and vitamin B-12 (< 130 nmol/ L) and abnormal thyroid stimulating hormone concentrations (0.35–5.0 mU/L) were excluded because participation may have led to potentially adverse outcomes.
Interventions	For the entire 24-mo period.<br>Intervention: daily oral 400 ug folic acid+ 100 ug vitamin B-12 supplementation<br>Placebo: Placebo tablets were manufactured by the same producers of the FA + vitamin B-12 tablets and were identical except for the omission of the active substances under investigation.
Outcomes	**At 12, 24** months<br>Global: TICS<br>Memory: TICS, delayed recall
Notes	**Funding:** Supported by beyondblue: the national depression initiative and the Australian Government Department of Health and Ageing. HC was supported by a National Health and Medical Research Council (NHMRC) fellowship no. 525411. JGW was supported by NHMRC Capacity Building Grant 418020.<br>**Adherence** was monitored by telephone assessment at 14 time points and by blood assay at baseline and at 12- and 24-mo assessments.

ROB

Bias	Judge	Support for judgement
Random sequence generation (selection bias)	Unclear	Quote: Participants were randomised into 1 of the 8 intervention programmes arising from the combination of active or comparison conditions of each intervention type

Allocation concealment (selection bias)	Unclear	No information
Blinding of participants and personnel (performance bias)	Low	Quote: Placebo tablets were manufactured by the same producers of the FA + vitamin B-12 tablets and were identical except for the omission of the active substances under investigation.
Blinding of outcome assessment (detection bias)	Unclear	No information
Incomplete outcome data (attrition bias)	Low	Quote: Of those who were recruited into the trial, the dropout rate was low, with only 123 (13.5%) participants withdrawing from the time of randomization to the 24-mo assessment. There were no significant differences in the proportions of participants in FA + vitamin B-12 and placebo groups who completed the 24-mo interview (chi-square1 = 0.6, P = 0.420).
Selective reporting (reporting bias)	High	Quote: This trial was registered at clinicaltrials.gov as NCT00214682<br>Comment: Did not report all outcomes in the protocol.
Other bias	Low	No other sources of biases identified.

Antioxidant vitamins

Grodstein, 2007

Methods	Randomized in a factorial design to receive aspirin, beta carotene, or placebo for prevention of cardiovascular disease and cancer. PHSII is a randomized, placebo-controlled trial, extending the PHS.
Participants	Location: United States<br>Setting of recruitment and treatment: Participants include those continuing their original beta carotene assignment from the PHS, begun in 1982, and newer recruits randomized as of 1998. invitations to enroll in PHSII were mailed to PHS participants, who remained blinded to beta carotene assignment.<br>Sample size<br>● Number randomized: 2,031 in intervention, 2,021 in placebo<br>● Number completed (not given)<br>Participant baseline characteristics<br>● Age in years, mean: intervention, 70.9 from PHS and 72.9 for newly recruited; placebo, 71.0 from PHS and 72.9 for newly recruited<br>● Female sex: intervention, 0; placebo, 0<br>● Cognitive function (CN, MCI)<br>**Inclusion criteria**<br>Male; older than 65 years; All 7,045 PHSII participants older than 65 years were eligible for the cognitive substudy.<br>**Exclusion criteria**<br>men had no history of cancer, active liver disease, current renal disease, peptic ulcer, or gout.
Interventions	Intervention: beta carotene and other vitamin supplements. The beta carotene arm (50 mg, alternate days) was terminated; follow-up is ongoing

	for the remaining arms.<br>Placebo:
Outcomes	At mean <u>216 months (18 years)</u><br><u>Global:</u> TICS<br><u>Memory:</u> verbal memory (immediate and delayed recall measures of the East Boston Memory Test (EBMT))<br><u>Executive:</u> categorical fluency
Notes	**Funding**: This study was supported by grants from the National Institutes of Health (CA34944, CA40360, CA97193, HL26490, HL34595, and AG15933),<br>and from BASF Corporation (Florham Park, New Jersey), Wyeth (New Jersey), and DMS (New Jersey). Dr Grodstein was partially supported by a New Scholars in Aging award from the Ellison Medical Foundation.<br>**Compliance**: Every 12 months, men were sent questionnaires on compliance and health factors. Thus, in the PHSII, all compliance data were established via mailed questionnaire only.

ROB

Bias	Judge	Support for judgement
Random sequence generation (selection bias)	Unclear	Comment: no information
Allocation concealment (selection bias)	Unclear	Comment: no information
Blinding of participants and personnel (performance bias)	Unclear	Quote: who remained blinded to beta carotene assignment.
Blinding of outcome assessment (detection bias)	Unclear	Comment: no information
Incomplete outcome data (attrition bias)	Low	Quote: Overall, 79.3% reported taking at least 2 of 3 of their study pills; this was similar in those assigned to active treatment (79.1%) or placebo (79.5%), and in the continuing participants from the PHS (79.2%) vs new recruits (79.4%).
Selective reporting (reporting bias)	Low	Quote: A cognitive component was added to the PHSII during the final years of beta carotene treatment.<br>clinicaltrials.gov Identifier: NCT00270647
Other bias	Low	No other sources of bias identified

Kang, 2006

Methods	a randomized, double-blind, placebo-controlled trial
Participants	Location: United States<br>Setting of recruitment and treatment: <u>**Women's Health Study**</u>; begun between 1992 and 1995; In 1998, a mean of 5.6 years after randomization, a substudy of cognitive function was initiated among active WHS participants 65 years or older (n=7175) at that time.<br>Sample size<br>● Number randomized: 3,184 in intervention, 3,193 in placebo<br>● Number completed: 2,596 in intervention, 2,630 in placebo<br>Participant baseline characteristics<br>● Age in years, mean (SD): intervention, 66.2 (4.0); placebo, 66.3 (4.1)

	- Female sex: intervention, 100%; placebo, 100%<br>- Cognitive function (CN, MCI) – TICS, mean (SD): intervention, 34.21 (0.05); placebo, 34.23 (0.05)<br>Inclusion criteria<br>if they were at least 45 years old; had **no history of coronary heart disease, cerebrovascular disease,** cancer (except for non-melanoma skin cancer), or other major chronic illnesses; and did not actively use any of the study medications or have any history of adverse effects from the medications.
Interventions	Intervention: 600 IU [**alpha-tocopherol acetate**], on alternate days<br>Placebo: no information
Outcomes	At 24, 48 months<br>**Global:** TICS<br>**Memory**: verbal memory (the delayed recall of the TICS 10-word list and the immediate and delayed recalls of the East Boston Memory Test)<br>**Executive**: categorical fluency
Notes	Funding: This work was supported by grants CA47988 and AG 15933 from the National Institutes of Health<br>Compliance: Women were asked to complete mailed questionnaires annually to update information on compliance, adverse effects, health and lifestyle characteristics, and the occurrence of clinical end points

ROB

Bias	Judge	Support for judgement
Random sequence generation (selection bias)	Unclear	Comment: no information
Allocation concealment (selection bias)	Unclear	Comment: no information
Blinding of participants and personnel (performance bias)	Unclear	**Quote:** Every 12 months during follow-up, the women were sent a year's supply of monthly calendar packs containing active agents or placebo.<br>**Comment:** clear description of how the participants were blinded to treatment arms were not given.
Blinding of outcome assessment (detection bias)	Unclear	Comment: no information
Incomplete outcome data (attrition bias)	High	Quote: the overall follow-up of the cohort exceeded 99%<br>Comment: actual drop-out rates among cognitive cohort is quite high.
Selective reporting (reporting bias)	Unclear	Comment: no information
Other bias	Low	No other sources of bias identified

Kang 2009

Methods	2 x 2 x 2 factorial, randomised, placebo-controlled trial
Participants	Location: United States and Puerto Rico<br>Setting of recruitment and treatment: **The Women's Antioxidant Cardiovascular Study**; from December 1998 to July 2000, we initiated a substudy of cognitive function<br>Sample size<br>- Number randomized: 1,428 in intervention, 1,396 in placebo for

	vitamin E<br>● Number completed: 1,586 in total for the vitamin E group<br>Participant baseline characteristics<br>● Age in years, mean (SD): intervention, 72.6 (4.3); placebo, 72.5 (4.2)<br>● Female sex: intervention, 100%; placebo, 100%<br>● Cognitive function (CN, MCI) – TICS, mean difference (95% CI): -0.01 (-0.25, 0.23)<br>**Inclusion criteria**<br>65 or more years of age; with cardiovascular disease or 3 or more coronary risk factors; Coronary risk factors included parental history of premature myocardial infarction, diabetes mellitus, hypertension, high cholesterol, and obesity (body mass index 30 or more kg/m2). CVD included myocardial infarction, stroke, revascularization procedures (percutaneous transluminal angioplasty, coronary artery bypass graft, carotid endarterectomy, or peripheral artery surgery), and symptomatic angina pectoris or transient cerebral ischemia.
Interventions	Intervention: **vitamin E** (402 mg every other day), beta carotene (50 mg every other day), or vitamin C (500 mg daily) over 5.4 years<br>Placebo: no information
Outcomes	At 24, 48, 72 months<br>**Global**: TICS<br>**Memory**: verbal memory<br>**Executive**: categorical fluency
Notes	**Funding:** This work is supported by grants AG15933 and HL046959 from the National Institutes of Health.<br>**Compliance:** Women completed annual mailed questionnaires on compliance; When assessed on annual questionnaires, participants' compliance to assigned study agents was high and comparable between the active and placebo groups<br>**Population redundancy with Kang 2006:** This article explicitly stated that, in the discussion section, "Vitamin E has been studied extensively in relation to cognitive function, including several randomized trials in different populations with different durations and dosages (Quoted Kang 2006)."

ROB

Bias	Judge	Support for judgement
Random sequence generation (selection bias)	Unclear	Comment: no information
Allocation concealment (selection bias)	Unclear	Comment: no information
Blinding of participants and personnel (performance bias)	Unclear	**Quote:** Every year during follow-up, the women were sent a 12-month supply of calendar packs containing active agents or placebo
Blinding of outcome assessment (detection bias)	Unclear	Comment: no information
Incomplete outcome data (attrition bias)	Low	Quote: In the fourth assessment, 24% of participants were not contacted for their interview because only a short interval had passed between their third interview and the end of the trial in January 2005.

		Follow-up rates were nearly identical across treatment groups at each assessment.
Selective reporting (reporting bias)	Unclear	Comment: no information
Other bias	Low	No other sources of bias identified

MRC/BHF 2002

Methods	"2x2 factorial" design randomised placebo-controlled study
Participants	Location: 69 Hospitals in the UK<br>Setting of recruitment and treatment: Medical collaborators from 69 UK hospitals appointed senior nurses to run special clinics for the study. Potentially eligible people entered a prerandomisation "run-in" phase, which involved about 2 months of active vitamins.<br>Sample size<br>● Number randomized: 10,269 in intervention, 10,267 in placebo<br>● Number completed: 10,241 in intervention, 10,228 in placebo<br>Participant baseline characteristics<br>● Age in years: 5806 (28.3%) aged at least 70 years at study entry.<br>● Female sex: no information but recruited both sexes.<br>● Cognitive function (CN, MCI)<br>**Inclusion criteria**<br>Men and women aged about 40 years to 80 years with non-fasting blood total cholesterol concentrations of at least 3·5 mmol/L were eligible provided they were considered to be at substantial 5-year risk of death from coronary heart disease because of a past medical history of coronary heart disease, of other occlusive arterial disease, of diabetes mellitus, or of treated hypertension alone. Compliant individuals who did not have a major vascular event or other serious problem during the run-in.<br>**Exclusion criteria**<br>People were ineligible if they had other life-threatening conditions, such as chronic liver disease, severe renal disease, severe heart failure, severe chronic airways disease, or diagnosed cancer (other than non-melanoma skin cancer). In addition, anyone already taking high-dose vitamin E supplements, or in whom such supplements were considered indicated, was not to be randomised.
Interventions	Intervention: 600 mg synthetic vitamin E, 250 mg vitamin C and 20 mg β-carotene administered orally once per day for an average of 5 years<br>Placebo: matching placebo
Outcomes	At 5 year<br>Global: TICS-m score
Notes	Funding: The study was funded by the UK Medical Research Council, the British Heart Foundation, Merck & Co (manufacturers of simvastatin: J Tobert, R Tomiak, J Young, A Tate, E John, F Walker, G Warner) and Roche Vitamins Ltd (manufacturers of the vitamins: R Salkeld, E Stöcklin, M Wahl)<br>Compliance: Compliance with study treatment was assessed at each follow-up by reviewing the calendar-packed capsules remaining and, for those who had stopped, the reasons for doing so were sought.

ROB

Bias	Judge	Support for judgement

Random sequence generation (selection bias)	Low	Quote: were then randomly allocated to receive the antioxidant vitamins or matching placebo capsules in specially prepared calendar packs; The central telephone randomisation system used a minimisation algorithm to balance the treatment groups with respect to eligibility criteria and other major prognostic factors.
Allocation concealment (selection bias)	Low	Quote: The central telephone randomisation system
Blinding of participants and personnel (performance bias)	Unclear	Quote: matching placebo capsules
Blinding of outcome assessment (detection bias)	Low	Quote: All such information was reviewed by coordinating centre clinical staff who were kept unaware of the study treatment allocation, and events were coded according to prespecified criteria.
Incomplete outcome data (attrition bias)	Low	Quote: Similar percentages of participants remained compliant in each treatment group, with the average during the study being 83%<br>Comment: relatively low dropout rates between intervention arms and
Selective reporting (reporting bias)	Low	Quote: the protocol on the study website: www.hpsinfo.org; The data analysis plan was prespecified either in the original protocol or in amendments
Other bias	Low	No other sources of bias identified.<br>The study was designed, conducted, analysed, and interpreted by the investigators entirely independently of all funding sources.

Naeini 2014;

Methods	Double-blind, randomized, placebo-controlled, 12 months duration
Participants	Location: Isfahan, Iran<br>Setting of recruitment and treatment: From retirees clubs<br>Sample size<br>● Number randomized: 127 in intervention, 129 in placebo<br>● Number completed: no information<br>Participant baseline characteristics<br>● Age in years, mean (SE): intervention, 66.5 (0.39); placebo, 66.3 (0.38)<br>● Female sex: intervention, 64/127 (50.4%); placebo, 72/129 (55.8%)<br>● Cognitive function (MCI)<br>Inclusion criteria<br>Aged 60-75 years; MMSE 21-26;<br>Exclusion criteria<br>obvious disabling disease, Alcohol intake, smoking, and routine consumption of neurological or antioxidants drugs.
Interventions	Intervention: 300 mg of **vitamin E** (Dl-alpha-tocopherol acetate) plus 400 mg vitamin C (ascorbic acid) for 12 months<br>Placebo: identical condition
Outcomes	At 6, **12 months**

	**Global:** MMSE<br>(In sixth month: supplemented vs. control 25.88 ± 0.17 vs. 25.86 ± 0.18 and in 12th month 26.8 ± 0.17 vs. 26.59 ± 0.18)
Notes	**Funding:** This study was supported by Institute of Nutritional Sciences, University of Vienna and the Vice-chancellor for Research, Tehran University of Medical Sciences (TUMS), Iran, by a Grant (No. 11126).

ROB

Bias	Judge	Support
Random sequence generation (selection bias)	Unclear	Selection and grouping were performed using stratified method followed by simple randomization method.
Allocation concealment (selection bias)	Unclear	No information
Blinding of participants and personnel (performance bias)	Low	consumed placebo with the identical condition
Blinding of outcome assessment (detection bias)	Unclear	No information
Incomplete outcome data (attrition bias)	Unclear	From these, 40 did not continue with the study due to the problems tolerating the supplementations (14 subjects), 1 subject died, and 25 subjects abstained to continue participation due to personal reasons. antioxidants drugs.
Selective reporting (reporting bias)	Unclear	No information
Other bias	Low	No other sources of bias identified

Peterson, 2005

Methods	multicenter, randomized, double-blind, placebo-controlled, parallel-group study
Participants	Location: from 69 ADCS sites in the United States and Canada.<br>Setting of recruitment and treatment: conducted between March 1999 and January 2004<br>Sample size<br>● Number randomized: 257 in intervention1, 253 in intervention 2, 259 in placebo<br>● Number completed: 185 in intervention1, 161 in intervention2, 193in placebo<br>Participant baseline characteristics<br>● Age in years, mean (SD): intervention1, 72.8 (7.3); intervention2, 73.1 (7.1); placebo, 72.9 (7.6)<br>● Female sex: intervention1, 119 (46%); intervention2, 112 (44%); placebo, 121 (47%)<br>● Cognitive function (aMCI) – MMSE, mean (SD): intervention1, 27.2 (1.9); intervention2, 27.3 (1.8); placebo, 27.4 (1.8)<br>**Inclusion criteria**<br>Amnestic mild cognitive impairment of a degenerative nature (insidious onset and gradual progression), impaired memory, a Logical Memory delayed-recall score approximately 1.5 to 2 SD below an education-adjusted norm, a Clinical Dementia Rating (CDR) of 0.5, a score of 24 to 30 on the

	Mini–Mental State Examination (MMSE), and an **age of 55 to 90 years**.<br>**Exclusion criteria**<br>1. Significant cerebral vascular disease: Modified Hachinski > 4<br>2. Depression: Hamilton Depression Rating Scale > 12<br>3. Central nervous system infarct, infection or focal lesions of clinical significance on CT or MRI scans<br>4. Medical diseases or psychiatric disorders that could interfere with study participation<br>5. Pregnant, lactating or of child bearing potential<br>6. Taking vitamin supplements, other supplements or a multi-vitamin<br>7. Restrictions on concomitant medication usage, including those with significant cholinergic or anticholinergic effects or potential adverse effects on cognition
Interventions	**Intervention1**: 2000 IU **of vitamin E,** placebo donepezil, and a multivitamin daily;<br>Intervention2: 10 mg of donepezil, placebo vitamin E, and a multivitamin daily;<br>**Placebo:** placebo vitamin E, placebo donepezil, and a multivitamin daily.<br>The multivitamin contained 15 IU of vitamin E.<br>The initial dose of vitamin E was 1000 IU daily, and the dose was increased to 2000 IU (1000 IU twice daily) after six weeks.<br>If a subject had difficulty tolerating the higher dose of vitamin E or donepezil, the investigator could reduce the dose of either medication temporarily and then rechallenge with the higher dose.
Outcomes	**At 12, 24, 36** months<br>Global: **MMSE**<br>**Memory**: ADAS immediate and delayed word-recall scores and the New York University immediate and delayed paragraph-recall scores<br>**Visuospatial:** the clock-drawing test<br>The cognitive-domain and overall composite scores were calculated as the weighted sum of the individual standardized test scores. The individual test scores were standardized by dividing each score by the standard deviation of the baseline scores. Weights were calculated as the reciprocal of the sum of the correlation coefficients between the tests in each domain at baseline.
Notes	**Funding:** Fifty percent of the funding was provided by the National Institute on Aging, with the other 50 percent coming from Pfizer and Eisai.<br>**Cognitive test scores:** This study defined the score of 'executive' using digits backward test and Symbol Digit Modalities Test, and number cancellation test which are, in our meta-analysis, categorized into attention, processing speed, and visuospatial function, respectively. Therefore we were unable to use this score in our meta-analysis model due to substantial heterogeneity. This study also defined 'language' score using Boston Naming Test and category-fluency test. However, because our analysis categorized the category-fluency test into Executive function, and we determined the Boston Naming Test cannot appropriately reflect this cognitive domain, we decided not to use this score as well.

ROB

Bias	Judge	Support for judgement
Random sequence generation (selection bias)	Unclear	Quote: were randomly assigned to

Allocation concealment (selection bias)	Unclear	Comment: no information
Blinding of participants and personnel (performance bias)	Unclear	Comment: no information
Blinding of outcome assessment (detection bias)	Unclear	Comment: no information
Incomplete outcome data (attrition bias)	High	**Quote:** A total of 790 subjects underwent randomization, and 769 completed the baseline assessment.<br>Adverse events in the donepezil group included muscle cramps, gastrointestinal symptoms, and sleep disturbances (Table 4). Twenty-three deaths occurred during the study (17 during the double blind phase and 6 during the open-label phase), and all were judged to be unrelated to treatment. During the double-blind phase, seven subjects died in the donepezil group and five subjects died in each of the other two groups (P=0.79).<br>A total of 230 subjects discontinued treatment during the double-blind phase: 92 in the donepezil group, 72 in the vitamin E group, and 66 in the placebo group (P=0.90). Among the leading reasons for discontinuation besides death were adverse events in the case of 47 subjects and withdrawal of consent in the case of 105 subjects.<br>**Comment:** Discrepant rate of the drop-out among study arms
Selective reporting (reporting bias)	Unclear	Comment: no information
Other bias	Low	No other sources of bias identified

Smith (1), 1999

Methods	A double-blind placebo controlled trial
Participants	Location: United Kingdom<br>Setting of recruitment and treatment: recruited by means of advertisements placed in the local press.<br>Sample size<br>● Number randomized: 93 in intervention, 92 in placebo<br>● Number completed: no information<br>Participant baseline characteristics<br>● Age in years, mean (SD): intervention, 66.76 (0.48); placebo, 66.9 (0.56)<br>● Female sex: intervention, 50 (54%); placebo, 50 (54%)<br>● Cognitive function (CN, MCI) – MMSE, mean (SD): N/A<br>**Inclusion criteria**<br>Aged between 60 and 80 years and within two standard deviations of the normal weight for height, age and sex; no history or evidence of significant disease or mental illness; able and willing to give informed consent; capable of taking 80-120 percent of the prescribed number of capsules during the run-in period.

	**Exclusion criteria**<br>Current medication likely to influence the outcome measures; use of vitamin supplements in the preceding 3 months; evidence or history of regular or chronic drug abuse including alcohol; significant cardiovascular, respiratory, hepatic, renal, gastrointestinal, endocrine, neurological disease or abnormality; malabsorption syndrome; psychiatric disorder; subjects unable or unwilling to give informed consent; disorders which would interfere with the understanding or compliance with the study, hypersensitivity to any of constituents in the active treatment; advanced stages of cognitive decline (mini-mental score below 18); participation in another drug clinical trial within the previous 6 months; subjects from whom blood samples could not be obtained.
Interventions	Take two capsules daily for a period of 4 weeks.<br>**Intervention**: 12 mg/d beta carotene, 400 mg/d alpha - tocopherol and 500 mg/d ascorbic acid.<br>Placebo: not specified
Outcomes	At 4, 8 and 12 months.<br>**Episodic memory**: **free recall task**, no of words correctly recalled<br>**Psychomotor speed**: simple reaction time, total mean reaction time (lower score = improvement)<br>**Attention: repeated digits vigilance task**
Notes	**Funding:** This study was supported by F. Hoffman-La Roche Ltd, Basel. We would like to thank Pip Brockman and Barbara Dovy for their assistance with data collection and administration of the study.<br>**Compliance**: Blood samples were taken to determine vitamin levels at these times. Compliance was considered acceptable if they took between 45 and 67 capsules.<br>**Exclusion criteria:** Because this study stated that it excluded those with significant neurologic disease, we determined that the investigators excluded those with dementia as well.

ROB

Bias	Judge	Support for judgement
Random sequence generation (selection bias)	Unclear	Quote: The volunteers were randomly assigned to either placebo or vitamin groups, with stratification by gender.<br>Comment: No specifics about the sequence generation method.
Allocation concealment (selection bias)	Unclear	Comment: No information
Blinding of participants and personnel (performance bias)	Unclear	Quote: subjects were given 70 placebo capsules and told to take two daily for a period of 4 weeks.<br>Comment: No information
Blinding of outcome assessment (detection bias)	Unclear	Comment: No information
Incomplete outcome data (attrition bias)	Unclear	Comment: No information on the number of subjects at each follow-up assessments.
Selective reporting (reporting bias)	Unclear	Comment: No information
Other bias	Unclear	Funding sources not identified.

Smith (2) 1999;

Methods	Randomised, double blind, placebo-controlled study, 12 months duration
Participants	Location: United Kingdom<br>Setting of recruitment and treatment: were recruited by means of advertisements placed in the local press.<br>Sample size<br>• Number randomized: 93 in intervention, 92 in placebo<br>• Number completed: no information<br>Participant baseline characteristics<br>• Age in years, mean (SE): intervention, 66.76 (0.48); placebo, 66.9 (0.56)<br>• Female sex: intervention, 50/93 (53.8%); placebo, 50/92 (54.3%)<br>• Cognitive function (CN, MCI) – NART, mean (SE): intervention, 37.09 (0.9); placebo, 37.23 (0.87)<br>**Inclusion criteria**<br>Aged 60-80 years old; within two standard deviations of the normal weight for height, age and sex; no history or evidence of significant disease or mental illness; able and willing to give informed consent; capable of taking 80-120% of the prescribed number of capsules during the placebo run-in period.<br>**Exclusion criteria**<br>current medication likely to influence the outcome measures; use of vitamin supplements in the preceding three months; evidence or history of regular or chronic drug abuse including alcohol; significant cardiovascular, respiratory, hepatic, renal, gastrointestinal, endocrine, neurological disease or abnormality; malabsorption syndrome; psychiatric disorder; unable or unwilling to give informed consent; disorders which would interfere with the understanding or compliance with the study, hypersensitivity to any of constituents in the active treatment; advanced stages of cognitive decline (mini-mental score below 18); participation in another drug clinical trial within the previous six months; volunteers from whom blood samples could not be obtained.
Interventions	Intervention: 12 mg/d **beta-carotene, 400** mg/d alpha-tocopherol and 500 mg/d ascorbic acid for 12 months<br>Placebo:
Outcomes	At **12** months<br>**Global:** intelligence (New Adult Reading Test- Nelson and O'Connell, 1978)
Notes	**Funding:** This study was supported by F. Hoffman-La Roche Ltd, Basel. We would like to thank Pip Brockman and Barbara Dovy for their assistance with data collection and administration of the study.<br>**Exclusion criteria:** Because this study stated that it excluded those with significant neurologic disease, we determined that the investigators excluded those with dementia as well.

ROB

Bias	Judge	Support
Random sequence generation (selection bias)	Unclear	Quote: The volunteers were then randomly assigned to either placebo or vitamin groups.
Allocation concealment (selection bias)	Unclear	No information

Blinding of participants and personnel (performance bias)	Unclear	No information
Blinding of outcome assessment (detection bias)	Unclear	No information
Incomplete outcome data (attrition bias)	Unclear	No information
Selective reporting (reporting bias)	Unclear	No information
Other bias	Unclear	Quote: A trimmed means procedure (eliminating the extreme 5% scores) was used to deal with outliers.

Yaffe 2004

Methods	4-arm, double-blinded, multicentre parallel group RCT, with up to 8 years of treatment (Age-Related Eye Disease Study (AREDS) report No. 12)
Participants	Location: 11 clinical centres in the US<br>Setting of recruitment and treatment: Between 1992 and 1998, 11 clinical centers enrolled 3,640 participants<br>Sample size<br>● Number randomized: 2737 in intervention, 903 in placebo<br>● Number completed: 1632 in intervention, 534 in placebo<br>Participant baseline characteristics<br>● Age in years, median: intervention, 69; placebo, 69<br>● Female sex: intervention, 55%; placebo, 56%<br>● Cognitive function (CN, MCI)<br>**Inclusion criteria**<br>Aged 55 to 80 years old; Individuals who passed the screening evaluation and were interested in participating in AREDS were provided with a one-month "run-in" supply of placebo to assess potential for tolerance of the inactive ingredients and compliance with the treatment regimen.<br>Participants who had good compliance with the run-in medication, who had adequate pupillary dilation and no disqualifying lesions noted on photographs sent to the Reading Center for grading, and who signed a second consent form were stratified into AMD categories at the time of randomisation<br>**Exclusion criteria**<br>Had to be free of any illness or condition that would make long-term follow-up or compliance with study medications unlikely or difficult; Participants taking fewer than 75% of the prescribed tablets were ineligible for enrolment.
Interventions	Intervention: Group A (Antioxidants): 500mg Vitamin C, 400 IU Vitamin E and 15mg beta carotene daily<br>Placebo: no information<br>They were asked to take two tablets twice a day for 1 month.
Outcomes	after a median of 6.9 years of treatment.<br>**Global:** 3MS<br>**Memory**: logical memory part II, delayed recall<br>**Executive**: animal category<br>**Attention**: digits backwards
Notes	**Funding**: Supported by contracts from the National Eye Institute and the

	National Institute on Aging, National Institutes of Health, Department of Health and Human Services, Bethesda, MD, with additional support from Bausch & Lomb Inc, Rochester, NY.

ROB

Bias	Judge	Support for judgement
Random sequence generation (selection bias)	Low	Quote: Eligibility verification and random treatment assignment were performed by the Coordinating Center using the on-site computers, with procedures to protect the integrity of randomization and to provide randomization backup in case of hardware malfunction. Multiple levels of data encryption ensure the integrity of the treatment assignment files.
Allocation concealment (selection bias)	Low	Quote: Multiple levels of data encryption ensure the integrity of the treatment assignment files; Each treatment assignment database residing on the hard drives at each Clinical Center is encrypted and includes check numbers to insure tamper free operation and proper sequential treatment assignments. There were no cases of database corruption during randomization.
Blinding of participants and personnel (performance bias)	Low	Quote: The AREDS participants, investigators and Reading Center personnel are masked to study-wide outcome data and treatment assignments; Four participants (0.1%) were reported to have been unmasked during the trial.<br>Comment: The proportion of participants who were unmasked during the trial was extremely low.
Blinding of outcome assessment (detection bias)	Low	Quote: The AREDS participants, investigators and Reading Center personnel are masked to study-wide outcome data and treatment assignments.
Incomplete outcome data (attrition bias)	High	Quote: Another limitation is that not all participants in the trial had cognitive testing, although participation in the cognitive ancillary study did not differ by treatment group and so probably does not interfere with our assessment of treatment effects.<br>Comment: Although the drop-out rates between the two treatment arms were comparable (40.4% vs 40.9%), their absolute values were too high.
Selective reporting (reporting bias)	Unclear	No information
Other bias	Low	No other sources of bias identified.

Vitamin D

Aspell, 2017;
Proceedings

Methods	Proceedings<br>a double-blind randomised placebo-controlled study
Participants	Location: Ireland<br>Setting of recruitment and treatment: recruited from Nov 2015 to Man 2016

	Sample size<br>● Number randomized: 30 in intervention, 29 in placebo<br>● A retention rate of 93·8 % was achieved.<br>Participant baseline characteristics<br>● Mean age was 68·5years SD 4·9 and 53·3 % were female.<br>● There were no significant baseline differences between groups<br>● Cognitive function (CN, MCI)<br>**Inclusion criteria**<br>healthy older adults, aged 60 years and older, without cognitive impairment (MMSE <23 or related disease) and **with measured serum vitD <125 nmol/l**<br>**Exclusion criteria**<br>According to the study protocol: Measures low or high serum vitamin D, defined as < 15nmol/L or >125nmol/L; Current use of supplemental vitamin D ≥800 international units/d; Screen positive for cognitive impairment using the Telephone Cognitive Screen (TCogS); Measured hypercalcaemia, defined as corrected serum calcium > 2.7nmol/l; Hyperparathyroidism; Epilepsy; Stroke; Renal disease; Schizophrenia; Bipolar affective disorder; Recurrent psychotic depression; Alcohol and drug abuse within the past 5 years; Anti-convulsants; Anti-psychotic medications; Significant hearing difficulties even when wearing hearing aid; Illness that caused permanent decrease in memory or other mental function
Interventions	Intervention: 50ug/day of **vitD3**<br>Placebo: Placebo -gel capsule containing no vitamin D.
Outcomes	At 6 months<br>**Global:** Montreal Cognitive Assessment<br>**Executive**: Trails Making Task A&B, B-A: TMTA&B, B-A<br>Sustained Attention to response Task_Coefficient of Variation
Notes	Funding: PhD scholarship funding by the Irish Research Council; University of Dublin, Trinity College

ROB

Bias	Judge	Support for judgement
Random sequence generation (selection bias)	Unclear	Comment: no information
Allocation concealment (selection bias)	Unclear	Comment: no information
Blinding of participants and personnel (performance bias)	Unclear	Comment: no information
Blinding of outcome assessment (detection bias)	Unclear	Comment: no information
Incomplete outcome data (attrition bias)	Unclear	Comment: no information
Selective reporting (reporting bias)	High	Quote: This trial was registered at https://clinicaltrials.gov as NCT02804841.<br>Comment: Did not report all the outcomes in the protocol.
Other bias	Unclear	No other sources of bias identified. Proceedings

Hu, 2018

Methods	Occasional Essay

	a population-based randomised, double-blind, placebo-controlled trial for 12 months
Participants	Location: Nankai District, Tianjin, China,<br>Setting of recruitment and treatment: six communities with older residents, by multistage random cluster sampling. The general practitioners of community health centres helped us announce the research purpose and encourage older adults to participate. Enrolled between March 2016 and April 2016<br>Sample size<br>● Number randomized: 93 in intervention, 88 in placebo<br>● Number completed: 80 in intervention, 83 in placebo<br>Participant baseline characteristics<br>● Age in years, mean (SD): 67.22 (6.09) intervention, ; 66.60 (5.24) placebo,<br>● Female sex: 50 (54%) intervention, ; 50 (57%) placebo,<br>● **Cognitive function (MCI; modified Petersen's criteria**) – MMSE, mean (SD): 22.60 (1.94) intervention, ; 22.43 (1.80) placebo,<br>**Inclusion criteria**<br>- all native Chinese speakers<br>- (A) aged 65+; (B) absence of terminal illness or mental disorders (ie, major depression, schizophrenia, bipolar disorder, and so on); (C) not using any nutritional supplementation known to interfere with nutrition status, vitamin D metabolism or cognitive function in the 3 months before recruitment; (D) not having a medical condition that would contraindicate vitamin D3 use; and (E) not living in a nursing home or being on a waiting list for a nursing home.
Interventions	**Intervention:** vitamin **D3** supplementation, one capsule daily during or immediately after a meal. 'Aiweidi' vitamin D3 nutrient solution soft capsule. A daily oral dose of one capsule consisting of **400 IU** vitamin D3 for the entire 12-month period.<br>**Placebo:** starch granules were manufactured by the same producers and were identical except for the omission of the active substances under investigation.
Outcomes	At 6 , **12 months**<br>Global: WAIS-RC, **FSIQ**<br>Processing speed: **WAIS digit symbol substitution test**<br>Attention: **WAIS digit span**<br>Visuospatial: **WAIS block design**
Notes	**Funding**: This study was supported by the National Natural Science Foundation of China (grant number: 81573148), the Natural Science Foundation of Tianjin Medical University (grant number: 2110-2GW034) and the Tianjin Science and Technology Support Program (grant number: 15ZCZDSY01040).<br>**Compliance:** Compliance with the trial protocol was assessed using self-reported number of days on which capsules were taken, a count of the number of capsules returned and blood biomarkers such as 25-hydroxyvitamin D (25-D) and 1,25-D measured from fasting venous blood samples collected from all participants willing to provide blood at baseline and at the 6-month and 12-month assessments for both groups.

ROB

Bias	Judge	Support for judgement
Random sequence generation (selection bias)	Low	Quote: The study sponsor generated the randomisation sequence with a computer.
Allocation concealment (selection bias)	Unclear	No information
Blinding of participants and personnel (performance bias)	Low	Quote: Placebo group followed the same instruction. Placebo capsules consisted of starch granules were manufactured by the same producers and were identical except for the omission of the active substances under investigation.
Blinding of outcome assessment (detection bias)	Unclear	No information
Incomplete outcome data (attrition bias)	Low	Quote: The difference of dropout rates between the two groups was not statistically significant (vitamin D3 group: 14.0%; placebo group: 5.7%; $\chi2=3.48$, $p=0.06$).
Selective reporting (reporting bias)	Low	Quote: This trial has been registered with trial number ChiCTR-IOR-16009307 (http://www. chictr. org. cn/ showproj. aspx? proj= 15255).
Other bias	Low	No other sources of bias identified

Jorde 2019

Methods	a randomized controlled trial
Participants	Location: conducted in the municipality of Tromsø, Norway,<br>Setting of recruitment and treatment: by mail invited; were screened by phone by one of the study nurses at the Clinical Research Unit at the University Hospital of North Norway.<br>Sample size<br>● Number randomized: 211 in intervention, 211 in placebo<br>● Number completed: 192 in intervention, 182 in placebo<br>Participant baseline characteristics<br>● Age in years, mean (SD): intervention, 51.1 (8.3); placebo, 52.5 (8.5)<br>● Female sex: intervention, 91 (47.4%); placebo, 85 (46.4%)<br>● Cognitive function (CN, MCI) – word test, mean (SD): intervention, 8.8 (1.9); placebo, 8.9 (1.8)<br>**Inclusion criteria**<br>In the seventh survey in 2015/2016 all citizens aged 40 years and above (32591) were invited to participate; with serum 25(OH)D values <42 nmol/L and < 80 years old; **Subjects with previous stroke or transitory ischemic attach (TIA) were included if no apparent mental of physical squelae**.<br>**Exclusion criteria**<br>known granulomatous disease, diabetes, renal stones last five years, systolic blood pressure > 174 mmHg, diastolic blood pressure > 104 mmHg, serum craetinine>130 μmol/L in males and > 120 μmol/L in females, clinical depression, clinical signs of vitamin D deficiency (muscle weakness), serious diseases that would make the subject unfit for participation (clinical evaluation by the first author if in doubt), use of vitamin D supplements exceeding 800 IU vitamin D per day, use of solarium on a regular basis, and planned holiday(s) in tropical areas during the study period. Women of childbearing potential without use of acceptable contraception (hormonal

	or intrauterine device) were excluded. Subjects with other specific neurological diseases were not included.
Interventions	Intervention: **vitamin D (**cholecalciferol, D3) 100,000 IU given as a bolus dose followed by 20,000 IU per week for **four months**<br>Placebo: arachis oil (Ayanda GmbH & CoKG, Falkenhagen, Germany)
Outcomes	At 4 months<br>Memory: **verbal recall test**<br>Processing speed: **Coding test**, Tapping test
Notes	Funding: The present study was supported by grants from the North Norway Regional Health Authorities (grant number SFP1277-16) and UiT The Arctic University of Norway.<br>Compliance: Compliance was calculated as the ratio between capsules used (capsules supplied minus capsules returned) and number of weeks between second and fourth visit.

ROB

Bias	Judge	Support for judgement
Random sequence generation (selection bias)	Low	Quote: The randomization unit assigned the subject a randomization number using a block randomization procedure
Allocation concealment (selection bias)	Low	Quote: This number was sent to the hospital's pharmacy who did have the randomization key and dispensed the medication accordingly.
Blinding of participants and personnel (performance bias)	Low	Quote: identical looking placebo capsules; All others involved in the study, including nurses, doctors and study participants, were blinded throughout the study.
Blinding of outcome assessment (detection bias)	Low	Quote: All others involved in the study, including nurses, doctors and study participants, were blinded throughout the study.
Incomplete outcome data (attrition bias)	Low	Comment: low drop-out rates in both arms
Selective reporting (reporting bias)	Low	Quote: Trial registration: ClinicalTrials.gov NCT02750293
Other bias	Low	Quote: The compliance rate was high, 14.5% of the subjects had a compliance rate between 84.2 and 100%, and the rest had a compliance rate of 100%.

Lee YJ, 2019

Methods	pre-test–post-test design, a nonequivalent control group
Participants	Location: South Korea<br>Setting of recruitment and treatment: between November 28, 2014, and March 7, 2015; had a nonequivalent control group and pre-test–post-test design<br>Sample size<br>● Number randomized: 51 in intervention, 57 in placebo<br>● Number completed: 46 in intervention, 48 in placebo<br>Participant baseline characteristics<br>● Age in years, mean (SD): intervention, 77.8 (6.0); placebo, 76.9 (6.5)

	● Female sex: intervention, 36 (78%); placebo, 39 (81%)<br>● Cognitive function (CN, MCI) – MMSE, mean (SD): intervention, 22.9 (3.8); placebo, 23.8 (3.6)<br>**Inclusion criteria**<br>aged > 65 years, lived alone, had serum 25-hydroxyvitamin D level < 20 ng/mL, and were members of senior centers in S city, Gyeonggi Province.<br>**Exclusion criteria**<br>Serum vitamin D level > 20 ng/mL, history of parathyroid disease or kidney disease, taking vitamin D or calcium supplements, receiving hormone therapy, or history of cardiac disease or **cerebrovascular disease**.
Interventions	Intervention: 1,000 IU vitamin D daily for 12 weeks<br>the minimum period taken for vitamin D supplementation to improve muscle strength without s/e<br>Placebo: no information
Outcomes	At 3 months<br>Global: MMSE
Notes	Funding: no information; We thank the nurses at S city visiting health center who assisted with the survey.<br>Compliance: compliance was checked during the weekly exercise program as well as over phone, once weekly

ROB

Bias	Judge	Support for judgement
Random sequence generation (selection bias)	Low	Quote: Based on a coin toss, four centers were assigned to the experimental vitamin D supplementation group (VDG) and the remaining to the control group (CG).
Allocation concealment (selection bias)	Unclear	Comment: no information
Blinding of participants and personnel (performance bias)	Unclear	Comment: no information
Blinding of outcome assessment (detection bias)	Unclear	Comment: no information
Incomplete outcome data (attrition bias)	Unclear	Comment: no information
Selective reporting (reporting bias)	Unclear	Comment: no information
Other bias	Low	No other sources of bias identified

Owusu, 2019

Methods	Randomized, double-blind, placebo-controlled clinical trial, 36 months duration
Participants	Location: United States<br>Setting of recruitment and treatment: were recruited in lectures by staff at churches, hospital events, and senior centers and through advertisements on flyers, postcards, and letters.<br>Sample size<br>● Number randomized: 130 in intervention, 130 in placebo<br>● Number completed: 95 in intervention, 89 in placebo<br>Participant baseline characteristics

	• Age in years, median (IQR): intervention, 67.8 (65.1-71.5); placebo, 69.0 (65.4-73.4)<br>• Female sex: intervention, 100%; placebo, 100%<br>• Cognitive function (CN, MCI) – MMSE, median (IQR): intervention, 29 (28-30); placebo, 29 (27-30)<br>**Inclusion criteria**<br>Aged 65 or older; self-declared African-American women; Postmenopausal; advised to not take vitamin D–containing supplements for 4 to 6 weeks before the study; with serum 25(OH)D between 8 and 26 ng/mL were included; Ambulatory<br>**Exclusion criteria**<br>osteoporosis of the total hip, MMSE score less than 21, moderate to severe vertebral fractures, liver disease, and kidney stones.
Interventions	Intervention: 2,400 IU, 3,600 IU, or 4,800 IU **vitamin D3,** 1,200mg calcium, 1 tab qd, for 36 months (Doses depended on serum 25(OH)D levels at that visit and were adjusted to achieve and maintain a serum level of 30 ng/mL)<br>Placebo: placebo D3 + 1,200mg calcium
Outcomes	At 6, **12**, 18**, 24,** 30**, 36** months<br>Global: MMSE
Notes	Funding: The PODA trial was funded by the National Institute on Aging (R01-AG032440-05) and NIH Office of Dietary Supplements (ODS) R01-AG032440-01A2.

ROB

Bias	Judge	Support
Random sequence generation (selection bias)	Low	Quote: Once a woman qualified, she was randomized into the placebo or Vitamin D group.<br>Block randomization was performed at baseline using a computer-generated (SAS Proc Plan, SAS Institute, Inc., Cary, NC) randomization list.
Allocation concealment (selection bias)	Unclear	No information
Blinding of participants and personnel (performance bias)	Low	Quote: The placebo and active group doses were titrated in a similar fashion throughout the study to maintain the blind.
Blinding of outcome assessment (detection bias)	Unclear	No information
Incomplete outcome data (attrition bias)	Low	Quote: Any participant who was randomized and received at least 1 dose of study medication was included in the intentionto-treat (ITT) population<br>Missing data were assumed to have occurred at random.<br>Fifty-eight active group participants (44.6%) and 61 placebo group participants (47.3%) had completed college. There was no statistically significant difference in education between groups.
Selective reporting (reporting bias)	Unclear	No information
Other bias	Low	No other sources of bias identified

Rossom 2012

Methods	Post hoc analysis of a randomized double-blind placebo-controlled trial, 96 months duration
Participants	Location: United States<br>Setting of recruitment and treatment: Forty Women's Health Initiative (WHI) clinical centers<br>Sample size<br>● Number randomized: 2,034 in intervention, 2,109 in placebo<br>● Number attended at least once: 2,028 in intervention, 2,094 in placebo<br>Participant baseline characteristics<br>● Age in years, mean: intervention, 70.7; placebo, 70.9<br>● Female sex: intervention, 2,034/2,034 (100%); placebo, 100% 2,109/2,109 (100%)<br>● Cognitive function (CN, MCI) – MMSE, mean (SD): intervention, 95.4 (4.2); placebo, 95.4 (4.1)<br>**Inclusion criteria**<br>Aged 65 or older; women; without probable dementia;<br>**Exclusion criteria**<br>those related to diseases associated with significant risk of mortality (invasive cancer in the previous 10 years; any history of breast cancer or a suspicion of breast cancer at the time of screening; acute myocardial infarction, stroke, or transient ischemic attack in the previous 6 months; known chronic active hepatitis or severe cirrhosis), safety (blood cell count indicative of disease, severe hypertension, current use of oral corticosteroids), and adherence or retention (unwillingness or inability to complete baseline study requirements).
Interventions	Intervention: 400IU **vit D3** + 1,000mg calcium carbonate for 96 months<br>Placebo: identical<br>Use of personal supplemental calcium up to 1,000 mg/day and vitamin D up to 600 IU/day in addition to the study tablets (increased to 1,000 IU/day in 1999) was also allowed.
Outcomes	At **12**, **24**, 36, 48, 60, 72, 84, **96** months<br>**Global:** MMSE
Notes	Funding: The WHI program is funded by the National Heart, Lung, and Blood Institute, National Institutes of Health, U.S. Department of Health and Human Services through Contracts N01WH22110, 24152, 32100–2, 32105–6, 32108–9, 32111–13, 32115, 32118– 32119, 32122, 42107–26, 42129–32, and 44221.

ROB

Bias	Judge	Support
Random sequence generation (selection bias)	Unclear	Quote: were randomly assigned with equal probability to combined calcium and vitamin D or placebo
Allocation concealment (selection bias)	Unclear	No information
Blinding of participants and personnel (performance bias)	Low	Quote: Identical placebo.
Blinding of outcome assessment (detection bias)	Unclear	No information

Incomplete outcome data (attrition bias)	Unclear	No information
Selective reporting (reporting bias)	Unclear	No information
Other bias	Unclear	Quote: In addition, the dose of vitamin D supplementation of 400 IU/day was low and may not have been enough to have an effect on cognitive function. Women in this study were generally not calcium or vitamin D deficient at the beginning of the intervention, so it is not possible to generalize the results of this study to women with calcium or vitamin D deficiency.

CN, cognitively normal; MCI, mild cognitive impairment; ROB, risk of bias

Table S3. Cognitive domains and their corresponding tests used in each study

Cognitive domain	Cognitive tests	Frequency
Global cognitive function	Mini-Mental State Examination	de Jager 2012; Ford 2010; Garcia 2004; Lee HK 2016; Lee YJ 2019; McMahon 2006; Naeini 2014; Owusu 2019; Peterson 2005; Rossom 2012; Ting 2017; van der Zwaluw 2014; van Uffelen 2008
	Modified Mini-Mental State Examination	Yaffe 2004
	Telephone Interview for Cognitive Status (TICS)	Andreeva 2011; Ford 2010; Grodstein 2007; Kang 2006; Kang 2008; Kang 2009; MRC/BHF 2002; Stott 2005; Walker 2012
	Full scale IQ from Wechsler Adult Intelligence Scale (WAIS)	Hu 2018; Ma 2016
	Dementia rating scale	Garcia 2004
	Repeatable Battery of the Assessment of Neuropsychological Status (RBANS)	Moore 2018
	Montreal Cognitive Assessment	Aspell 2017; Jiang 2013
	intelligence (New Adult Reading Test- Nelson and O'Connell, 1978)	Smith 1999
	Composite Z score – total NTB (neuropsychological battery)	Kwok 2017
	Composite Z score – average of the memory, sensorimotor speed, complex speed, information processing speed, and word fluency domains	Durga 2007
Episodic memory	Free recall task	Smith 1999
	Logical Memory Part II (delayed recall) from Wechsler Memory Scale (WMS)	Scott 2017; Yaffe 2004
	15-word learning test, delayed recall	Eussen 2006
	Word list recall from TICS	Walker 2012
	Repetition (%) of TICS	Andreeva 2011
	Words recalled at delayed recall of list A from California Verbal Learning Test (CVLT)	Dangour 2015; Ford 2010; Garcia 2004; Rossom 2012
	Delayed recall from Rey Auditory Verbal Learning Test (AVLT)	McMahon 2006; van der Zwaluw 2014; van Uffelen

		2008
	Recall from Hopkins Verbal Learning Test	de Jager 2012
	Berliner Amnesie Test, pattern recognition	Wolters 2005
	Visual reproduction I from WMS	Lewerin 2005
	Rey complex figure, delayed recall	Eussen 2006
	Benton visual retention test	Rossom 2012
	Visual memory span forward	Ting 2017
	Recognition of two-word nouns	Cheng 2016
	Forget score, a combination of The scores on trial 3 of Associate Learning (AL3) and on Associate Recognition (AR)	Deijen 1992
	Composite Z score: Shopping List Task + continuous paired associates learning	Kwok 2017
	Composite Z score: 15-word learning test 'total immediate recall' + 15-word learning test 'maximum immediate recall' + 15-word learning test 'delayed recall'	Durga 2007
	Composite score (Alzheimer's disease assessment scale [ADAS] immediate and delayed word-recall scores and the New York University immediate and delayed paragraph-recall scores)	Peterson 2005
	Composite score (delayed recall of the TICS 10-word list and the immediate and delayed recalls of the East Boston Memory Test)	Grodstein 2007; Kang 2006; Kang 2008; Kang 2009
Executive function	Category Naming Test; Semantic Verbal Fluency Test; Animal category; verbal fluency	Dangour 2015; de Jager 2012; Durga 2007 (composite Z score); Eussen 2006; Grodstein 2007; Kang 2006; Kang 2008; Kang 2009; McMahon 2006; Rossom 2012; Ting 2017; van der Zwaluw 2014; van Uffelen 2008; Yaffe 2004
	Trail Making Test B; TMT B-A	Aspell 2017; McMahon 2006; Scott 2017; van der Zwaluw 2014
	Stroop Test; Stroop interference; Stroop Color time; Stroop Color-Word time;	Garcia 2004; van der Zwaluw 2014; van Uffelen 2008

	Stroop congruent, response time; Stroop incongruent, response time	
	Frontal assessment battery, total score	Moore 2018; Ting 2017
	Composite Z score: Controlled Oral Word Association Test + category fluency	Kwok 2017
Processing speed	Simple reaction time	Smith 1999
	Digit Symbol Substitution Test score from WAIS-R; Digit symbol coding test from WAIS	Durga 2007 (composite Z score); Hu 2018; Jorde 2019; Lewerin 2005; Scott 2017; Stott 2005; van Uffelen 2008
	symbol letter modality test [number correct]; Symbol Digit Modalities Test, no. correct; Symbol letter modality, n correct in 90 sec	Dangour 2015; Ma 2016; van der Zwaluw 2014
	Finger tapping	Eussen 2006; Rossom 2012
	Composite Z score: simple reaction time + choice reaction time	Kwok 2017
Attention	Trail Making Test A	Eussen 2006; Scott 2017; van der Zwaluw 2014
	Backward Counting task; digit span backward; Digit span [backward + forward] from WAIS	Eussen 2006; Hu 2018; Lewerin 2005; Ma 2016; Rossom 2012; Ting 2017; van der Zwaluw 2014; Yaffe 2004
	digit span forward	Eussen 2006; Lewerin 2005; Ting 2017; van der Zwaluw 2014
	Repeated digits vigilance task	Smith 1999
Visuospatial function	letter cancellation task; digit cancellation	Ford 2010; Ting 2017
	Block design from WAIS-III	Hu 2018; Lewerin 2005; Ma 2016; Scott 2017
	Clock Drawing Test; CLOX 2,	de Jager 2012; Ford 2010; Peterson 2005 (standardized)
	Card rotations	Rossom 2012
	RBANS-index II	Moore 2018
	Chinese character rotation	Cheng 2016
	Rey Complex Figure Test, copy	Eussen 2006

Figure S1. Summary of the risk of bias across seven categories for the overall included studies.

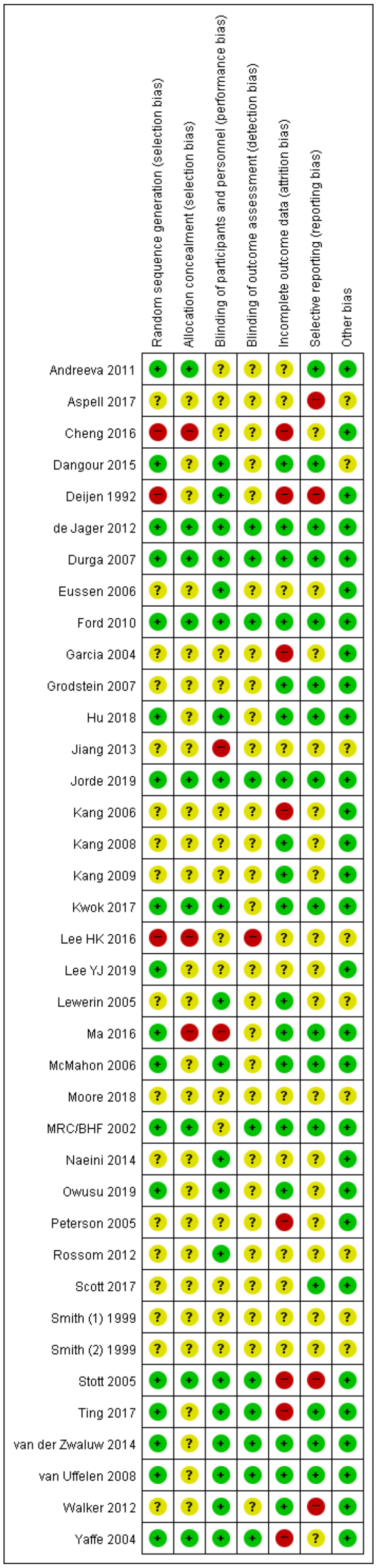

+, low risk of bias; ?, unclear risk of bias; –, high risk of bias

Figure S2. Effect of B vitamins on global cognition in terms of final measurements by (A) the length of the intervention period, (B) the geographic location of the study, and (C) baseline cognitive function

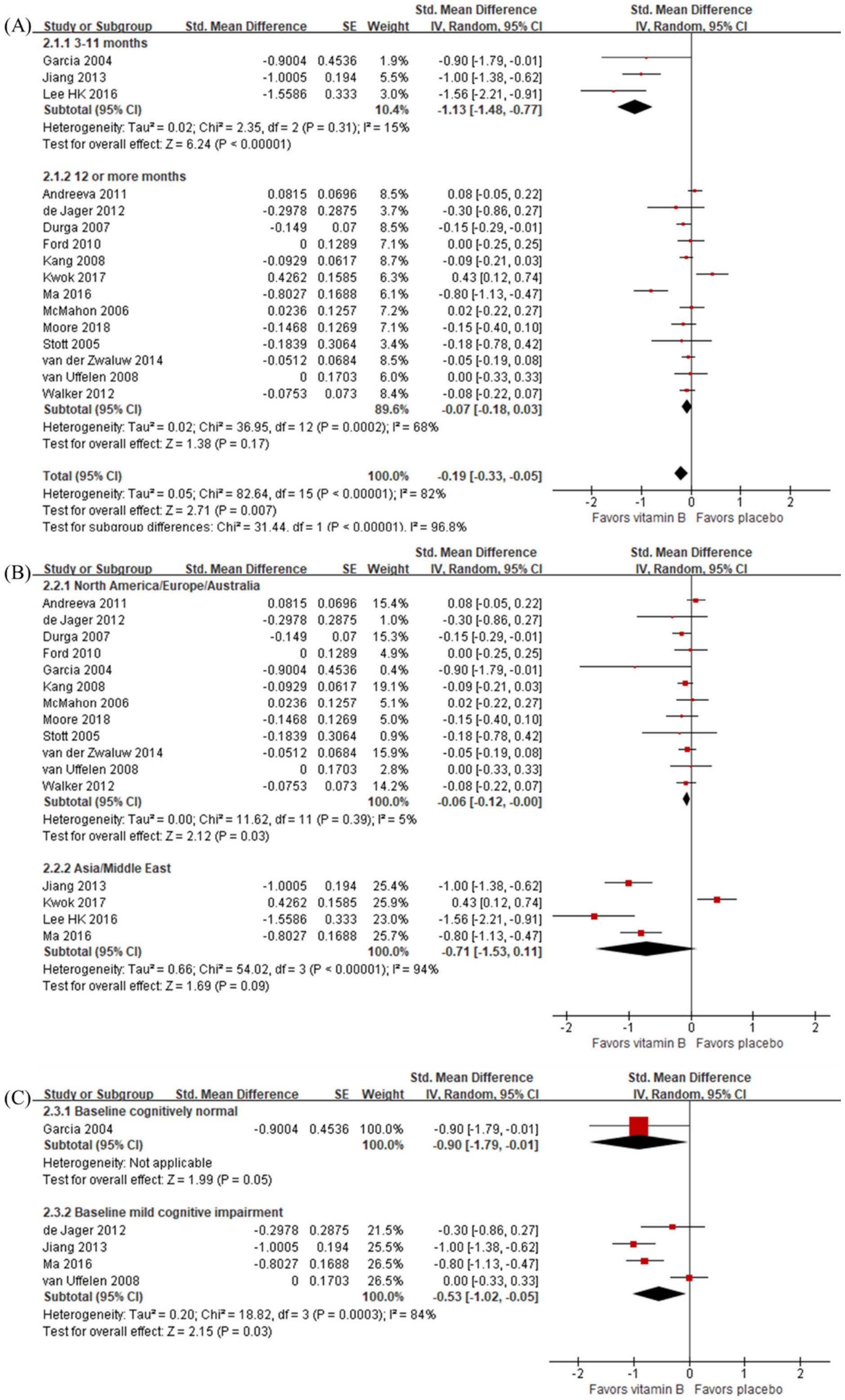

Figure S3. Effects of B vitamins on (A) episodic memory, (B) executive function, (C) processing speed, (D) attention, and (E) visuospatial function in terms of final measurements

(A)

Study or Subgroup	Std. Mean Difference	SE	Weight	Std. Mean Difference IV, Random, 95% CI
Cheng 2016	-0.2797	0.2207	1.7%	-0.28 [-0.71, 0.15]
Dangour 2015	0.0586	0.1475	3.8%	0.06 [-0.23, 0.35]
de Jager 2012	-0.3701	0.2884	1.0%	-0.37 [-0.94, 0.20]
Deijen 1992	0.5108	0.2333	1.6%	0.51 [0.05, 0.97]
Durga 2007	-0.1359	0.07	15.6%	-0.14 [-0.27, 0.00]
Eussen 2006	0	0.1954	2.2%	0.00 [-0.38, 0.38]
Ford 2010	-0.2215	0.1293	4.9%	-0.22 [-0.47, 0.03]
Garcia 2004	-0.4615	0.435	0.4%	-0.46 [-1.31, 0.39]
Kang 2008	-0.1016	0.0617	19.5%	-0.10 [-0.22, 0.02]
Kwok 2017	-0.2076	0.1368	4.4%	-0.21 [-0.48, 0.06]
Lewerin 2005	0.0292	0.1581	3.3%	0.03 [-0.28, 0.34]
McMahon 2006	0.0598	0.1258	5.2%	0.06 [-0.19, 0.31]
Scott 2017	-0.2037	0.1758	2.7%	-0.20 [-0.55, 0.14]
van der Zwaluw 2014	-0.0512	0.0684	16.2%	-0.05 [-0.19, 0.08]
van Uffelen 2008	0.0255	0.1703	2.9%	0.03 [-0.31, 0.36]
Walker 2012	-0.0849	0.073	14.4%	-0.08 [-0.23, 0.06]
**Total (95% CI)**			**100.0%**	**-0.08 [-0.14, -0.03]**

Heterogeneity: Tau² = 0.00; Chi² = 15.65, df = 15 (P = 0.41); I² = 4%
Test for overall effect: Z = 2.84 (P = 0.005)

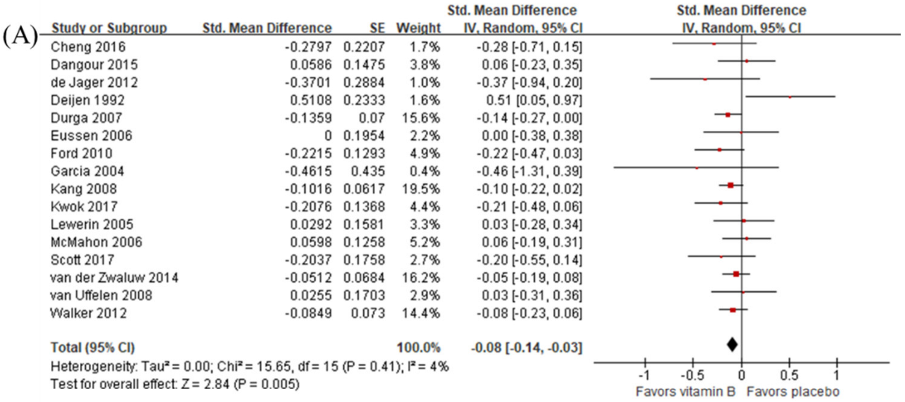

(B)

Study or Subgroup	Std. Mean Difference	SE	Weight	Std. Mean Difference IV, Random, 95% CI
Dangour 2015	-0.1689	0.1477	8.7%	-0.17 [-0.46, 0.12]
de Jager 2012	-0.38	0.2886	6.0%	-0.38 [-0.95, 0.19]
Durga 2007	-0.0383	0.0699	10.0%	-0.04 [-0.18, 0.10]
Eussen 2006	-0.0551	0.1972	7.8%	-0.06 [-0.44, 0.33]
Garcia 2004	0.1293	0.4287	4.0%	0.13 [-0.71, 0.97]
Kang 2008	0.0246	0.0618	10.1%	0.02 [-0.10, 0.15]
Kwok 2017	1.4891	0.149	8.7%	1.49 [1.20, 1.78]
McMahon 2006	0.2126	0.1261	9.1%	0.21 [-0.03, 0.46]
Moore 2018	-0.134	0.1269	9.1%	-0.13 [-0.38, 0.11]
Scott 2017	0.0421	0.1754	8.2%	0.04 [-0.30, 0.39]
van der Zwaluw 2014	0	0.0684	10.0%	0.00 [-0.13, 0.13]
van Uffelen 2008	0.3215	0.1715	8.3%	0.32 [-0.01, 0.66]
**Total (95% CI)**			**100.0%**	**0.13 [-0.08, 0.34]**

Heterogeneity: Tau² = 0.11; Chi² = 106.20, df = 11 (P < 0.00001); I² = 90%
Test for overall effect: Z = 1.18 (P = 0.24)

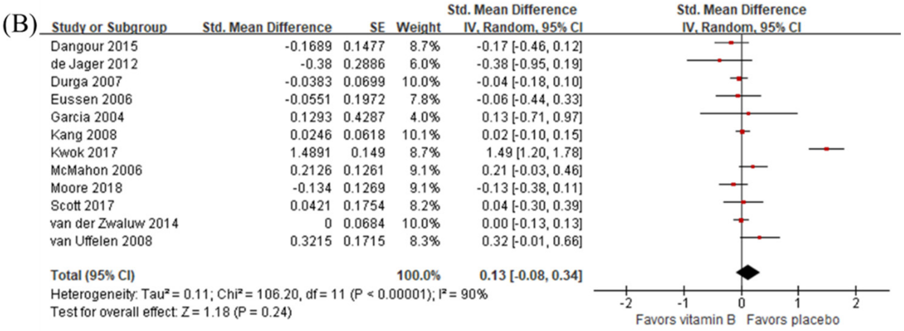

(C)

Study or Subgroup	Std. Mean Difference	SE	Weight	Std. Mean Difference IV, Random, 95% CI
Dangour 2015	0.0451	0.1475	10.3%	0.05 [-0.24, 0.33]
Durga 2007	-0.1578	0.07	16.1%	-0.16 [-0.29, -0.02]
Eussen 2006	-0.0657	0.2088	7.1%	-0.07 [-0.47, 0.34]
Kwok 2017	-0.2746	0.1532	10.0%	-0.27 [-0.57, 0.03]
Lewerin 2005	0.3932	0.1595	9.6%	0.39 [0.08, 0.71]
Ma 2016	-0.5001	0.1648	9.3%	-0.50 [-0.82, -0.18]
Scott 2017	-0.1647	0.1757	8.7%	-0.16 [-0.51, 0.18]
Stott 2005	0.0277	0.3162	3.9%	0.03 [-0.59, 0.65]
van der Zwaluw 2014	0.0512	0.0684	16.2%	0.05 [-0.08, 0.19]
van Uffelen 2008	-0.025	0.1703	9.0%	-0.03 [-0.36, 0.31]
**Total (95% CI)**			**100.0%**	**-0.07 [-0.21, 0.07]**

Heterogeneity: Tau² = 0.03; Chi² = 22.74, df = 9 (P = 0.007); I² = 60%
Test for overall effect: Z = 0.98 (P = 0.33)

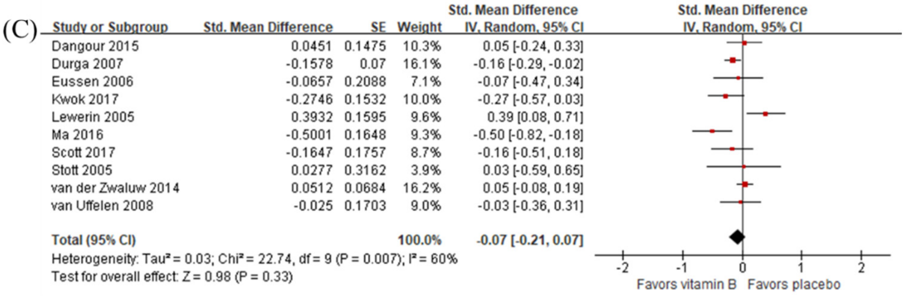

(D)

Study or Subgroup	Std. Mean Difference	SE	Weight	Std. Mean Difference IV, Random, 95% CI
Eussen 2006	-0.0618	0.1962	16.0%	-0.06 [-0.45, 0.32]
Lewerin 2005	0.1665	0.1562	19.5%	0.17 [-0.14, 0.47]
Ma 2016	-0.5549	0.1654	18.7%	-0.55 [-0.88, -0.23]
Scott 2017	0.0893	0.1754	17.8%	0.09 [-0.25, 0.43]
van der Zwaluw 2014	0	0.0684	28.0%	0.00 [-0.13, 0.13]
**Total (95% CI)**			**100.0%**	**-0.07 [-0.29, 0.16]**

Heterogeneity: Tau² = 0.04; Chi² = 12.33, df = 4 (P = 0.02); I² = 68%
Test for overall effect: Z = 0.58 (P = 0.56)

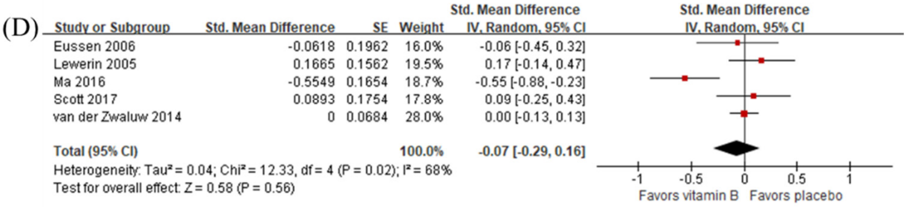

(E)

Study or Subgroup	Std. Mean Difference	SE	Weight	Std. Mean Difference IV, Random, 95% CI
Cheng 2016	-0.3014	0.2208	7.7%	-0.30 [-0.73, 0.13]
de Jager 2012	0	0.2858	4.8%	0.00 [-0.56, 0.56]
Eussen 2006	-0.1794	0.2056	8.8%	-0.18 [-0.58, 0.22]
Ford 2010	0.1802	0.1291	19.6%	0.18 [-0.07, 0.43]
Lewerin 2005	0.1913	0.159	13.9%	0.19 [-0.12, 0.50]
Ma 2016	-0.0371	0.1622	13.4%	-0.04 [-0.36, 0.28]
Moore 2018	-0.1787	0.127	20.1%	-0.18 [-0.43, 0.07]
Scott 2017	-0.0564	0.1754	11.7%	-0.06 [-0.40, 0.29]
**Total (95% CI)**			**100.0%**	**-0.02 [-0.15, 0.10]**

Heterogeneity: Tau² = 0.00; Chi² = 8.02, df = 7 (P = 0.33); I² = 13%
Test for overall effect: Z = 0.39 (P = 0.70)

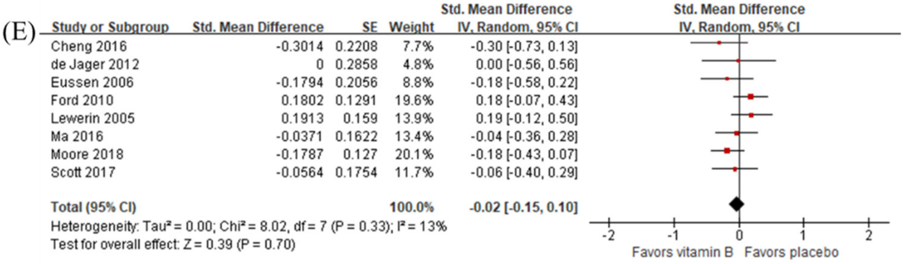

Figure S4. Effect of antioxidant vitamins on (A) episodic memory, (B) executive function, (C) processing speed, and (D) attention in terms of final measurements

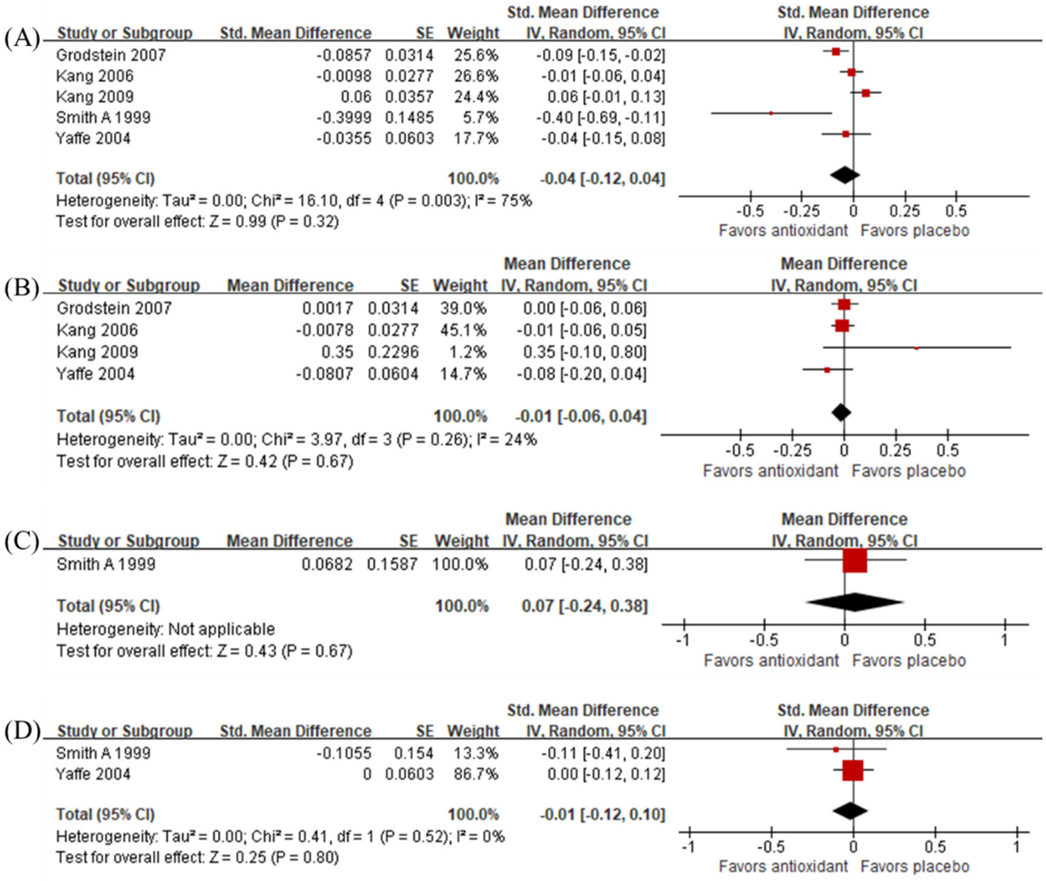

Figure S5. Effect of vitamin D on (A) episodic memory, (B) processing speed, (C) attention, and (D) visuospatial function in terms of final measurement

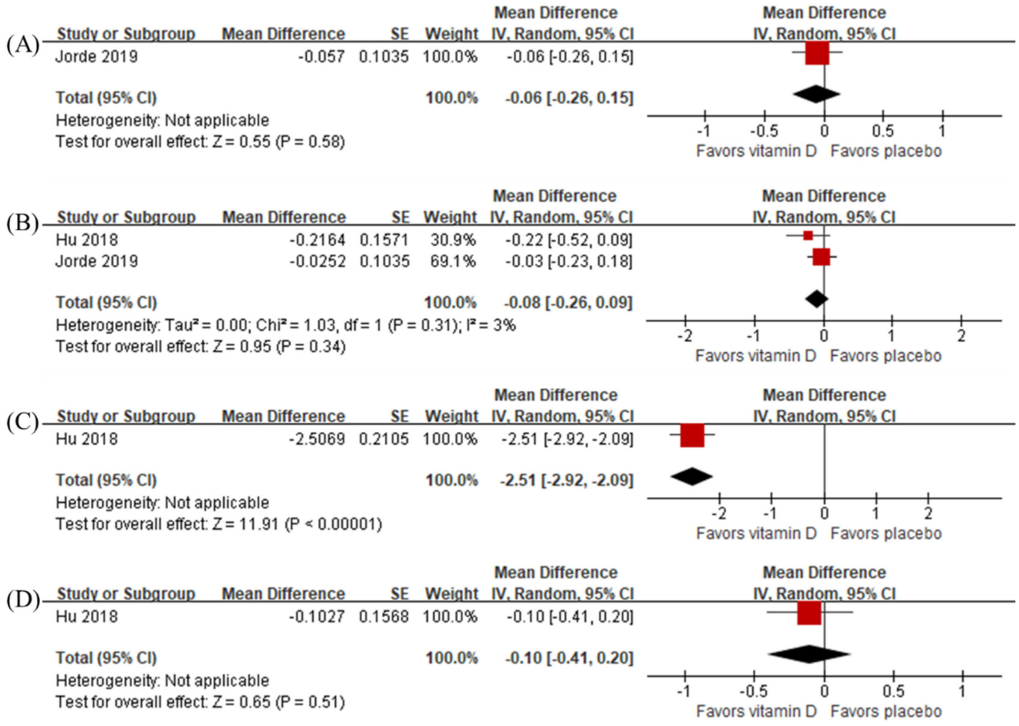

Figure S6. Funnel plots of meta-analyses with 10 or more included studies.

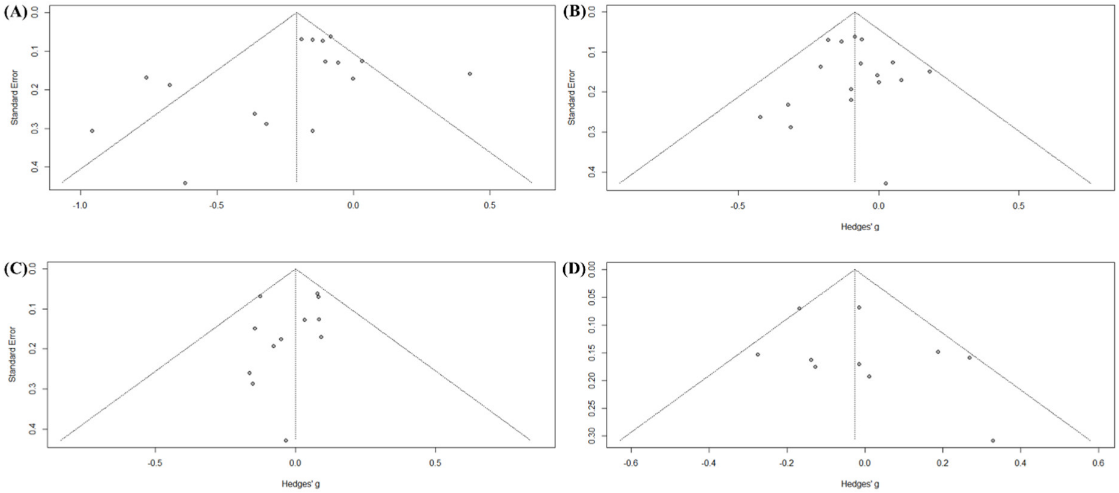

Effects of B vitamins on (A) global cognition, (B) episodic memory, (C) executive function, and (D) processing speed.
